# The rising burden of Alzheimer’s and other dementias: role of high fasting plasma glucose from 1990 to 2021

**DOI:** 10.3389/fmed.2025.1592620

**Published:** 2025-06-26

**Authors:** Shuhua Liu, Yuxuan Wu, Fangying Chen, Luying Han, Yu Zhang, Enqiang Chang

**Affiliations:** ^1^Department of Anesthesiology, Guangxi Medical University Cancer Hospital, Guangxi Clinical Research Center for Anesthesiology, Guangxi, China; ^2^Department of Anesthesiology and Perioperative Medicine, People’s Hospital of Zhengzhou University, Henan Provincial People’s Hospital, Zhengzhou, Henan, China; ^3^Department of Anesthesiology and Perioperative Medicine, People's Hospital of Henan University, Henan Provincial People’s Hospital, Zhengzhou, Henan, China; ^4^Division of Anesthetics, Pain Medicine and Intensive Care, Department of Surgery and Cancer, Faculty of Medicine, Imperial College London, Chelsea and Westminster Hospital, London, United Kingdom

**Keywords:** Alzheimer’s disease and other dementias, prevalence, incidence, disability-adjusted life years, high fasting plasma glucose, 60 to 74 age groups

## Abstract

**Background:**

Dementia, one of the top 10 causes of death globally, imposes significant health and socioeconomic/socioeconomic burdens, with prevalence projected to reach 82 million by 2030. High fasting plasma glucose (HFPG) is a prominent modifiable risk factor for dementia in 2021. This study aims first to examine the global trend in dementia burden and the disability-adjusted life years and death attributable to HFPG from 1990 to 2021 and second to define age-specific disparities in dementia burden among older populations.

**Methods:**

Using data from the Global Burden of Diseases Study (GBD) 2021, this research evaluated the incidence, prevalence, deaths, disability-adjusted life years (DALYs), and HFPG-attributable burden related to Alzheimer’s disease and other dementias (ADOD). The estimated annual percentage change was calculated to qualify the burden change of ADOD.

**Results:**

There was a significant rise in the ADOD burden globally, with over 56.9 million prevalent cases and 2.0 million deaths in 2021. the incidence and prevalence were positively correlated with HFPG-related summary exposure value. The HFPG-attributable ADOD burden has increased worldwide over time. Globally, the 60 to 74 age groups suffered a prominent rise in the burden and HFPG-attributable burden of ADOD.

**Conclusion:**

The global burden and HFPG-attributable ADOD burden have remained prominent and have increased increase over the past 32 years. The ASIR and ASPR showed positive correlations with the SEV related to HFPG. Notably, the 60 to 74 age groups suffered a prominent rise in burden and HFPG attributable to the DALYs rate of ADOD over time. Moreover, a prominent positive correlation was observed between the incidence and prevalence rate with the SEVs related to HFPG occurred in the population aged 60 to 74 years old. Therefore, HFPG should be emphasized in strategic priorities for controlling the ADOD burden.

## Introduction

Dementia is one of the top 10 death causes in 2021 worldwide and is recognized as a public health priority by WHO; it has become the most significant global challenge for health and social care in the 21st century (1–3). Alzheimer’s disease and other dementias (ADOD) became the second cause of adult disability among those aged 60 years and older in 2021 (3, 4). Worldwide, the number of people living with dementia has increased from 20.3 million in 1990 to about 47 million in 2015, and the total number of people with dementia is projected to reach 82 million in 2030 (3, 5). Dementia leads to increased costs for governments, communities, families, and individuals and a loss of productivity for economies. In 2015, the global dementia-related costs were estimated to be US $818 billion, equivalent to 1.1% of global gross domestic product. The costs will be US $2.54 trillion in 2030 and US $9.12 trillion in 2050 (5, 6).

While there is no effective treatment for dementia, previous studies reported that modifiable risk factor reduction might prevent up to 40% of dementia (7). *Dementia prevention, intervention, and care: The 2020 report of the Lancet Commission* published 12 modifiable risk factors for dementia (5, 6). The Global Burden of Diseases Study (GBD) 2021 Nervous System Disorders Collaborators reported that high fasting plasma glucose contributed the largest all-age population attributable fraction for ADOD than high body-mass index (BMI) and smoking in 2021 (4). Yang *et al.* also reported that high fasting plasma glucose was the leading risk factor for ADOD compared to high BMI and smoking in China; similar discrepancies were observed in all Chinese municipalities in 2021 (8). Dementia predominantly occurs in older populations (7); Jia *et al*. also demonstrated that Diabetes contributed to a higher risk for dementia than smoking, hypertension, hyperlipidemia, and heart disease for older adults in China (9). Moreover, Diabetes is also one of the top 10 causes of death in 2021, with a sharp percentage increase of 95% since 2000 (1). Therefore, it is urgent to reduce the exposure of dementia modifiable risk factors for controlling the disease burden of ADOD, especially in Diabetes or high fasting plasma glucose (HFPG). Moreover, Japanese surveys have observed an upward trend or stability in the incidence of dementia among various elderly populations over the past several decades (10). Age differences in the change of dementia burden for the elderly population have not received sufficient attention globally; there is a lack of related research to explain this phenomenon and adequately address this challenging situation (1, 4, 8–10). Therefore, the primary objective of our study is to analyze the latest global trends in the incidence, prevalence, deaths, DALYs, and HFPG-attributable burden related to ADOD, as well as age-specific disparities in dementia burden among older populations.

## Methods

### Data sources

Raw data were downloaded from the Global Burden of Diseases, Injuries, and Risk Factors Study (GBD) 2021^1^, which comprehensively updated and evaluated epidemiological data on 371 diseases and injuries and 88 risk factors in 204 countries and territories from 1990 to 2021 (11). The detailed methodology used by GBD 2021 was extensively described in previous GBD studies (4, 12). The waiver of informed consent has been approved by the University of Washington Institutional Review Board due to the use of deidentified aggregated data in GBD 2021.

### Definitions

Alzheimer’s disease and other dementias (ADOD) were defined based on the 3rd, 4th, or 5th editions of the Diagnostic and Statistical Manual of Mental Disorders or International Classification of Diseases (ICD) case criteria. The diagnosis was accomplished using clinical records, algorithm criteria, National Institute on Aging Alzheimer’s disease criteria, 10/66 algorithm criteria, and general practitioner records. The ICD codes for ADOD are as follows: 290, 291.2, 291.8, 294, and 331 in the ICD-9, and F00, F01, F02, F03, G30, and G31 in the GBD 2021 (4, 8).

The Socio-Demographic Index (SDI) is a composite measure that quantifies a country’s or region’s development level based on fertility rates, education levels, and per capita income data for a given year. It ranges from 0 (worst) to 1 (best) and divided 204 countries and regions into five SDI categories: high SDI (>0.81), high-middle SDI (0.71–0.81), middle SDI (0.62–0.71), low-middle SDI (0.47–0.62) and low SDI (<0.47) (4, 12). In addition, GBD 2021 classified regions into seven super-regions and 21 sub-regions based on epidemiological similarity and geographical proximity. The details of region classification are provided in published GBD studies (4, 12).

High fasting plasma glucose (HFPG) is any level above the theoretical minimum-risk exposure level (TMREL), which is 4.9–5.3 mmol/L. High body-mass index (BMI) for adults (ages 20 and above) is defined as BMI greater than 20 to 23 kg/m^2^. High BMI for children and adolescents (ages 2–19) is known as being overweight or obese based on International Obesity Task Force standards. GBD study defines smoking as the prevalence of current smoking and the prevalence of former smoking using data from relevant surveys and studies. Current smokers are defined as individuals who currently use any smoked tobacco product on a daily or occasional basis; former smokers are defined as individuals who quit using all smoked tobacco products for at least 6 months, where possible, or according to the definition used by the given survey (12).

### Measures

The GBD 2021 extracted vast amounts of data from national disease surveillance systems, maternal and child health surveillance websites, vital registration systems, and verbal autopsy data, among others. The data was subjected to rigorous and cautious adjustments to ensure accuracy before analysis. The DisMod-MR 2.1 (disease-model-Bayesian meta-regression) tool is used to calculate incidence and prevalence (4, 8). The Cause of Death Ensemble modeling (CODEm) framework was applied to estimate ADOD mortality. Multiple models were employed to minimize differences in study design and methodology across various data sources, ensuring consistent and accurate calculations of ADOD incidence, prevalence, and mortality. The Years Lived with Disability (YLDs) is defined as a measure that quantifies the burden of living with the effects of ADOD. The Years of life lost (YLLs) were calculated as the standard life expectancy at the age of death multiplied by the number of deaths (4, 8, 12). The sum of YLLs and YLDs due to ADOD was considered disability-adjusted life-years (DALYs). Our study obtained the following annual information from 1990 to 2021, based on the GBD 2021, including the number and rate of ADOD prevalence, incidence, deaths, DALYs, YLDs, and YLLs by sex and age, for global, regional, and 204 countries and territories. Age groups were extracted from 40 years to 95 + years at 5-year intervals.

### Risk factor

The GBD 2021 used a comparative risk assessment (CRA) method to evaluate the ADOD burden attributable to different risk factors (4, 8, 12). Three risk factors of ADOD were classified into behavioral (smoking) and metabolic risks (HFPG, high BMI). According to epidemiological evidence, GBD 2021 established the theoretical minimum risk exposure level (TMREL) for each risk factor, indicating the counterfactual exposure level that minimizes health risk. Summary exposure values (SEVs) are calculated as the age-specific risk-weighted prevalence of corresponding risk exposure. SEVs are evaluated on a 0 to 100 scale, with 0 representing the entire population exposed at the TMREL and 100 representing the whole population exposed at the maximum risk exposure level. Population-attributable fractions (PAFs) were evaluated based on relative risk data, exposure data, and the TMREL of risk factors exposure. The PAFs attributable to the corresponding risk factor include all disease burdens that can be directly or indirectly attributed to that risk factor (4, 8, 12). GBD 2021 estimates the ADOD burden attributable to the corresponding risk factors, which means the proportion of ADOD burden attributable to that risk factor, as quantified by the product of the PAF and the DALYs or deaths associated with the outcome. According to GBD 2021, our study also obtained the attributable measure (rate, number, and PAFs) of ADOD burden related to each risk factor (death, DALYs, YLDs, YLLs) and SEVs of HFPG, high BMI, and smoking by sex and age (age groups are same as above) in global, regions, countries and territories level from 1990 to 2021.

### Data analysis

Age-standardized rates (ASRs per 100,000 population) of incidence (ASIR), prevalence (ASPR), death (ASMR), and DALYs (ASYR) have been used to describe the universal indicators to compare the ADOD burden in different regions/populations among the same population at different times (4, 8, 12). GBD 2021 presents 95% uncertainty intervals (UIs) for ASRs based on the 2.5th and 97.5th percentiles of 1,000 drawn from the posterior distribution. Our study calculated the Estimated Annual Percentage Change (EAPC) to assess the temporal trends in ASRs of ADOD from 1990 to 2021. A linear regression model was used to calculate the EAPC of ASRs during a specified period. The formula is as follows: Y = *α* + *β*X + e, where Y means the natural logarithm of the ASRs, X indicates the calendar year, α means the intercept term, β denotes the slope or trend, and e is the error term. The EAPC is calculated as 100 × [exp(β)–1], meaning the annual percentage change. The model also calculated the corresponding 95% confidence interval (CI). EAPC>0 and 95% UI > 0 represented an uptrend in ASRs, whereas EAPC<0 and 95% UI < 0 indicated a downtrend in ASRs. Otherwise, ASRs were regarded as insignificant or stable changes during the period (4, 8, 12). Our study computed the Pearson correlation coefficient (R) to identify the correlation between the ADOD burden with SDI or SEVs related to HFPG using R 4.1.2. *p* value<0.05 was considered as the statistical significance.

## Results

### The substantial burden of ADOD in global and different regions

Globally, with a total of 56.86 (95% UI: 49.38 to 64.98) million prevalent individuals and 0.84 (95% UI: 8.62 to 11.16) million new numbers in 2021, a staggering increase from 1990. The number of DALYs and death due to ADOD each sharply increased from 1990 to 2021. The ASRs (per 100,000 population) for prevalence, incidence, DALYs, and deaths have shown flat changes in the study (Supplementary Table 1).

**Figure 1:**
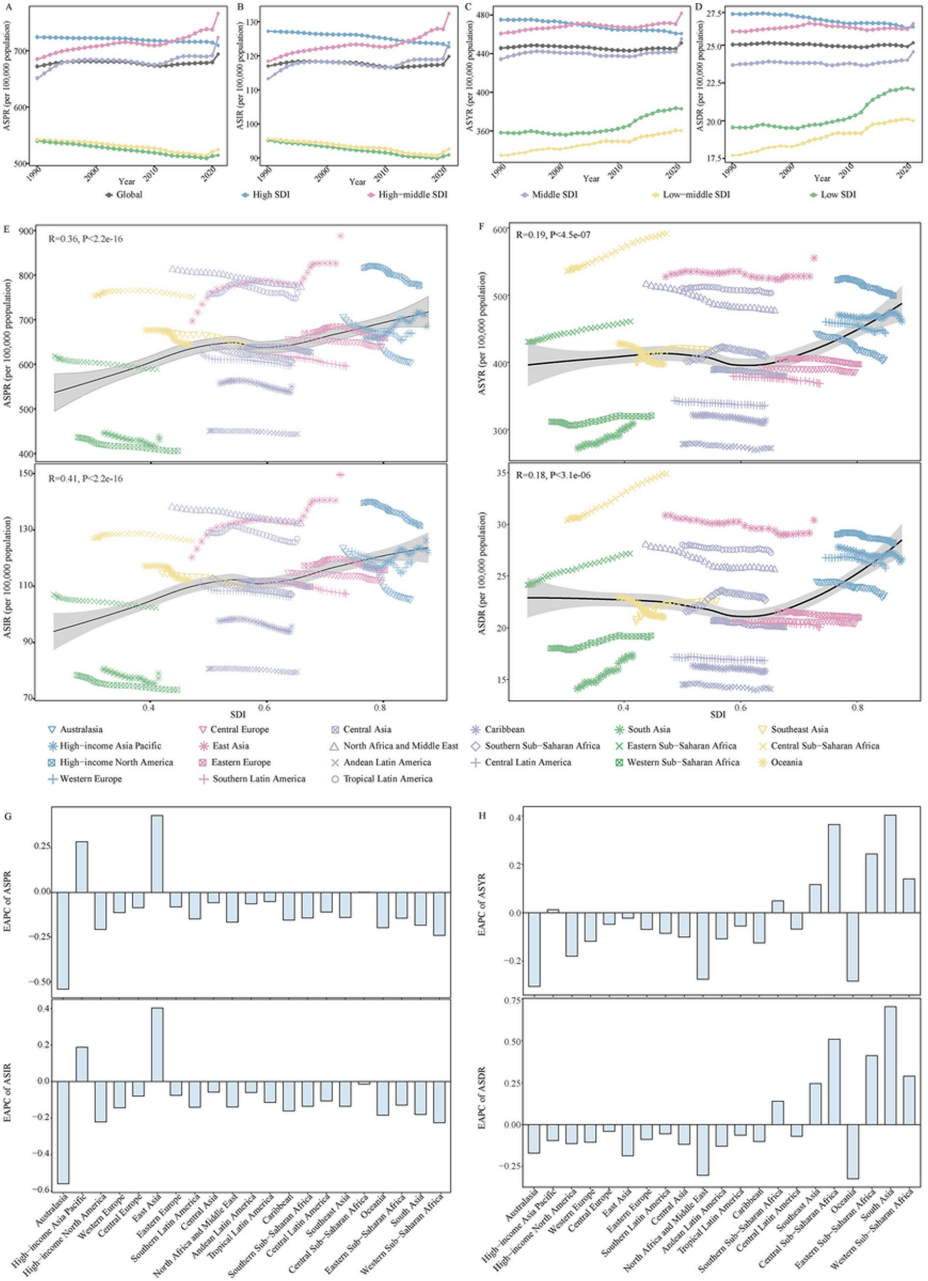
The change in disease burden for Alzheimer’s disease and other dementias in global and regions from 1990 to 2021. **(A)** The trend in ASPR of Alzheimer’s disease and other dementias in global and different SDI regions from 1990 to 2021. **(B)** The trend in ASIR of Alzheimer’s disease and other dementias in global and different SDI regions from 1990 to 2021. **(C)** The trend in ASYR of Alzheimer’s disease and other dementias in global and different SDI regions from 1990 to 2021. **(D)** The trend in ASDR of Alzheimer’s disease and other dementias in global and different SDI regions from 1990 to 2021. **(E)** The correlation between ASPR, ASIR of Alzheimer’s disease and other dementias with SDI in 21 GBD regions from 1990 to 2021. **(F)** The correlation between ASYR, ASDR of Alzheimer’s disease and other dementias with SDI in 21 GBD regions from 1990 to 2021. **(G)** The change in ASPR and ASIR for alzheimer’s disease and other dementias in 21 GBD regions from 1990 to 2021. **(H)** The change in ASYR and ASDR for alzheimer’s disease and other dementias in 21 GBD regions from 1990 to 2021. SDI, socio-demographic index; DALYs, disability adjusted of life years; ASPR, age-standardized prevalence rate; ASIR, age-standardized incidence rate; ASYR, age-standardized DALYs rate; ASDR, age-standardized death rate; GBD, Global Burden of Disease; EAPC, estimated annual percentage change.

The disease burden related to ADOD demonstrated notable disparities among 5 SDI regions. All SDI regions demonstrated uptrends in the number of ADOD burden, and the high SDI region experienced the highest numbers in the study (Supplementary Table 1). ASPR, ASIR, ASYR, and ASDR increased as SDI (Figures 1A–F). However, the highest ASPR, ASIR, ASYR, and ASDR were transferred from the high SDI region in 1990 to the high-middle SDI region in 2021 (Figures 1A–D; Supplementary Table 1). Lower SDI regions have the relatively lowest ASRs over time, and prominent uptrends in ASDR and ASYR were observed in lower SDI regions from 1990 to 2021 (Figures 1A–D; Supplementary Table 1). Meanwhile, the high-middle SDI region and middle SDI region showed a rise in ASPR and ASIR (Figures 1A,B; Supplementary Table 1). The high SDI region showed a decrease in ASRs of ADOD burden over the study period (Figures 1A–D; Supplementary Table 1).

The GBD database divides the world into 21 geographical regions. All GBD regions showed a significant increase in the number of ADOD burden from 1990 to 2021(Supplementary Table 1). Meanwhile, a decline in the corresponding ASRs occurred in most of the 21 regions (Figures 1G,H; Supplementary Table 1). East Asia has the fastest increase in ASPR and ASIR (Figures 1G,H; Supplementary Table 1). The East Asia region had the highest number of prevalence, incidence, DALYs, and death in 2021; the highest ASPR and ASIR due to ADOD occurred in East Asia. Central Sub-Saharan Africa and East Asia showed the highest ASYR and ASDR in 2021 (Supplementary Table 1).

Supplementary Table 2 shows the variations in ASRs of ADOD burden across 204 countries and territories. China had the highest ASPR and ASIR in 2021. Democratic Republic of the Congo, Gabon, Afghanistan, Congo, Angola, Central African Republic, China, and Equatorial Guinea exhibited the highest ASYR and ASDR in 2021. During the period, ASRs of ADOD burden increased in most countries and territories, but China and Taiwan (Province of China) showed the most pronounced increase in ASPR and ASIR.

### A more prominent rise of ADOD burden for 60 to 74 age groups in global and higher SDI regions

**Figure 2:**
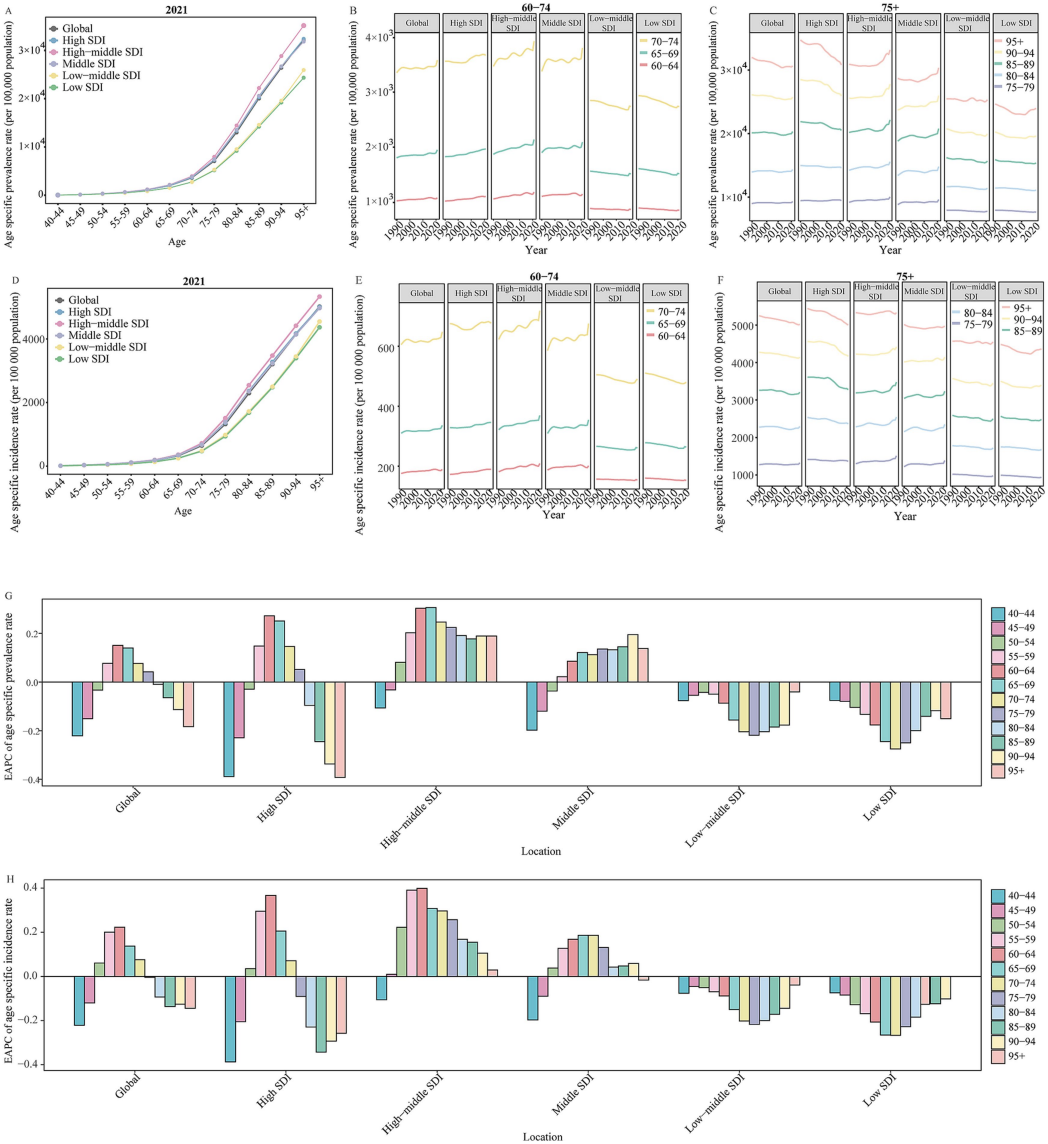
The change in prevalence and incidence rate for Alzheimer’s disease and other dementias by age in global and SDI regions from 1990 to 2021. **(A)** The age specific prevalence rate of Alzheimer’s disease and other dementias by age in global and different SDI regions in 2021. **(B)** The change in age specific prevalence rate of Alzheimer’s disease and other dementias for 60 to 74 age groups in global and different SDI regions from 1990 to 2021. **(C)** The change in age specific prevalence rate of Alzheimer’s disease and other dementias for people aged 75 years and over in global and different SDI regions from 1990 to 2021. **(D)** The age specific incidence rate of Alzheimer’s disease and other dementias by age in global and different SDI regions in 2021E. The change in age specific incidence rate of Alzheimer’s disease and other dementias for 60 to 74 age groups in global and different SDI regions from 1990 to 2021. F. The change in age specific incidence rate of Alzheimer’s disease and other dementias for people aged 75 years and over in global and different SDI regions from 1990 to 2021. **(G)** The EAPC of age specific prevalence rate of Alzheimer’s disease and other dementias by age in global and different SDI regions from 1990 to 2021. **(H)** The EAPC of age specific incidence rate of Alzheimer’s disease and other dementias by age in global and different SDI regions from 1990 to 2021. SDI, socio-demographic index; EAPC, estimated annual percentage change.

**Figure 3:**
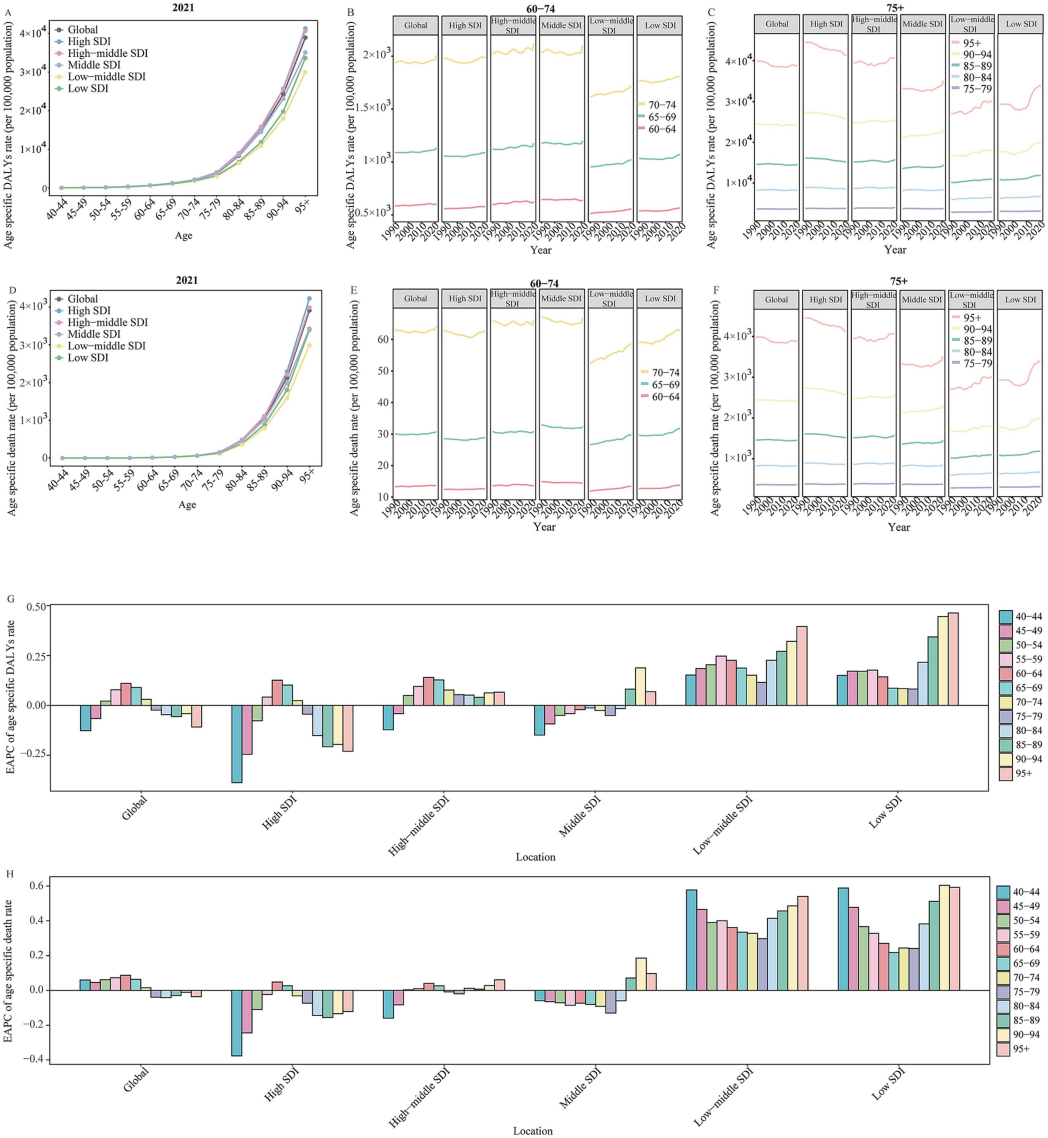
The change in DALYs and death rate for Alzheimer’s disease and other dementias by age in global and SDI regions from 1990 to 2021. **(A)** The age specific DALYs rate of Alzheimer’s disease and other dementias by age in global and different SDI regions in 2021. **(B)** The change in age specific DALYs rate of Alzheimer’s disease and other dementias for 60 to 74 age groups in global and different SDI regions from 1990 to 2021. **(C)** The change in age specific DALYs rate of Alzheimer’s disease and other dementias for people aged 75 years and over in global and different SDI regions from 1990 to 2021. **(D)** The age specific death rate of Alzheimer’s disease and other dementias by age in global and different SDI regions in 2021. **(E)** The change in age specific death rate of Alzheimer’s disease and other dementias for 60 to 74 age groups in global and different SDI regions from 1990 to 2021. **(F)** The change in age specific death rate of Alzheimer’s disease and other dementias for people aged 75 years and over in global and different SDI regions from 1990 to 2021. **(G)** The EAPC of age specific DALYs rate of Alzheimer’s disease and other dementias by age in global and different SDI regions from 1990 to 2021. **(H)** The age specific death rate of Alzheimer’s disease and other dementias by age in global and different SDI regions in 2021. SDI, socio-demographic index; EAPC, estimated annual percentage change; DALYs, disability-adjusted life-years.

Among all global and SDI regions, the ADOD burden was consistently higher in females compared to males (Supplementary Table 3). The age-specific rate of ADOD burden increased with age, particularly in the 95 + age group (Figures 2, 3). The population aged 60 years and older contributed the majority of the total burden of ADOD (Figures 2, 3). Among the population aged 60 and over, the rise in incidence and prevalence rates occurred in high-middle SDI regions, and prominent uptrends in DALYs and death rates for these population were found in lower SDI regions over the study period (Figures 2, 3). Notably, the population aged 60 to 74 has a more significant rise in prevalence and incidence rate compared to the 75 + age group in global and higher SDI regions over the past 32 years (Figures 2G,H). Meanwhile, the 60 to 74 age groups has a more prominent rise in the DALY rate than the 75 + age groups in the global and high-middle SDI region (Figure 3G).

### A prominent increase in HFPG-attributable ADOD burden in global and all regions

**Figure 4:**
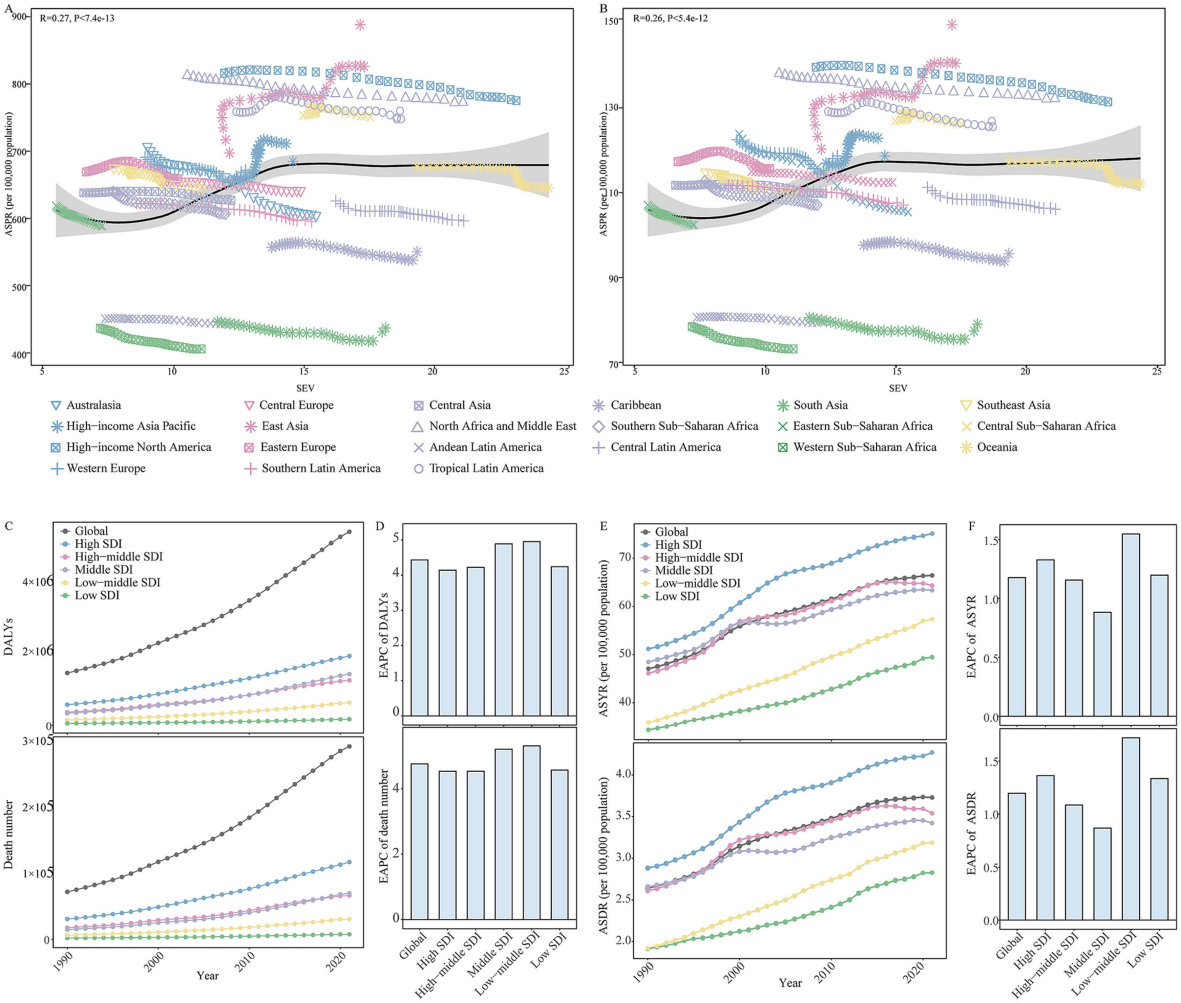
The changes in high fasting plasma glucose contributed to the Alzheimer’s disease and other dementias burden in global and different regions from 1990 to 2021. **(A)** The correlations between ASPR and SEV related to high fasting plasma glucose in 21 GBD regions from 1990 to 2021. **(B)** The correlations between ASIR and SEV related to high fasting plasma glucose in 21 GBD regions from 1990 to 2021. **(C)** The change in high fasting plasma glucose contributed to the DALYs for Alzheimer’s disease and other dementias in global and different SDI regions from 1990 to 2021. **(D)** The EAPC of DALYs related to high fasting plasma glucose in global and different SDI regions from 1990 to 2021. **(E)** The change in high fasting plasma glucose contributed to the ASYR and ASDR for Alzheimer’s disease and other dementias in global and different SDI regions from 1990 to 2021. **(F)** The EAPC of the ASYR and ASDR related to high fasting plasma glucose in global and different SDI regions from 1990 to 2021. SEV, summary exposure value; ASPR, age-standardized prevalence rate; ASIR, age-standardized incidence rate; GBD, Global Burden of Disease; DALYs, disability-adjusted life-years; ASYR, age-standardized DALYs rate; ASDR, age-standardized death rate; GBD, Global Burden of Disease; SDI, socio-demographic index; EAPC, estimated annual percentage change.

As shown in Supplementary Table 4, HFPG was the leading risk factor that contributed to ADOD burden in both female and male compared to high BMI and smoking in 2021. The number, PAFs, and ASRs of HFPG-attributable DALYs and death have sharply increased over the study period (Table 1). Moreover, statistically significant positive correlations were observed in the ASIR (R = 0.26, *p* < 0.01) and ASPR (R = 0.27, *p* < 0.01) with SEV related to HFPG, respectively (Figures 4A,B).

**Table 1:** The change of high fasting plasma glucose contributed to Alzheimer’s disease and other dementias burden in global and different regions from 1990 to 2021.

Location	Number×10^3^ (95% UI)		EAPC of number (95% CI)	PAFs (%, 95 UI)		EAPC of PAF (95% CI)	ASR (per 100,000 population) (95% UI)		EAPC of ASR (95% CI)
	1990	2021		1990	2021		1990	2021	
DALYs									
Global	1441.54 (82.70, 3878.46)	5348.85 (308.06, 14351.16)	4.43 (4.38, 4.48)	10.50 (0.89, 21.20)	14.64 (1.21, 29.44)	1.19 (1.10, 1.29)	47.07 (2.72, 126.46)	66.42 (3.83, 178.85)	1.18 (1.09, 1.26)
High SDI	565.75 (33.90, 1492.49)	1910.98 (112.98, 5105.65)	4.14 (4.05, 4.22)	10.71 (0.94, 21.08)	16.20 (1.35, 32.16)	1.44 (1.33, 1.55)	51.18 (3.07, 135.29)	75.11 (4.45, 198.51)	1.33 (1.22, 1.44)
High-middle SDI	357.82 (20.58, 978.89)	1237.59 (73.04, 3272.97)	4.22 (4.13, 4.30)	9.94 (0.84, 20.12)	13.28 (1.11, 26.44)	1.09 (0.96, 1.22)	46.12 (2.66, 124.91)	64.33 (3.80, 170.88)	1.16 (1.03, 1.28)
Middle SDI	327.92 (17.81, 901.71)	1411.11 (80.07, 3799.73)	4.88 (4.82, 4.95)	11.10 (0.92, 22.99)	13.82 (1.13, 28.27)	0.85 (0.77, 0.94)	48.47 (2.64, 133.72)	63.35 (3.60, 172.97)	0.88 (0.81, 0.95)
Low-middle SDI	142.66 (7.89, 378.74)	622.13 (33.44, 1710.19)	4.95 (4.93, 4.97)	10.67 (0.87, 22.00)	15.84 (1.27, 32.41)	1.31 (1.27, 1.34)	35.92 (1.99, 95.66)	57.36 (3.09, 158.52)	1.55 (1.52, 1.58)
Low SDI	45.68 (2.47, 125.24)	162.09 (8.24, 452.61)	4.24 (4.18, 4.30)	9.55 (0.77, 19.79)	12.86 (1.01, 26.77)	0.96 (0.93, 0.99)	34.41 (1.86, 93.92)	49.47 (2.52, 139.57)	1.20 (1.16, 1.23)
Deaths									
Global	71.47 (2.85, 221.70)	290.03 (11.76, 916.71)	4.76 (4.70, 4.82)	10.48 (0.89, 21.03)	14.70 (1.21, 29.41)	1.22 (1.12, 1.32)	2.64 (0.11, 8.38)	3.73 (0.15, 11.84)	1.20 (1.10, 1.29)
High SDI	30.55 (1.28, 95.70)	116.11 (4.82, 364.12)	4.53 (4.44, 4.62)	10.57 (0.93, 20.65)	16.15 (1.33, 31.95)	1.48 (1.37, 1.60)	2.88 (0.12, 9.17)	4.27 (0.18, 13.27)	1.36 (1.25, 1.48)
High-middle SDI	17.56 (0.70, 55.92)	65.96 (2.68, 205.26)	4.54 (4.44, 4.63)	10.01 (0.84, 20.14)	13.29 (1.10, 26.50)	1.07 (0.93, 1.20)	2.61 (0.10, 8.38)	3.54 (0.14, 11.11)	1.09 (0.94, 1.23)
Middle SDI	14.87 (0.56, 48.06)	69.33 (2.71, 219.02)	5.21 (5.14, 5.28)	11.12 (0.91, 23.18)	13.79 (1.12, 28.33)	0.84 (0.75, 0.92)	2.66 (0.10, 8.58)	3.42 (0.13, 10.92)	0.87 (0.79, 0.94)
Low-middle SDI	6.39 (0.23, 20.48)	30.60 (1.13, 98.70)	5.31 (5.27, 5.35)	10.77 (0.87, 22.27)	15.81 (1.27, 32.33)	1.28 (1.24, 1.31)	1.92 (0.07, 6.09)	3.19 (0.12, 10.43)	1.72 (1.68, 1.76)
Low SDI	2.03 (0.07, 6.57)	7.76 (0.28, 25.00)	4.57 (4.51, 4.63)	9.70 (0.77, 20.27)	12.71 (0.99, 26.31)	0.88 (0.84, 0.92)	1.91 (0.07, 6.19)	2.83 (0.10, 9.31)	1.34 (1.27, 1.40)

DALYs, disability-adjusted life-years; ASR, age-standardized rate; SDI, sociodemographic index; UI, uncertainty intervals; CI, confidence intervals; EAPC, estimated annual percentage change.

At the SDI level, high SDI regions experienced the highest number and ASRs of HFPG-attributable DALYs and death; the second-highest were high-middle and middle SDI regions from 1990 to 2021 (Figures 4C–F). Meanwhile, the highest PAFs of HFPG-attributable DALYs and death were transferred from the middle SDI region to the high SDI region (Table 1). During the period, the number, ASRs, and PAFs of HFPG-attributable DALYs and death exhibited prominent uptrends in all SDI regions (Figures 4C–F; Table 1). The high and low-middle SDI regions experienced the most dramatic increase in ASRs, PAFs of DALYs, and death-related HFPG. The most significant increase in the corresponding number occurred in middle and low-middle SDI regions (Figures 4C–F; Table 1).

Among 21 geographical regions, East Asia exhibited the highest number of DALYs and death attributable to HFPG. High-income North America had the highest ASRs and PAFs in 2021. During the period, the number, ASRs, and PAFs of HFPG-attributable DALYs and death exhibited prominent uptrends in all 21 regions. The most significant increase in number occurred in Andean Latin America, and High-income North America has the most significant increase in ASRs and PAFs (Supplementary Table 5).

The spatial and geographical distribution of HFPG-attributable ADOD burden across 204 countries and territories were exhibited in Supplementary Table 6. Marshall Islands, Tokelau, Afghanistan, Qatar, Morocco, and Iraq showed the highest ASYR and ASDR related to HFPG, Mongolia and Belarus were the lowest in 2021. From 1990 to 2021, over 190 countries and territories experienced uptrends in ASYR and ASDR related to HFPG. Georgia, Greenland, Luxembourg, Uzbekistan, Egypt, and Cambodia showed the most pronounced shifts in HFPG-attributable ADOD burden (Supplementary Table 6).

### A more prominent rise of HFPG-attributable DALYs rate for 60 to 74 age groups in global and most SDI regions

Regarding sex, the number and ASRs of HFPG-attributable DALYs and death were consistently higher in female compared to male in global and most regions, however, the corresponding PAFs for female was lower than male in the period (Supplementary Table 5). Meanwhile, male (ASIR: R = 0.38, *p* < 0.00001; ASPR: R = 0.39, *p* < 0.00001) showed a more prominent statistically positive correlation between ASIR, ASPR with the SEV related to HFPG compare to female (ASIR: R = 0.17, *p* < 0.00001; ASPR: R = 0.18, *p* < 0.00001) (Supplementary Figures 1, 2).

**Figure 5:**
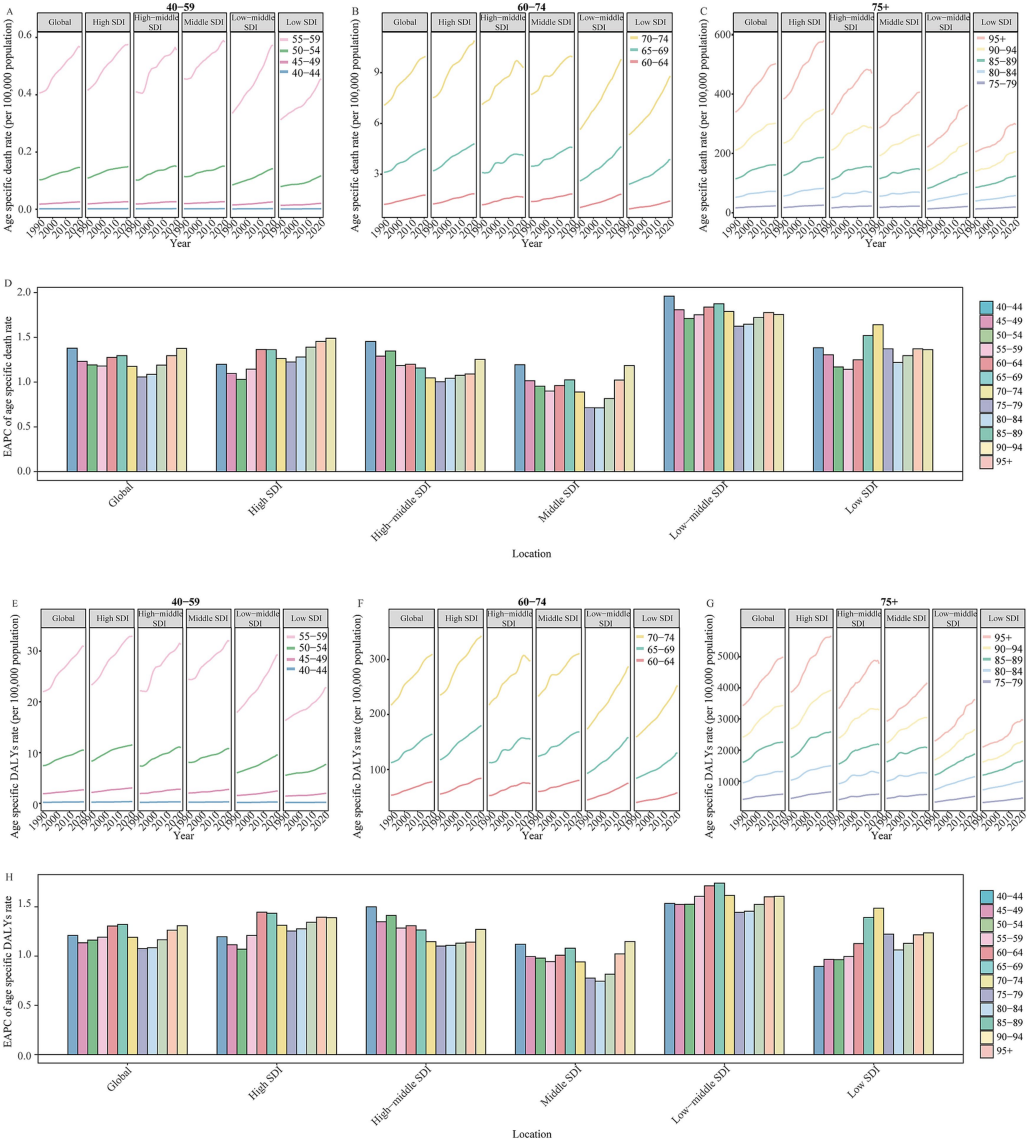
**(A)** The change in high fasting plasma glucose contributed to the DALYs and death rate for Alzheimer’s disease and other dementias by age in global and SDI regions from 1990 to 2021. **(B)** The change in high fasting plasma glucose contributed to age specific death rate of Alzheimer’s disease and other dementias for 40 to 59 age groups in global and different SDI regions from 1990 to 2021. **(C)** The change in high fasting plasma glucose contributed to age specific death rate of Alzheimer’s disease and other dementias for 60 to 74 age groups in global and different SDI regions from 1990 to 2021. The change in high fasting plasma glucose contributed to age specific death rate of Alzheimer’s disease and other dementias for people aged 75 years and over in global and different SDI regions from 1990 to 2021. **(D)** The EAPC of high fasting plasma glucose contributed to age specific death rate for Alzheimer’s disease and other dementias by age in global and different SDI regions from 1990 to 2021. **(E)** The change in high fasting plasma glucose contributed to age specific DALYs rate of Alzheimer’s disease and other dementias for 40 to 59 age groups in global and different SDI regions from 1990 to 2021. **(F)** The change in high fasting plasma glucose contributed to age specific DALYs rate of Alzheimer’s disease and other dementias for 60 to 74 age groups in global and different SDI regions from 1990 to 2021. **(G)** The change in high fasting plasma glucose contributed to age specific DALYs rate of Alzheimer’s disease and other dementias for people aged 75 years and over in global and different SDI regions from 1990 to 2021. **(H)** The EAPC of high fasting plasma glucose contributed to age specific DALYs rate for Alzheimer’s disease and other dementias by age in global and different SDI regions from 1990 to 2021. SDI, socio-demographic index; EAPC, estimated annual percentage change; DALYs, disability-adjusted life-years.

Figure 5 shows the DALYs and death rate of HFPG-attributable ADOD, which also increased with age, peaking in the 95 + age group. All age groups reported an upward trend in death rates globally and across all SDI regions from 1990 to 2021. The population aged 60 years and older accounted for the central part of the total DALYs rate, and uptrends also occurred in global and all SDI regions in the study. Significantly, the 60 to 74 age groups showed a more substantial increase in the DALYs rate compared to the 75 to 84 age group globally, and these populations also experienced a more prominent rise than the 75 to 89 age groups in high to low-middle SDI regions over the past 32 years. Furthermore, further analysis of the statistically significant correlation between ASIR and ASPR with the SEV related to HFPG revealed that age groups 60 to 74 have a more positive correlation than the population aged 80 and above (Table 2; Supplementary Figures 1, 2).

**Table 2:** The correlation between the incidence and prevalence rate due to Alzheimer’s disease and other dementias and summary exposure value related to high fasting plasma glucose for population age 60 and over in 21 GBD regions from 1990 to 2021.

Age	Incidence		Prevalence	
	Pearson R	*p*-value	Pearson R	*p*-value
60–64 years	0.16	**<0.00001**	0.18	**<0.00001**
65–69 years	0.16	**<0.00001**	0.17	**<0.00001**
70–74 years	0.23	**<0.00001**	0.18	**<0.00001**
75–79 years	0.16	**<0.00001**	0.17	**<0.00001**
80–84 years	0.017	0.66	0.063	0.1
85–89 years	−0.0027	0.94	0.024	0.54
90–94 years	0.039	0.31	0.049	0.21
95 + years	**0.096**	0.012	0.13	**0.00062**

GBD, Global Burden of Disease. Bold *p* values indicates statistical significance (*p* < 0.05).

## Discussion

Using GBD 2021 data, this study analyzed the changes in ADOD and HFPG-attributable ADOD burden at the global, regional, country and territory levels. The ADOD burden has continuously increased globally and in most regions. East Asia and China exhibited the highest ADOD burden among regions or countries in 2021, with sharp increases from 1990 to 2021. The ASIR and ASPR positively correlated with the SEV related to HFPG. Trends in HFPG-attributable ADOD burden have been observed globally, in all regions, and nearly all countries and territories over the past 32 years. People aged 60 and over accounted for the majority of ADOD and HFPG-attributable ADOD burden. Notably, the 60 to 74 age groups have prominent increases in incidence and prevalence rate and relatively poor control in DALYs and death rate compared to the people aged 75 and over in global and higher SDI regions. Meanwhile, 60 to 74 age groups also showed significant increases in the HFPG-attributable DALYs rate in global and most SDI regions over the past 32 years. A prominent positive correlation was observed between the incidence and prevalence rates of SEVs related to HFPG in 60 to 74 age groups compared to those aged 80 and over.

Our analysis indicated that ADOD remains a significant health burden globally, with more than 56 million survivors and approximately 2.0 million deaths in 2021. The increasing number is likely due to population growth and aging, advanced diagnostic techniques, and increased public awareness of ADOD (7, 13–15). The Coronavirus Disease 2019 (COVID-19) pandemic significantly impacted the care and disease burden of individuals living with ADOD (16–19). ASRs of ADOD burden have changed less prominently over the past 32 years. Probably, the educational, socioeconomic, health care, and lifestyle changes have contributed significantly to the control of the ADOD disease burden. However, increasing obesity, Diabetes, and declining physical activity might reverse this trajectory (7). Therefore, identifying and targeting modifiable risk factors may be effective strategies to reduce the burden of dementia.

Higher SDI regions exceeded the greater ADOD burden during the period. In 2015, the prevalence of dementia had reached 6.4% in America, which was higher than in Africa (4.6%) (20). Relevant studies have reported that the majority of global dementia care costs and dementia research are focused on high-income countries (HICs) (14). The National Alzheimer’s Project Act in the United States, Living Well With Dementia in the United Kingdom, and the National Dementia Strategy in Germany share common goals: to enhance public awareness, improve diagnosis and care, and provide support for caregivers and families (8). A global healthy life expectancy and population estimation revealed that HICs have a longer life expectancy and a greater proportion of the population aged 65 years and older compared to low-income regions (21). Moreover, several modifiable risk factors for dementia, such as hypertension, Diabetes, overweight, and obesity, are prevalent in HICs (22–25). The European and African regions had a prevalence of obesity of 22.9 and 12.7%, respectively, in 2015 (22). Fortunately, we observed the decline in ASRs of ADOD burden in high SDI region during the study period. A growing number of epidemiological studies revealed stable or decreasing incidence and prevalence of dementia in HICs over the last few decades (26, 27). The decrease in dementia rates has been primarily attributed to improved cardiovascular health resulting from lifestyle changes, enhanced social and welfare systems, prompt diagnosis and treatment of comorbidities, and increasing educational levels (15). Additionally, the effective management of metabolic risk factors (such as hyperglycemia, hyperlipidemia, hypertension, and obesity) may contribute positively to this change. Japan implemented Specific Health Checkups to control unhealth-related risk factors, which include waist circumference, BMI, blood lipids, blood glucose, and blood pressure (28).

Of concern is that lower SDI regions experienced a greater increase in DALYs and death burden in the study period. Several common factors across low-income and middle-income countries (LMICs) that may contribute to this change include low public awareness of ADOD, poor literacy, insufficient knowledge of ADOD-related issues, a lack of interprofessional cooperation, and inadequate access to suitable services. 90% of dementia cases in LMICs go undiagnosed (15). The WHO’s 2021 report showed that most LMICs do not have a national dementia plan (15, 29, 30). Another potential reason may be that the COVID-19 pandemic has significantly imposed immense stress on already strained medical resources and limited access to care across low-and middle-income countries, thereby aggravating the ADOD burden in these region (16, 17, 31, 32). Several modifiable risk factors for ADOD, such as hypertension, dyslipidemia, Diabetes, and obesity, have significantly increased in LMICs. Nearly 80% of global CVD deaths take place in LMICs (33). From 1975 to 2015, the highest worldwide blood pressure values were suggested to have shifted from HICs to LMICs in South Asia and sub-Saharan Africa (15). Metabolic diseases such as type 2 diabetes mellitus (T2DM), obesity, and hypercholesterolemia have consistently increased in lower SDI regions in the last three decades (34, 35). Moreover, population growth and the shift in population structure toward older adults may also contribute to the ADOD burden in LMICs. Therefore, national dementia plans, inclusive research, dementia policies, and interventions are urgently needed to reduce the ADOD burden in LMICs. The WHO 2017–2025 public health dementia plan aims to implement dementia awareness-raising campaigns in 100% of countries worldwide. Due to the scarcity of health and human resources, LMICs should consider mandating a task-shifting approach to integrating dementia care into chronic disease clinics, delivered by non-specialist health workers (36).

East Asia exhibited the highest ADOD burden among 21GBD regions in 2021, with the fastest increase from 1990 to 2021. Population aging is a significant contributor to the ADOD burden in East Asia (15). The Apolipoprotein E (ApoE) ε4 allele, as the major genetic risk factor for dementia, has been shown to have the most significant association with Alzheimer’s disease (the most common type of dementia) in East Asians (3, 15). Global ASDR of ischemic heart disease (IHD) declined, but the rise was found in East Asia in recent years (37). China also experienced a high ADOD burden in 2021, with the most significant increase in ASPR and ASIR across all countries from 1990 to 2021. The relevant study suggested a 46% relative increase in Chinese ASPR of dementia from 1990 to 2010 (+2.3% per year) (26). The COVID-19 pandemic was a potentially adverse contributor to the ADOD burden in China (8, 38). A cross-sectional survey reported the PAF of nine modifiable risk factors (lower early-life education, midlife hearing loss, hypertension, obesity, later-life smoking, depression, physical inactivity, social isolation, and Diabetes) for dementia prevalence was 39.5% in China (39). Ischemic heart disease and stroke have consistently increased in China in the past 30 years (37). In China, deaths due to a hyperglycaemic crisis made up 8–10% of all deaths in individuals with Diabetes, compared with less than 1% in the UK (40). To address the challenges of increasing ADOD burden, China set out the Work Program for Exploring Special Services for Dementia Prevention and Control and determined to execute a nationwide project of dementia prevention and care promotion from 2023 to 2025. Moreover, China’s Action Plan for Healthy China 2030 has issued a feasible scheme to promote healthy aging (8).

The *2020 Lancet Commission on dementia prevention, intervention, and Care* reported that the total potentially modifiable risk factors may prevent or delay around 40% of dementia (7). Our result revealed that HFPG accounted for 15% of all deaths and DALYs due to ADOD worldwide in 2021. A cohort study of 15, 744 black and white adults indicated the late-life dementia risk of midlife exposure to Diabetes [hazard ratio (HR), 1.77; 95% CI: 1.53–2.04] was higher than lower educational attainment (HR, 1.61; 95% CI, 1.28–2.03), midlife smoking (HR, 1.41; 95% CI, 1.23–1.61), prehypertension (HR, 1.31; 95% CI, 1.14–1.51), hypertension (HR, 1.39; 95% CI, 1.22–1.59), overweight (HR,1.05; 95% CI: 0.92–1.19) and hyperlipidemia (not significant difference) (41). A nationwide population-based cohort study from South Korea reported that the varying degrees and durations of hyperglycemia were associated with an increased risk of incident dementia (42). Our study partly corroborates these findings, revealing a positive correlation between the incidence and prevalence of ADOD with SEV for HFPG. Related mechanisms that demonstrate the association between high fasting plasma glucose and ADOD are not fully understood. It may be partially mediated by insulin resistance related to hyperglycemia, which disrupts brain insulin signaling and promotes the accumulation of amyloid-*β* in the brain. Additionally, hyperglycemia causes minor vessel damage in the brain through impaired blood supply, ultimately leading to dementia (42). Moreover, the APOE ε4 allele, a well-established genetic risk factor for dementia (43, 44), may synergistically interact with Diabetes. Diabetes increased the risk of incident dementia by an additional 35% in APOE ε4 carriers (relative risk: 1.35, 95% CI: 1.13–1.63) (45). However, the exact biological and genetic mechanisms underlying the interplay between HFPG and other risk factors for ADOD necessitate further exploration. The population-attributable fraction of ADOD incidence and prevalence driven by HFPG exposure remains to be fully elucidated. Notably, the TMREL of FPG is 4.9–5.3 mmol/L in GBD 2021 (12). Therefore, the population with Diabetes and prediabetes should pay attention to the increased risk of ADOD burden.

Notably, our study observed an uptrend in HFPG-attributable ADOD burden in all global regions and most countries and territories over the past 32 years. High SDI regions experienced the highest level and the most significant increase in HFPG-attributable ADOD burden among SDI regions. The diabetes prevalence was estimated to be higher in HICs (11.1%) than in low-income countries (5.5%) in 2021, and the corresponding increases are expected to be 12.2 and 11.0% between 2021 and 2045, respectively (46). Meanwhile, the most significant increase is expected in Africa and Europe. In contrast, the smallest growth is expected in North America and Caribbean, as well as the Western Pacific (46). Lower SDI regions experienced an increase in HFPG-attributable ADOD burden from 1990 to 2021. In LMIC countries, poor access to life-saving technologies such as insulin and tools for monitoring blood glucose concentration, high costs of medications, insufficient health care systems, low patient education resulting in insufficient diabetes diagnosis and treatment, and many avoidable deaths and acute emergencies in patients (40). In previous studies, almost 50% of people surveyed in India, about 40% in China, and 20% in Latin America were unaware that they had Diabetes (39). The deaths due to the hyperglycaemic crisis made up 8–10% of total Diabetes in Mexico and China, compared with less than 1% in the UK (40). With aging and urbanization being inevitable drivers of the epidemic of Diabetes and dementia, the better treatment paradoxically increasing prevalence through decreased mortality, an urgent need exists for the implementation of effective intervention strategies that focus on stalling the increase in the number of the population developing dementia, Diabetes, and related complications.

In our study, female experienced higher ASRs of ADOD burden and HFPG-attributable burden compared to male globally. However, the HFPG-attributable PAFs exhibited opposite gender differences in the period. There are multiple scenarios maybe affect the gender differences in ASRs of ADOD burden (7, 47): (1) female have consistently had a longer life expectancy; (2) depression is demonstrated to increase the risk of dementia; female have twice the risk of depression compared to male after puberty, and worsens in the menopausal transition. (3) female had fewer opportunities for higher education and occupational attainment during the past century. (4) several sex-specific risk factors (such as Hypertensive pregnancy disorders, menopause, perimenopause, combination estrogen plus progestin therapy in later life) for female also increased the dementia risk. Mehak et al. observed that the global incidence and prevalence of Type 2 diabetes mellitus (T2DM) among male were higher than among female from 1990 to 2019 (48). The Non-Communicable Diseases Risk Factor Collaboration also reported a similar trend in global diabetes prevalence and incidence in 2022 (49). This trend partly reveals the higher exposure of HFPG in male; it may partly explain the gender differences in HFPG-attributable PAFs for our study. Absolute rates of HFPG-attributable burden are calculated based on the related PAF and the absolute rates of DALYs and death for ADOD. The inconsistency between PAFs and absolute rates may be because the female sex, as an unmodifiable factor, contributes to a higher risk than HFPG for ADOD. The sex-related disparities in PAFs and the rate of ADOD burden related to HFPG necessitate a further exploration of the underlying factors. By elucidating the intricate interplay between sex, hormones, brain development, educational attainment, genetics, and metabolic risk factors, future research can inform targeted interventions and policies in metabolic risk factors (particularly HFPG) to address the growing ADOD burden.

Our results indicated that ASRs of ADOD burden increased with age, peaking in the 95 + age group. The older aged 60 and over experienced the main proportion of ADOD burden. Research from the US reported that 5% of people aged 65 to 74, 13.2% of people aged 75 to 84, and 33.4% of people aged 85 or older were living with Alzheimer’s dementia in 2024 (50). Numerous studies have focused on people aged 60 and older as the primary population for management and prevention related to dementia (20, 26, 50). Crucially, while old age is the strongest known risk factor for dementia, dementia is not a natural and inevitable result of aging (5, 20). The following reasons may partly explain this difference: Firstly, the older population experienced a growing number of medical comorbidities, such as hearing and vision impairment, musculoskeletal disorders, cardiovascular disease, and atherosclerosis (5, 20, 51, 52). Secondly, cognitive demands on this population (who are no longer working and have limited household duties) declined, and the prevalence of insufficient physical activity was highest in numerous regions (20, 53). Moreover, a prospective study demonstrated that the association between the APOE polymorphism and cognition was modified by age, with the APOE ε4 allele demonstrating negative effects in older populations compared to young adults (54). Remarkably, our analysis revealed notable disparities in the change of ADOD burden for older adults: the 60 to 74 age groups have a relatively poor control or even a prominent increase in ADOD burden compared to the 75 + age groups in global and higher SDI regions. Tomoyuki et al. reported that, compared with the 1988 cohort, the incidence of dementia increased in the population aged 65 to 84 years in the 2002 cohort; however, such an uptrend was not observed in individuals aged 85 years and above (10). On the one hand, individuals in the 60 to 74 year age groups may experience smaller genetic risk factors and more modifiable risk factors than those aged 75 years and above. Rasmussen et al. reported that the absolute 10-year risk of Alzheimer’s disease in carriers of the APOE ɛ44 genotype increased with age (43). Therefore, 60 to 74 age groups may be confronted with smaller genetic risk factors compared to those aged 75 years and above. In contrast, other modifiable risk factors (such as metabolic risk factors and smoking) may play important roles in age disparities in the change of ADOD burden. A population-based prospective study reported metabolic syndrome was associated with an increased risk of dementia (hazard ratio:1.11, 95% CI, 1.01–1.21); the association was similar for participants in the 60 to 69 age group (1.21, 1.05–1.39), but attenuated for participants aged 70 to 79 years (0.96, 0.81–1.14). A linear trend was observed between the number of metabolic syndrome components and dementia risk in participants aged 60 to 69 years (P trend = 0.0040) but not in the 70 to 79 age group (55). These findings may partly be consistent with our observation of a prominent positive correlation between the ADOD incidence and prevalence of SEV related to HFPG occurring in the 60 to 74 age groups compared to the 80 + age groups. Moreover, Zhang et al. reported that smoking increased the risk of AD, which was prominent among non-APOE ε4 carriers (56). On the other hand, exposure to metabolic risk factors have been relatively poorly controlled among the 60 to 74 age groups compared with the population aged 75 and over. Relevant epidemiological studies have observed that the global age-specific incidence rate of type 2 diabetes mellitus (T2DM) increases until the age of 70 to 74 years before declining thereafter (48). These age disparities in T2DM may partly account for the significant increase in HFPG-attributable DALYs rate for 60 to 74 age groups in global and most SDI regions over the past 32 years. Similarly, the prevalence of stroke (in China) and metabolic syndrome (among Chinese and three American racial groups) demonstrates an age-dependent pattern, peaking at 70–79 years and 70 years of age, respectively, followed by a gradual decline (57, 58). Furthermore, the age distribution of hospital admissions for ischaemic stroke and subarachnoid hemorrhage in China rises with advancing age, reaching peak incidence at 65–74 years and 70 years, respectively, then decreases with age (59). Notably, while stroke incidence and prevalence increased among individuals younger than 70 years between 1990 and 2021, incidence rates declined substantially in those aged 70 years or older. However, prevalence in this older group remained stable during this period (57). Moreover, Rebecca et al. reported that midlife exposure to obesity, smoking, Diabetes, hypertension, and hypercholesterolemia may increase the risk of 25-year incident dementia (41). Hu et al. showed that the cumulative incidence of dementia was highest among those who were diagnosed with Diabetes at younger than 60 years of age (HR 2.92 [95% CI 2.06, 4.14]). The HRs were 1.73 (95% CI 1.47, 2.04) for onset at 60 to 69 years, 1.23 (95% CI 1.08, 1.40) at 70 to 79 years, 1.13 (95% CI 0.94, 1.35) at aged 80 years or older (60). Another study showed that Diabetes in midlife was associated with a 19% greater cognitive decline over 20 years compared with no diabetes (61). Therefore, effective control of the potential risk factors for different populations should be a focus of future analyses. Further studies are needed to confirm this disparity and better understand the underlying risk factors or biological differences that contribute to it.

### Limitations

Several limitations were present in our study. First, The lack of data from some regions and the negligence of distinctions between urban and rural areas could increase biases in the analyses. Second, despite the availability of improved diagnostic tools for ADOD, the undiagnosed cases of ADOD in the population would lead us to underestimate the ADOD burden. Third, GBD 2021 does not distinguish the subtypes of dementia, for example, vascular dementia, dementia with Lewy bodies, mixed dementia, and Alzheimer’s disease; each of them is associated with differing burdens and risk profiles. Moreover, other risk factors such as genetic predisposition, racial factors, and other modifiable risk factors should be considered in risk profiles for dementia subtypes. Moreover, despite PAFs presenting a comprehensive analysis of the combined impact of multiple risk factors, there may still be potential challenges in precisely analyzing the joint influences of multiple risk factors. Finally, We only obtained the ADOD DALYs and death attributable to the three risk factors, and other risk factors of ADOD burden cannot be downward. Unfortunately, we were unable to obtain or calculate the ADOD incidence, prevalence, and the corresponding population-attributable fraction due to the exposure of risk factors. Therefore, we exclusively analyzed the correlation between the incidence and prevalence rate with the SEVs related to HFPG in this study.

## Conclusion

The substantial burden of dementia has still continuously increased worldwide during the past 32 years. Notably, the 60 to 74 age groups suffered a relatively poor control or even more prominent rise in ADOD burden compared to people aged 75 and over in global and higher SDI regions. The global HFPG-attributable ADOD burden remains prominent and is expected to increase during the study period. Meanwhile, the 60 to 74 age groups suffered a notable rise in HFPG attributable to the DALYs rate of ADOD in global and most SDI regions over time. Moreover, a prominent positive correlation was observed between the incidence and prevalence with the SEVs related to HFPG occurred in the 60 to 74 age groups compared to people aged 80 and over. Therefore, HFPG should be emphasized in strategic priorities for controlling the ADOD burden.

## Data Availability

The datasets presented in this study can be found in online repositories. The names of the repository/repositories and accession number(s) can be found in the article/Supplementary material.

## Footnotes

^1^ https://ghdx.healthdata.org/
