# Supplementary material for: The rising burden of Alzheimer’s and other dementias: role of high fasting plasma glucose from 1990 to 2021
Source: Front Med (Lausanne). 2025 Jun 26;12:1592620. doi: 10.3389/fmed.2025.1592620 (PMC12241058; doi:10.3389/fmed.2025.1592620)
Supplement: Supplementary file 1 [file Data_Sheet_1.pdf]

Supplementary Material

Title: The Rising Burden of Alzheimer’s and Other Dementias: Role of High Fasting Plasma Glucose from 1990 to 2021

Supplementary Figures

Supplementary Figure 1. The correlations between prevalence rate and SEV related to high fasting plasma glucose by sex and age in 21 GBD regions from 1990 to 2021
Supplementary Figure 2. The correlations between incidence rate and SEV related to high fasting plasma glucose by sex and age in 21 GBD regions from 1990 to 2021

Supplementary Tables

Supplementary Table 1. The change of alzheimer's disease and other dementias burden in global and different regions from 1990 to 2021
Supplementary Table 2. The change for alzheimer's disease and other dementias burden at 204 countries and territories from 1990 to 2021
Supplementary Table 3. The alzheimer's disease and other dementias burden by sex in global and different SDI regions from 1990 to 2021
Supplementary Table 4. The change of three risk factors contributed to alzheimer's disease and other dementias burden by sex in global from 1990 to 2021
Supplementary Table 5. The change of high fasting plasma glucose contributed to alzheimer's disease and other dementias burden by sex in different regions from 1990 to 2021
Supplementary Table 6. The change of high fasting plasma glucose contributed to alzheimer's disease and other dementias burden at 204 countries and territories from 1990 to 2021

1. Supplementary Figures

Supplementary Figure 1. The correlations between prevalence rate and SEV related to high fasting plasma glucose by sex and age in 21 GBD regions from 1990 to 2021

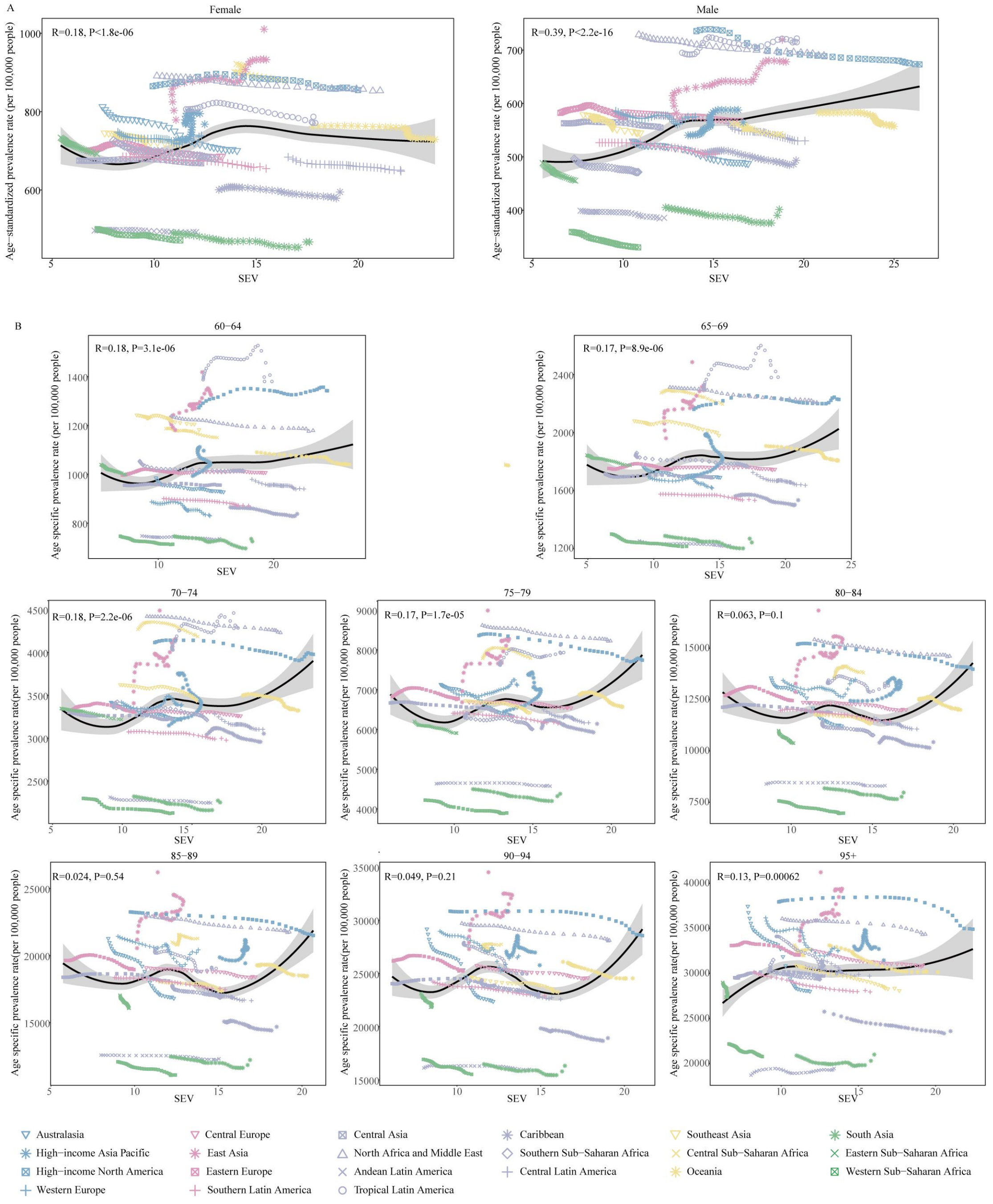

A. The correlations between age-standardized prevalence rate and SEV related to high fasting plasma glucose by sex in 21 GBD regions from 1990 to 2021
B. The correlations between prevalence rate and SEV related to high fasting plasma glucose by age in 21 GBD regions from 1990 to 2021

GBD=Global Burden of Disease; SEV= summary exposure value;

Supplementary Figure 2. The correlations between incidence rate and SEV related to high fasting plasma glucose by sex and age in 21 GBD regions from 1990 to 2021

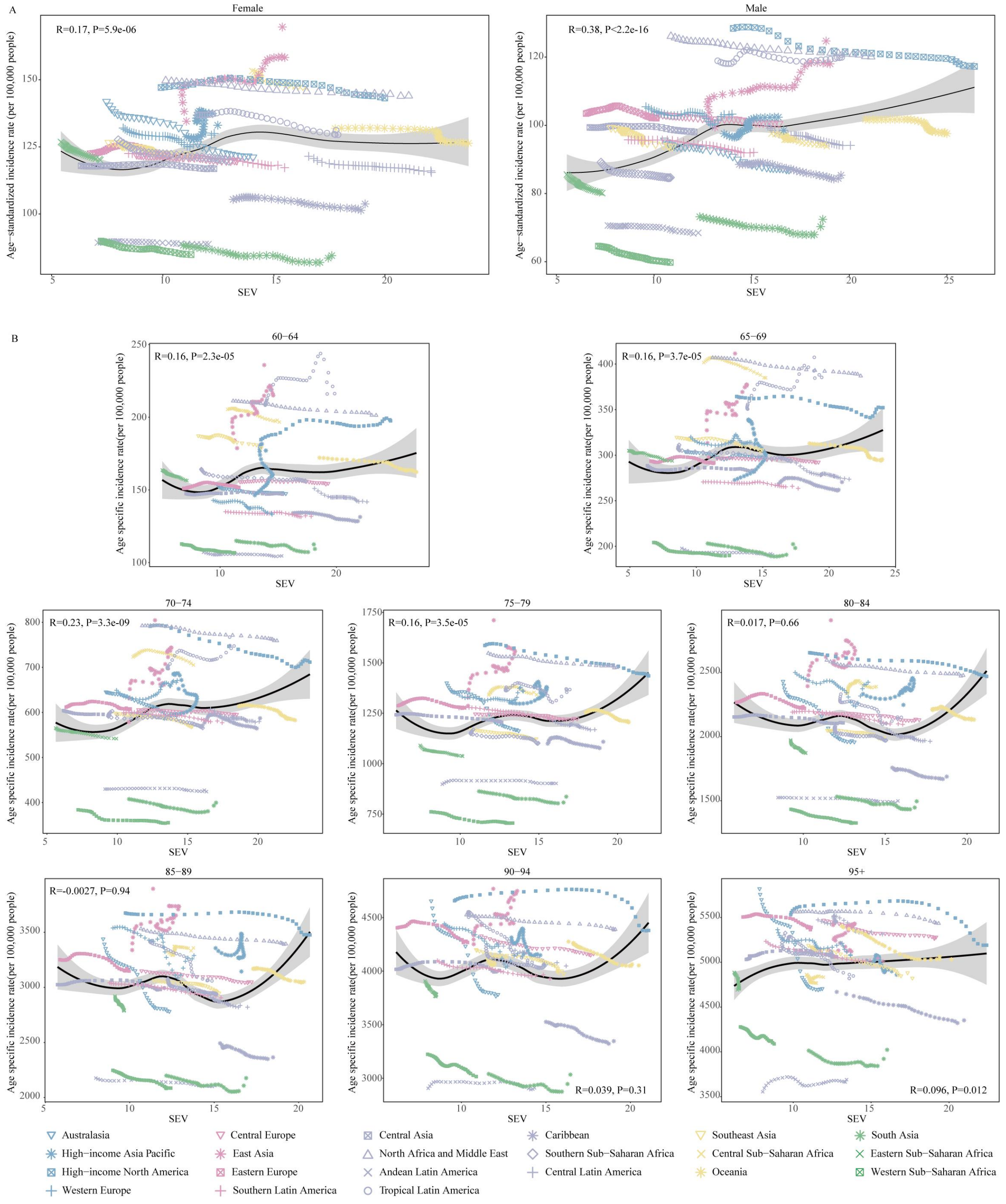

A. The correlations between age-standardized incidence rate and SEV related to high fasting plasma glucose by sex in 21 GBD regions from 1990 to 2021

B. The correlations between incidence rate and SEV related to high fasting plasma glucose by age in 21 GBD regions from 1990 to 2021

GBD=Global Burden of Disease; SEV= summary exposure value;

Supplementary Tables

Supplementary table 1. The change of alzheimer's disease and other dementias burden in global and different regions from 1990 to 2021

Location	Number×10^3 (95% UI)		EAPC of number (95% CI)	ASR (per 100,000 population) (95% UI)		EAPC of ASR (95% CI)	Number×10^3 (95% UI)		EAPC of number (95% CI)	ASR (per 100,000 population) (95% UI)		EAPC of ASR (95% CI)
	1990	2021		1990	2021		1990	2021		1990	2021	
	Prevalence						Incidence					
Global	21799.76(19067.09,24837.69)	56856.69(49382.06,64977.51)	3.10(3.05，3.14）	672.22（588.73，763.95）	694.01（602.88，794.08）	0.00（-0.02，0.03）	3834.53(3367.54,4358.43)	9837.06(8620.52,11163.70)	3.04(3.00，3.09）	116.97（102.77，132.32）	119.76（104.96，135.89）	-0.02（-0.04，0.00）
High SDI	8093.99(7092.81,9188.86)	17216.18(15033.00,19545.52)	2.54(2.51，2.57）	724.21（637.60，815.94）	709.47（619.94，807.40）	-0.05（-0.06，-0.04）	1435.43(1264.75,1624.79)	2952.15(2586.72,3344.55)	2.41(2.39，2.44）	127.18（112.55，142.92）	122.61（107.45，138.44）	-0.11（-0.12，-0.10）
High-middle SDI	5680.57(4933.84,6518.29)	14925.10(12861.05,17155.67)	3.07(3.01，3.14）	685.28（598.24，781.65）	766.20（659.80，879.64）	0.21（0.17，0.25）	990.61(862.20,1138.43)	2582.35(2252.03,2941.77)	3.05(3.00，3.11）	118.38（103.82，134.11）	132.40（115.43，150.85）	0.22（0.18，0.26）
Middle SDI	4831.62(4192.76,5504.06)	16802.25(14485.31,19311.41)	3.93(3.86，4.01）	651.47（566.76，743.90）	723.42（623.26，830.91）	0.12（0.08，0.17）	844.51(735.19,963.60)	2902.08(2542.32,3314.77)	3.90(3.83，3.97）	113.27（99.18，128.90）	123.79（108.25，141.26）	0.09（0.05，0.14）
Low-middle SDI	2350.23(2033.03,2676.29)	6009.38(5214.01,6839.57)	3.08(3.05，3.11）	542.48（473.62，616.16）	524.47（455.82，596.80）	-0.17（-0.19，-0.15）	415.38(363.22,471.14)	1063.28(929.61,1207.79)	3.09(3.05，3.12）	95.55（83.27，108.90）	92.61（80.79，105.71）	-0.17（-0.18，-0.15）
Low SDI	818.15(700.40,930.00)	1854.99(1603.96,2101.15)	2.69(2.64，2.74）	539.74（471.27，612.06）	514.37（447.22，584.18）	-0.19（-0.21，-0.18）	144.16(125.72,163.57)	328.70(287.11,372.98)	2.71(2.66，2.76）	95.08（82.74，108.32）	90.89（79.00，103.12）	-0.19（-0.20，-0.17）
Andean Latin America	78.84(68.47,89.84)	248.16(215.29,283.42)	4.03(3.96,4.10)	450.85（390.47，514.20）	444.09（384.10，507.73）	-0.06（-0.07，-0.05）	14.09(12.25,15.98)	44.49(38.61,50.84)	4.03(3.96,4.10)	80.60（69.91，92.12）	79.55（68.95，91.05）	-0.06（-0.07，-0.05）
Australasia	160.27(139.13,181.94)	363.51(317.55,409.19)	2.56(2.48,2.64)	706.96（614.59，802.27）	604.41（526.77，679.94）	-0.54（-0.58，-0.50）	28.41(24.87,32.32)	63.29(55.46,71.35)	2.48(2.40,2.56)	123.79（108.48，139.61）	105.44（92.56，118.63）	-0.56（-0.60，-0.52）

Caribbean	130.47(113.21,148.47)	301.09(261.14,341.20)	2.61(2.55,2.67)	557.24（485.64，629.27）	550.22（476.89，624.84）	-0.15（-0.18，-0.12）	22.91(19.92,26.13)	52.40(45.97,59.45)	2.60(2.54,2.66)	97.60（85.15，110.90）	95.60（83.53，108.64）	-0.16（-0.19，-0.13）
Central Asia	256.02(221.62,290.36)	398.79(345.21,451.92)	1.53(1.39,1.67)	638.07（553.08，725.58）	626.81（541.85，713.52）	-0.06（-0.08，-0.04）	44.78(39.05,51.31)	69.75(61.21,79.09)	1.55(1.43,1.67)	111.72（97.73，127.69）	109.82（95.87，125.30）	-0.06（-0.07，-0.04）
Central Europe	868.66(747.73,997.94)	1540.94(1327.85,1766.51)	2.07(2.00,2.15)	657.16（565.40，750.94）	641.22（554.05，732.31）	-0.09（-0.09，-0.08）	152.64(131.92,177.17)	269.69(233.10,309.82)	2.05(1.98,2.12)	115.01（100.69，131.62）	112.42（97.97，128.46）	-0.08（-0.09，-0.07）
Central Latin America	420.13(364.26,478.95)	1397.10(1216.19,1585.19)	4.13(4.08,4.19)	625.86（543.81，713.04）	596.48（518.98，680.63）	-0.11（-0.12，-0.09）	74.98(65.39,85.45)	248.66(218.26,282.09)	4.12(4.07,4.18)	111.25（96.71，126.56）	106.12（92.53，120.99）	-0.11（-0.12，-0.09）
Central Sub-Saharan Africa	100.18(85.94,114.16)	253.63(220.06,287.41)	2.99(2.95,3.03)	752.86（650.12，852.75）	750.54（654.70，848.11）	0.00（-0.02，0.03）	17.48(15.15,19.94)	44.06(38.63,49.58)	2.97(2.93,3.00)	126.90（111.06，144.40）	126.14（111.31，143.21）	-0.01（-0.04，0.01）
East Asia	4151.55(3556.09,4768.97)	17414.17(14854.02,20142.13)	4.38(4.26,4.50)	697.26（603.50，802.56）	887.95（759.95，1027.48）	0.43（0.35，0.51）	725.92(621.29,834.63)	2988.72(2569.17,3434.39)	4.33(4.22,4.44)	120.29（104.75，137.02）	149.61（129.58，171.14）	0.40（0.33，0.48）
Eastern Europe	1653.42(1425.00,1903.78)	2380.52(2059.57,2730.95)	1.28(1.22,1.35)	669.20（578.77，768.96）	658.68（571.31，752.67）	-0.08（-0.12，-0.04）	291.03(251.71,335.61)	416.09(361.58,476.87)	1.27(1.21,1.32)	117.31（102.49，133.78）	115.66（100.94，131.87）	-0.08（-0.11，-0.04）
Eastern Sub-Saharan Africa	306.61(262.59,348.49)	692.23(597.29,780.54)	2.72(2.62,2.82)	618.54（538.17，701.19）	588.72（513.88，667.20）	-0.14（-0.15，-0.14）	53.37(46.29,60.69)	121.21(106.42,137.29)	2.74(2.64,2.84)	107.12（93.44，121.63）	102.41（89.70，116.09）	-0.13（-0.14，-0.12）
High-income Asia Pacific	1182.07(1025.97,1345.12)	4109.52(3547.34,4692.56)	4.36(4.22,4.51)	658.06（572.10，745.38）	684.82（596.97，780.11）	0.28（0.23，0.34）	212.52(185.44,242.78)	701.76(614.64,802.67)	4.18(4.03,4.33)	116.94（102.53，133.08）	118.62（103.43，135.00）	0.19（0.14，0.24）
High-income North America	3021.80(2617.28,3444.67)	5508.42(4800.05,6295.24)	1.88(1.85,1.92)	815.90（709.63，929.16）	775.11（673.67，885.78）	-0.21（-0.23，-0.19）	520.20(454.54,593.02)	928.26(812.57,1051.34)	1.81(1.77,1.84)	139.57（122.14，157.89）	131.39（114.67，149.04）	-0.22（-0.24，-0.20）
North Africa and Middle East	977.03(843.77,1104.04)	2684.37(2322.37,3040.39)	3.44(3.41,3.47)	812.51（708.72，918.84）	772.66（671.20，877.55）	-0.17（-0.17，-0.16）	170.21(149.71,192.33)	468.05(411.55,530.96)	3.43(3.41,3.46)	138.06（121.37，156.98）	132.19（115.75，150.35）	-0.14（-0.15，-0.13）

Oceania	11.66(9.92,13.44)	30.70(26.25,34.93)	3.10(3.05,3.14)	676.80（584.15，778.03）	644.83（554.28，737.37）	-0.20（-0.22，-0.18）	2.06(1.76,2.37)	5.42(4.71,6.17)	3.09(3.04,3.14)	117.17（102.35，133.78）	112.05（96.98，128.81）	-0.18（-0.21，-0.16）
South Asia	1759.04(1517.06,2008.72)	5147.48(4444.26,5886.07)	3.53(3.46,3.59)	446.47（386.42，510.25）	437.07（377.00，500.95）	-0.18（-0.22，-0.14）	315.18(273.89,359.43)	924.83(799.98,1056.26)	3.54(3.47,3.61)	80.57（69.50，92.11）	79.00（68.26，90.52）	-0.18（-0.22，-0.14）
Southeast Asia	1269.36(1094.13,1445.54)	3387.80(2922.77,3857.74)	3.21(3.19,3.23)	675.02（589.62，770.17）	644.38（560.58，737.69）	-0.14（-0.16，-0.12）	216.53(189.08,245.89)	578.24(505.93,658.51)	3.22(3.20,3.23)	114.85（100.81，130.85）	110.07（96.11，125.72）	-0.14（-0.15，-0.12）
Southern Latin America	259.76(223.39,295.89)	546.19(471.61,621.96)	2.41(2.34,2.48)	622.39（538.28，706.11）	595.26（514.48，676.41）	-0.15（-0.16，-0.14）	46.98(40.64,53.79)	98.31(85.03,112.62)	2.39(2.32,2.46)	111.77（97.43，127.36）	107.09（92.90，122.43）	-0.14（-0.15，-0.13）
Southern Sub-Saharan Africa	137.24(118.25,156.39)	268.80(231.16,306.09)	2.14(2.09,2.19)	639.88（554.13，730.43）	606.69（524.69，692.07）	-0.14（-0.16，-0.13）	24.23(21.09,27.41)	47.44(41.32,54.03)	2.13(2.08,2.18)	112.76（98.34，128.64）	107.13（93.16，122.19）	-0.14（-0.15，-0.12）
Tropical Latin America	540.71(467.91,618.34)	1862.09(1617.47,2116.52)	4.06(3.98,4.14)	758.68（660.76，863.19）	759.83（660.00，867.54）	-0.05（-0.10，-0.01）	92.95(81.38,105.41)	311.56(274.80,352.66)	3.97(3.90,4.05)	129.14（113.10，146.66）	126.83（111.77，144.42）	-0.11（-0.15，-0.08）
Western Europe	4233.26(3694.05,4798.87)	7747.19(6705.23,8805.14)	2.02(1.97,2.07)	691.03（608.15，774.18）	670.36（583.83，762.62）	-0.11（-0.14，-0.08）	758.29(671.20,848.02)	1352.81(1182.31,1543.87)	1.92(1.87,1.98)	122.45（108.70，136.67）	118.56（103.36，134.23）	-0.14（-0.18，-0.11）
Western Sub-Saharan Africa	281.67(241.90,319.13)	573.97(493.78,650.72)	2.32(2.25,2.40)	436.85（379.40，496.47）	406.02（352.89，462.39）	-0.24（-0.26，-0.22）	49.77(43.26,56.44)	102.00(88.93,115.14)	2.35(2.28,2.43)	78.42（67.93，89.46）	73.18（63.36，83.46）	-0.23（-0.24，-0.21）
**Location**	**DALYs**						**Death**					
Global	13572.31(6439.34,29586.87)	36332.69(17237.62,76873.28)	3.22(3.16，3.27）	445.75（206.08，958.03）	450.98（212.69，950.16）	-0.02（-0.03，0.00）	663.29(163.58,1764.99)	1952.68(512.98,4984.74)	3.56(3.50，3.62）	25.04（6.29，66.28）	25.16（6.68，64.25）	-0.02（-0.03，-0.01）
High SDI	5178.32(2446.91,11074.20)	11731.99(5566.77,24033.20)	2.71(2.67，2.75）	475.13（223.26，1007.56）	460.75（220.37，948.43）	-0.11（-0.12，-0.10）	281.91(71.31,738.79)	719.33(196.68,1762.39)	3.09(3.05，3.13）	27.06（6.97，70.01）	26.21（7.05，64.83）	-0.12（-0.13，-0.11）
High-middle SDI	3550.38(1664.39,7789.66)	9243.77(4399.64,19638.39)	3.10(3.04，3.17）	460.79（211.15，995.92）	481.70（228.79，1023.96）	0.07（0.04，0.09）	170.71(41.72,462.49)	490.09(128.59,1265.12)	3.47(3.38，3.55）	25.91（6.38，69.59）	26.42（7.01，68.31）	0.02（-0.01，0.04）

Middle SDI	2975.64(1398.26,6593.08)	10141.16(4901.24,21854.76)	3.95(3.87，4.04）	434.43（200.09，947.97）	455.35（215.96，982.76）	0.03（0.00，0.05）	130.85(31.33,349.33)	493.42(123.09,1282.12)	4.34(4.25，4.43）	23.69（5.75，63.90）	24.56（6.30，64.53）	0.03（0.00，0.05）
Low-middle SDI	1357.81(641.76,3022.10)	3925.27(1774.10,8561.48)	3.53(3.50，3.56）	334.98（157.27，731.97）	360.40（164.09，783.93）	0.23（0.22，0.24）	58.42(13.86,159.85)	189.59(45.90,510.23)	3.96(3.93，3.99）	17.72（4.27，49.17）	20.00（4.85，54.05）	0.43（0.41，0.45）
Low SDI	495.71(228.39,1102.22)	1260.61(565.04,2856.16)	3.14(3.06，3.21）	358.32（163.29，788.84）	383.04（167.71，863.74）	0.24（0.18，0.29）	20.73(4.97,57.26)	58.64(13.91,162.64)	3.56(3.49，3.63）	19.56（4.72，54.84）	22.07（5.30，61.34）	0.45（0.36，0.53）
Andean Latin America	47.31(22.35,102.11)	151.02(72.68,318.35)	4.08(4.01,4.14)	278.53（129.89，603.92）	272.02（131.15，573.64）	-0.11（-0.13，-0.09）	2.30(0.55,6.18)	7.68(1.91,20.68)	4.32(4.22,4.42)	14.54（3.51，39.06）	14.08（3.49，37.90）	-0.13（-0.15，-0.11）
Australasia	97.15(47.03,204.50)	252.18(117.59,522.76)	3.06(2.99,3.12)	443.49（212.56，928.52）	405.09（190.47，836.34）	-0.30（-0.32，-0.29）	5.00(1.23,13.39)	15.25(4.01,38.57)	3.63(3.53,3.73)	24.52（6.14，64.68）	23.18（6.03，58.68）	-0.17（-0.19，-0.15）
Caribbean	71.56(35.51,153.77)	174.18(85.23,368.49)	2.84(2.77,2.91)	321.56（155.29，684.78）	313.56（153.61，661.20）	-0.12（-0.14，-0.11）	3.24(0.78,9.11)	9.07(2.25,24.63)	3.31(3.18,3.43)	16.35（3.92，44.84）	15.82（3.87，43.17）	-0.10（-0.11，-0.09）
Central Asia	149.21(71.67,319.46)	232.27(112.59,498.32)	1.41(1.25,1.56)	388.52（183.29，831.85）	379.25（181.20，819.07）	-0.10（-0.11，-0.09）	7.26(1.79,19.90)	11.02(2.73,30.44)	1.27(1.09,1.46)	20.66（5.07，56.11）	20.07（5.03，55.69）	-0.12（-0.13，-0.11）
Central Europe	488.56(236.36,1078.06)	933.20(446.02,1970.59)	2.29(2.20,2.39)	391.81（185.38，853.38）	386.04（184.19，812.17）	-0.05（-0.06，-0.04）	22.49(5.46,63.37)	49.87(12.52,133.19)	2.80(2.65,2.95)	20.75（5.08，56.95）	20.46（5.15，54.29）	-0.04（-0.05，-0.03）
Central Latin America	220.40(109.63,471.32)	783.86(384.33,1643.52)	4.32(4.26,4.37)	343.12（166.95，727.80）	335.60（163.80，703.51）	-0.07（-0.07，-0.06）	9.71(2.38,26.79)	38.74(9.58,102.46)	4.71(4.63,4.79)	17.16（4.23，46.44）	16.84（4.16，44.59）	-0.07（-0.08，-0.06）
Central Sub-Saharan Africa	62.70(28.37,141.97)	178.40(78.70,408.10)	3.44(3.39,3.48)	535.40（238.50，1185.21）	591.41（255.67，1360.26）	0.36（0.33，0.40）	2.51(0.60,6.84)	8.11(1.93,22.19)	3.96(3.93,4.00)	30.39（7.51，79.92）	34.89（8.43，93.23）	0.51（0.45，0.57）
East Asia	2781.64(1277.19,6270.51)	10359.13(5080.42,22833.68)	4.14(4.02,4.26)	527.37（233.39，1172.37）	555.11（267.58，1222.86）	-0.02（-0.07，0.02）	123.42(29.18,331.21)	507.66(129.18,1368.54)	4.54(4.40,4.67)	30.87（7.45，82.33）	30.41（7.81，81.29）	-0.19（-0.23，-0.14）

Eastern Europe	934.16(452.80,2040.93)	1438.73(673.86,3104.95)	1.45(1.32,1.58)	403.75（191.34，870.95）	396.72（186.44，853.77）	-0.07（-0.09，-0.05）	43.18(10.54,122.19)	75.31(18.26,209.06)	1.85(1.60,2.11)	21.50（5.22，59.64）	20.98（5.07，57.85）	-0.09（-0.10，-0.08）
Eastern Sub-Saharan Africa	191.45(89.28,426.49)	488.06(218.47,1080.94)	3.14(3.04,3.25)	430.46（196.38，949.41）	460.68（200.73，1028.27）	0.24（0.23，0.25）	8.13(1.97,22.48)	23.35(5.59,64.42)	3.56(3.47,3.66)	24.15（5.95，66.84）	27.18（6.72，73.25）	0.41（0.40，0.42）
High-income Asia Pacific	795.71(373.98,1726.61)	3028.73(1439.61,6015.52)	4.56(4.46,4.67)	470.24（218.13，1006.65）	461.33（222.39，928.28）	0.01（-0.01，0.04）	42.16(10.71,109.37)	200.16(57.94,463.17)	5.26(5.15,5.36)	27.79（7.27，71.04）	26.59（7.46，62.96）	-0.10（-0.13，-0.06）
High-income North America	1931.57(915.98,4111.23)	3664.55(1728.30,7650.51)	2.04(2.00,2.07)	522.13（247.40，1104.02）	499.20（236.41，1041.63）	-0.18（-0.20，-0.16）	106.65(27.64,276.05)	216.71(58.26,541.29)	2.29(2.25,2.33)	29.01（7.56，74.58）	28.25（7.49，71.28）	-0.11（-0.13，-0.10）
North Africa and Middle East	578.98(280.43,1264.23)	1566.07(753.72,3314.93)	3.41(3.36,3.45)	515.80（245.62，1112.57）	476.29（225.56，1004.20）	-0.27（-0.30，-0.25）	26.39(6.40,70.58)	73.79(18.12,190.47)	3.64(3.56,3.72)	28.02（6.81，75.27）	25.61（6.31，66.80）	-0.30（-0.35，-0.26）
Oceania	6.68(3.20,14.22)	17.63(8.41,38.11)	3.14(3.11,3.17)	427.58（201.47，930.31）	397.82（184.77，863.92）	-0.28（-0.30，-0.26）	0.26(0.06,0.73)	0.73(0.17,2.04)	3.49(3.44,3.55)	22.93（5.60，63.25）	21.08（5.19，59.87）	-0.32（-0.35，-0.30）
South Asia	1009.43(467.21,2351.86)	3450.14(1531.59,7711.44)	4.13(4.08,4.19)	272.02（126.23，615.50）	308.27（135.52，684.96）	0.40（0.37，0.43）	41.79(9.60,119.80)	165.38(39.36,454.12)	4.68(4.62,4.74)	14.11（3.23，40.84）	17.20（4.10，47.28）	0.70（0.66，0.75）
Southeast Asia	703.42(344.01,1485.73)	2086.18(973.51,4362.20)	3.51(3.48,3.54)	400.20（192.50，839.35）	418.63（193.30，883.66）	0.12（0.08，0.15）	30.07(7.13,80.14)	98.37(24.21,259.33)	3.81(3.76,3.85)	20.67（4.97，55.97）	22.64（5.56，59.23）	0.24（0.20，0.29）
Southern Latin America	150.66(73.81,321.85)	340.21(162.52,709.45)	2.67(2.57,2.76)	379.33（183.13，803.78）	368.23（176.23，766.73）	-0.08（-0.10，-0.07）	7.40(1.84,20.36)	18.79(4.81,48.96)	3.08(2.94,3.23)	20.61（5.15，55.38）	20.06（5.12，52.25）	-0.06（-0.07，-0.04）
Southern Sub-Saharan Africa	81.98(39.19,177.35)	168.43(77.83,376.44)	2.26(2.18,2.34)	401.95（189.45，867.33）	408.97（187.40，904.57）	0.05（-0.01，0.10）	3.88(0.95,10.57)	7.86(1.89,21.83)	2.22(2.13,2.31)	21.61（5.33，58.95）	22.70（5.42，62.82）	0.14（0.06，0.22）
Tropical Latin America	341.58(159.91,755.00)	1226.23(576.49,2613.82)	4.26(4.20,4.32)	510.43（234.39，1109.41）	503.17（235.73，1070.77）	-0.05（-0.07，-0.04）	15.92(3.94,41.76)	65.23(17.12,164.36)	4.77(4.67,4.87)	28.03（7.29，73.09）	27.25（7.13，68.87）	-0.06（-0.08，-0.05）

Western Europe	2746.24(1292.22,5841.69)	5385.86(2539.41,11019.00)	2.25(2.22,2.29)	460.18（215.66，971.82）	443.24（211.93，909.88）	-0.12（-0.13，-0.11）	153.04(38.47,403.81)	339.57(90.93,836.29)	2.64(2.60,2.68)	26.77（6.83，69.36）	25.78（6.82，64.03）	-0.11（-0.12，-0.09）
Western Sub-Saharan Africa	181.91(82.65,402.09)	407.63(181.17,942.45)	2.70(2.61,2.79)	311.57（138.94，686.95）	320.74（138.35，745.09）	0.14（0.10，0.18）	8.52(2.07,23.46)	20.04(4.71,56.35)	2.94(2.86,3.02)	18.02（4.36，49.87）	19.25（4.66，53.64）	0.29（0.24，0.33）

ASR: Age-standardized rate; GBD: Global Burden of Diseases; DALYs: disability-adjusted life-years; UI: uncertainty intervals; CI: confidence intervals; EAPC: estimated annual percentage change; SDI: Socio-Demographic Index;

Supplementary table 2. The change for alzheimer's disease and other dementias burden at 204 countries and territories from 1990 to 2021

Location	ASPR (per 100,000 population) (95% UI)		EAPC of ASPR (95% CI)	ASIR (per 100,000 population) (95% UI)		EAPC of ASIR (95% CI)	ASYR (per 100,000 population) (95% UI)		EAPC of ASYR (95% CI)	ASDR (per 100,000 population) (95% UI)		EAPC of ASDR (95% CI)
	1990	2021		1990	2021		1990	2021		1990	2021	
Afghanistan	777.91（674.21，886.04）	761.83（655.07，867.52）	-0.07(-0.09，-0.06）	132.83（116.86，152.07）	130.94（115.12，149.09）	-0.05(-0.06，-0.03）	604.48（259.91，1383.43）	577.72（251.23，1271.17）	-0.16(-0.18，-0.14）	35.31（8.65，95.86）	33.27（8.44，89.55）	-0.20(-0.23，-0.17）
Albania	646.38（556.60，736.69）	648.77（562.35，743.87）	0.03(0.02，0.04）	111.95（97.18，127.90）	112.62（98.00，128.70）	0.04(0.03，0.05）	402.99（189.66，887.25）	391.82（185.72，836.06）	-0.08(-0.10，-0.06）	21.63（5.36，59.16）	20.82（4.95，56.60）	-0.12(-0.14，-0.09）
Algeria	787.05（675.35，896.79）	752.64（650.93，866.12）	-0.16(-0.17，-0.15）	134.08（117.34，152.85）	128.59（112.65，146.91）	-0.14(-0.15，-0.14）	510.58（240.11，1138.87）	479.05（224.05，1029.30）	-0.19(-0.21，-0.17）	28.02（6.74，76.98）	26.19（6.43，68.19）	-0.17(-0.20，-0.14）
American Samoa	640.58（540.81，740.34）	626.63（528.15，722.51）	-0.09(-0.10，-0.08）	111.24（95.84，128.11）	109.14（93.39，125.47）	-0.08(-0.09，-0.07）	434.66（193.98，981.06）	405.32（188.48，890.49）	-0.19(-0.22，-0.15）	24.16（5.81，66.64）	22.47（5.43，60.53）	-0.17(-0.22，-0.12）
Andorra	688.92（594.24，791.59）	645.09（551.71，741.30）	-0.21(-0.21，-0.20）	121.55（105.04，139.09）	114.28（98.41，131.55）	-0.19(-0.20，-0.19）	449.15（213.89，925.89）	418.30（197.77，896.84）	-0.19(-0.22，-0.16）	25.55（6.33，63.33）	24.08（6.18，64.18）	-0.12(-0.17，-0.08）
Angola	760.96（656.94，865.61）	738.83（636.99，840.23）	-0.10(-0.10，-0.09）	127.48（111.77，145.28）	124.21（109.07，141.23）	-0.08(-0.09，-0.07）	511.90（235.74，1119.38）	570.59（247.01，1310.57）	0.34(0.30，0.38）	28.20（6.57，75.41）	33.40（8.06，92.19）	0.53(0.48，0.58）

Antigua and Barbuda	559.11（482.15，639.66）	545.71（468.40，624.81）	-0.07(-0.08，-0.06）	97.40（85.27，111.61）	95.08（82.70，109.64）	-0.07(-0.08，-0.06）	326.38（156.48，706.05）	311.86（148.11，672.36）	-0.09(-0.11，-0.06）	16.71（4.12，45.60）	15.78（3.80，44.13）	-0.07(-0.12，-0.02）
Argentina	624.58（540.01，715.42）	592.53（512.44，676.59）	-0.18(-0.20，-0.16）	112.19（97.11，128.39）	106.73（92.20，122.29）	-0.17(-0.18，-0.15）	382.45（185.41，805.40）	369.59（176.33，772.22）	-0.09(-0.10，-0.07）	20.88（5.20，56.06）	20.21（5.17，53.04）	-0.06(-0.07，-0.04）
Armenia	655.25（565.59，746.77）	651.84（563.19，741.68）	-0.02(-0.03，-0.01）	114.41（99.75，130.36）	114.00（99.56，130.25）	-0.02(-0.02，-0.01）	383.46（186.79，820.65）	398.52（189.72，838.85）	0.18(0.14，0.22）	19.98（4.91，54.39）	21.34（5.34，56.32）	0.30(0.24，0.36）
Australia	702.82（607.11，797.96）	589.93（513.23，663.34）	-0.60(-0.64，-0.56）	122.93（107.54，138.36）	102.66（90.27，115.36）	-0.62(-0.67，-0.58）	439.01（211.17，915.75）	398.66（187.36，824.12）	-0.32(-0.33，-0.31）	24.20（6.03，63.96）	22.89（5.93，57.75）	-0.16(-0.18，-0.14）
Austria	700.14（604.44，803.39）	654.27（558.12，755.81）	-0.26(-0.29，-0.23）	123.67（107.30，141.71）	116.09（99.55，133.87）	-0.25(-0.27，-0.22）	448.76（212.67，960.88）	425.92（206.34，877.24）	-0.16(-0.17，-0.15）	25.44（6.22，67.72）	24.43（6.28，61.92）	-0.10(-0.11，-0.08）
Azerbaijan	650.14（562.27，745.41）	629.51（541.15，721.59）	-0.13(-0.15，-0.11）	113.74（99.35，129.86）	110.27（96.65，126.18）	-0.13(-0.15，-0.11）	397.18（188.19，849.04）	384.43（179.15，829.85）	-0.09(-0.11，-0.07）	21.19（5.19，58.29）	20.54（5.17，57.09）	-0.04(-0.08，0.01）
Bahamas	557.67（479.49，634.59）	545.68（469.68，625.79）	-0.08(-0.09，-0.07）	97.29（84.85，110.70）	95.28（82.97，109.64）	-0.07(-0.08，-0.07）	322.24（154.69，691.36）	312.32（158.22，666.95）	-0.09(-0.11，-0.07）	16.19（3.87，44.62）	15.76（3.78，43.31）	-0.05(-0.09，-0.02）
Bahrain	789.09（678.43，906.42）	765.69（660.19，875.62）	-0.07(-0.09，-0.06）	133.65（117.28，152.36）	130.61（114.78，148.70）	-0.05(-0.06，-0.04）	520.17（240.47，1181.12）	464.81（218.72，1032.86）	-0.40(-0.44，-0.35）	29.27（7.03，80.92）	25.61（6.06，72.79）	-0.49(-0.56，-0.41）
Bangladesh	461.65（396.87，526.02）	444.05（383.22，506.23）	-0.13(-0.14，-0.12）	82.54（71.13，94.38）	79.47（68.72，90.87）	-0.12(-0.13，-0.11）	291.82（135.94，647.43）	310.82（138.97，735.74）	0.15(0.09，0.22）	15.40（3.61，43.17）	17.27（4.04，50.94）	0.29(0.17，0.41）
Barbados	565.67（492.06，646.55）	542.29（465.00，620.45）	-0.14(-0.16，-0.12）	98.52（86.53，112.22）	94.55（82.58，109.16）	-0.14(-0.16，-0.12）	319.07（153.82，684.03）	313.79（151.07，667.52）	-0.02(-0.08，0.04）	15.84（3.71，43.45）	15.98（3.78，44.24）	0.08(0.00，0.17）
Belarus	670.45（576.02，769.54）	668.51（574.81，769.20）	0.00(-0.01，0.02）	116.59（101.35，133.27）	116.35（101.03，133.43）	0.00(-0.01，0.02）	398.18（190.13，842.13）	393.88（187.68，837.83）	-0.04(-0.06，-0.03）	21.03（5.28，57.99）	20.58（4.99，56.43）	-0.10(-0.12，-0.08）

Belgium	787.50（685.21，897.70）	693.78（598.36，799.83）	-0.46(-0.48，-0.43）	136.89（120.08，155.50）	121.59（105.24，139.55）	-0.43(-0.46，-0.40）	507.61（240.88，1079.06）	457.28（222.55，938.37）	-0.34(-0.38，-0.29）	29.12（7.39，75.81）	26.47（7.10，65.12）	-0.27(-0.33，-0.20）
Belize	580.72（501.76，665.24）	557.96（479.96，636.81）	-0.12(-0.12，-0.11）	101.05（87.86，115.00）	97.17（84.64，110.79）	-0.11(-0.12，-0.11）	330.60（160.46，686.06）	321.18（153.32，672.41）	-0.04(-0.09，0.00）	16.52（4.04，43.14）	16.19（3.93，43.09）	0.02(-0.05，0.09）
Benin	479.13（412.99，546.66）	428.50（376.12，490.81）	-0.25(-0.29，-0.21）	84.72（73.95，97.15）	75.85（65.91，87.05）	-0.25(-0.29，-0.21）	323.19（146.11，695.32）	316.03（141.36，711.21）	-0.05(-0.07，-0.02）	17.98（4.32，48.50）	18.44（4.51，49.83）	0.09(0.06，0.12）
Bermuda	576.86（495.36，659.37）	567.27（489.10，648.40）	-0.06(-0.06，-0.05）	99.80（86.10，114.94）	98.24（84.80，112.71）	-0.06(-0.06，-0.05）	333.74（159.71，708.87）	322.90（159.72，664.50）	-0.12(-0.14，-0.09）	17.02（4.16，46.25）	16.45（4.07，42.53）	-0.10(-0.14，-0.06）
Bhutan	472.18（407.19，539.39）	433.70（372.38，497.86）	-0.27(-0.29，-0.24）	83.88（72.92，96.22）	77.45（67.36，88.68）	-0.25(-0.27，-0.23）	297.89（137.34，638.66）	337.02（145.62，777.24）	0.47(0.43，0.50）	15.51（3.62，42.83）	19.63（4.83，54.56）	0.85(0.79，0.91）
Bolivia (Plurinational State of)	464.08（398.47，530.30）	456.33（392.47，522.57）	-0.02(-0.04，0.01）	83.32（72.16，95.49）	82.05（71.22，94.16）	-0.01(-0.03，0.01）	293.85（136.32，635.66）	294.23（137.35，638.66）	0.02(0.00，0.04）	15.49（3.80，40.10）	15.65（3.84，41.49）	0.05(0.02，0.08）
Bosnia and Herzegovina	640.72（550.04，732.16）	641.05（550.88，735.26）	0.02(-0.01，0.05）	111.71（96.53，128.25）	111.79（96.79，127.27）	0.02(-0.02，0.05）	385.90（182.97，837.09）	374.45（182.06，769.83）	-0.09(-0.11，-0.08）	20.38（5.00，53.64）	19.57（4.76，50.44）	-0.13(-0.16，-0.10）
Botswana	622.87（536.42，712.36）	597.43（511.15，685.77）	-0.10(-0.13，-0.08）	108.19（93.86，123.77）	103.94（90.71，119.61）	-0.10(-0.13，-0.08）	419.53（189.14，950.06）	406.74（186.63，900.91）	-0.06(-0.11，-0.01）	23.01（5.35，67.10）	22.59（5.43，60.14）	0.00(-0.06，0.07）
Brazil	759.73（661.49，864.32）	761.45（661.47，869.62）	-0.05(-0.09，0.00）	129.35（113.26，146.89）	127.08（112.01，144.66）	-0.11(-0.15，-0.07）	511.96（234.64，1114.18）	503.99（236.06，1073.53）	-0.06(-0.07，-0.05）	28.16（7.35，73.41）	27.29（7.15，69.06）	-0.07(-0.09，-0.06）
Brunei Darussalam	581.28（494.56，670.00）	578.96（496.58，663.75）	0.02(-0.02，0.05）	101.91（88.20，118.19）	101.56（88.37，116.86）	0.02(-0.01，0.06）	386.48（177.85，839.95）	396.91（186.35，850.70）	0.17(0.11，0.24）	22.44（5.55，61.65）	23.27（5.71，62.03）	0.23(0.14，0.32）
Bulgaria	657.85（564.21，753.61）	643.45（553.32，739.68）	-0.08(-0.11，-0.06）	114.97（99.80，131.97）	112.43（97.80，128.73）	-0.08(-0.11，-0.06）	384.01（179.87，832.76）	381.00（182.04，808.36）	-0.05(-0.07，-0.02）	19.96（4.68，56.18）	20.04（5.05，55.24）	-0.01(-0.04，0.02）

Burkina Faso	480.35（414.61，547.47）	447.82（385.98，510.39）	-0.25(-0.25，-0.24）	85.31（73.99，97.74）	79.80（69.33，91.77）	-0.23(-0.24，-0.23）	374.24（161.10，852.32）	341.84（149.95，776.62）	-0.34(-0.39，-0.28）	22.14（5.30，60.77）	20.27（4.98，55.69）	-0.35(-0.42，-0.29）
Burundi	632.52（546.31，717.71）	580.93（501.14，662.01）	-0.25(-0.27，-0.23）	109.09（95.51，124.10）	100.50（88.07，114.87）	-0.24(-0.26，-0.22）	412.68（194.13，912.37）	431.79（188.83，1002.56）	0.19(0.16，0.21）	22.55（5.54，61.04）	25.05（6.10，69.42）	0.38(0.35，0.41）
Cabo Verde	465.75（399.93，529.12）	440.96（378.26，500.17）	-0.18(-0.19，-0.17）	82.37（70.98，94.30）	78.13（67.85，89.40）	-0.18(-0.18，-0.17）	303.76（141.96，652.48）	309.03（138.82，694.39）	0.02(0.00，0.04）	16.53（4.02，43.18）	17.54（4.33，46.61）	0.15(0.11，0.19）
Cambodia	683.19（589.81，782.26）	658.61（568.01，754.43）	-0.17(-0.19，-0.15）	116.07（101.32，132.03）	112.21（97.73，128.37）	-0.16(-0.18，-0.14）	412.50（196.15，901.03）	463.50（209.71，1084.98）	0.44(0.41，0.46）	21.16（5.08，58.17）	25.75（6.12，71.34）	0.74(0.70，0.79）
Cameroon	445.65（383.06，507.77）	421.43（362.09，481.18）	-0.18(-0.18，-0.17）	79.70（69.02，91.36）	75.45（65.39，86.37）	-0.18(-0.18，-0.17）	346.06（148.30，758.27）	325.66（145.07，779.51）	-0.23(-0.28，-0.17）	20.46（4.98，54.92）	19.25（4.44，54.68）	-0.24(-0.30，-0.17）
Canada	885.63（781.67，993.27）	792.31（701.61，888.19）	-0.55(-0.64，-0.45）	149.59（134.39，165.87）	132.40（118.02，147.29）	-0.55(-0.61，-0.48）	450.78（230.93，891.10）	417.19（213.00，829.97）	-0.35(-0.40，-0.30）	22.03（5.70，57.72）	21.15（5.57，54.00）	-0.16(-0.18，-0.13）
Central African Republic	800.00（688.59，906.05）	795.18（681.53，900.67）	-0.10(-0.13，-0.07）	132.85（116.29，150.68）	130.87（114.21，148.33）	-0.10(-0.11，-0.08）	575.66（257.05，1267.47）	567.00（253.46，1228.24）	-0.08(-0.11，-0.05）	32.85（7.99，86.60）	31.97（7.50，83.07）	-0.08(-0.13，-0.04）
Chad	472.92（405.50，537.32）	428.14（366.66，486.61）	-0.33(-0.34，-0.32）	84.07（72.82，96.50）	76.45（66.54，87.28）	-0.32(-0.33，-0.31）	312.02（144.48，689.01）	311.23（139.60，709.81）	0.01(0.00，0.03）	17.19（4.16，46.35）	17.76（4.06，48.36）	0.14(0.12，0.16）
Chile	611.01（533.28，694.64）	601.08（518.53，687.09）	-0.02(-0.05，0.01）	109.85（96.31，124.05）	107.87（93.91，123.11）	-0.03(-0.05，0.00）	366.35（176.43，772.10）	361.94（175.97，753.94）	-0.04(-0.05，-0.03）	19.50（4.80，51.90）	19.41（4.93，49.99）	-0.02(-0.04，0.00）
China	703.14（608.36，809.51）	900.82（770.92，1043.22）	0.44(0.35，0.53）	121.11（105.50，137.99）	151.47（131.22，173.34）	0.41(0.33，0.49）	534.47（236.20，1190.60）	562.39（271.16，1238.81）	-0.02(-0.07，0.02）	31.39（7.60，83.63）	30.82（7.88，82.43）	-0.19(-0.23，-0.15）
Colombia	647.46（559.46，736.84）	638.88（549.98，725.16）	-0.03(-0.06，-0.01）	113.71（99.52，130.26）	112.13（97.75，127.51）	-0.03(-0.05，-0.01）	359.12（175.51，757.30）	358.92（176.40，727.46）	-0.01(-0.02，0.00）	17.98（4.49，48.96）	17.86（4.53，44.77）	-0.03(-0.05，-0.02）

Comoros	611.50（525.54，694.46）	582.10（503.27，661.45）	-0.14(-0.16，-0.13）	105.26（92.01，119.38）	100.96（88.05，115.39）	-0.12(-0.13，-0.11）	436.60（198.84，948.69）	472.86（202.81，1099.20）	0.29(0.27，0.31）	25.00（6.06，64.94）	28.16（7.02，75.17）	0.42(0.39，0.45）
Congo	742.92（641.57，845.44）	706.08（641.29，770.22）	-0.14(-0.15，-0.12）	124.97（109.29，142.35）	120.80（109.94，132.53）	-0.08(-0.11，-0.06）	585.15（253.34，1290.63）	572.08（242.82，1281.13）	-0.06(-0.08，-0.04）	34.90（8.36，91.71）	34.49（8.41，89.94）	-0.03(-0.06，0.01）
Cook Islands	644.33（544.78，747.85）	631.64（535.44，728.65）	-0.08(-0.09，-0.07）	111.54（95.15，129.33）	109.53（93.60，126.15）	-0.07(-0.08，-0.06）	446.00（204.64，1012.26）	397.17（185.81，853.89）	-0.34(-0.37，-0.32）	25.23（6.12，70.58）	21.90（5.71，57.63）	-0.42(-0.46，-0.38）
Costa Rica	646.87（556.77，739.05）	631.10（547.39，722.93）	-0.07(-0.09，-0.05）	113.52（98.91，129.64）	110.62（95.96，126.87）	-0.07(-0.09，-0.06）	364.72（178.33，763.04）	356.80（176.36，722.91）	-0.07(-0.08，-0.05）	18.44（4.61，50.01）	18.04（4.54，46.40）	-0.06(-0.08，-0.04）
Croatia	671.05（576.40，770.36）	647.44（556.18，745.36）	-0.09(-0.10，-0.08）	116.47（101.76，133.92）	112.67（98.23，128.39）	-0.08(-0.10，-0.07）	396.94（190.69，870.52）	382.75（182.83，812.48）	-0.11(-0.13，-0.09）	20.87（5.08，57.20）	20.34（4.92，53.88）	-0.09(-0.12，-0.07）
Cuba	521.16（453.79，590.27）	526.06（456.06，596.36）	-0.19(-0.24，-0.13）	92.22（80.31，104.84）	91.41（79.70，103.65）	-0.20(-0.25，-0.16）	306.14（147.67，658.55）	302.91（147.14，645.56）	-0.13(-0.16，-0.11）	15.80（3.79，42.99）	15.40（3.82，42.87）	-0.11(-0.13，-0.10）
Cyprus	690.13（589.98，796.69）	661.56（568.54，762.23）	-0.09(-0.11，-0.07）	122.06（106.32，139.42）	117.37（101.52，134.87）	-0.09(-0.11，-0.07）	494.58（222.59，1135.64）	441.38（204.91，956.95）	-0.37(-0.42，-0.32）	28.94（6.93，81.14）	25.81（6.31，69.51）	-0.37(-0.45，-0.29）
Czechia	643.53（550.56，735.12）	638.18（547.88，727.68）	-0.06(-0.07，-0.05）	111.88（97.48，128.14）	111.23（96.31，127.59）	-0.05(-0.07，-0.04）	385.05（181.59，850.37）	382.68（184.26，794.05）	-0.01(-0.02，0.00）	20.56（5.07，56.00）	20.37（5.02，53.57）	0.02(0.00，0.04）
C么te d'Ivoire	446.24（385.24，513.22）	428.76（367.98，489.19）	-0.13(-0.15，-0.11）	79.55（69.19，91.73）	76.63（66.06，88.28）	-0.12(-0.14，-0.10）	327.53（145.45，731.70）	323.54（141.27，745.74）	-0.03(-0.06，-0.01）	19.40（4.80，51.50）	19.10（4.39，52.57）	-0.04(-0.07，-0.01）
Democratic People's Republic of Korea	631.91（537.88，725.98）	618.92（529.05，710.41）	-0.08(-0.11，-0.06）	110.89（95.65，127.83）	108.50（93.89，125.37）	-0.09(-0.11，-0.06）	422.86（194.83，954.64）	431.35（197.14，950.17）	0.12(0.09，0.15）	23.32（5.35，64.29）	24.21（5.69，67.83）	0.21(0.17，0.26）
Democratic Republic of the Congo	746.90（646.06，847.11）	755.89（656.94，858.40）	0.05(0.02，0.09）	126.38（110.62，143.95）	126.99（111.77，144.57）	0.02(-0.01，0.06）	530.87（234.29，1153.36）	600.24（256.06，1393.32）	0.46(0.42，0.50）	30.13（7.30，80.02）	35.44（8.35，93.78）	0.62(0.55，0.69）

Denmark	586.93（511.27，666.79）	474.71（409.31，539.52）	-0.78(-0.82，-0.73）	102.88（90.99，117.38）	84.34（72.78，96.21）	-0.73(-0.76，-0.69）	402.72（185.09，869.35）	385.44（169.23，831.45）	-0.16(-0.23，-0.10）	23.22（5.78，62.26）	24.12（6.14，60.94）	0.10(0.01，0.19）
Djibouti	638.22（547.66，724.77）	603.12（517.99，686.84）	-0.14(-0.16，-0.13）	109.58（95.81，124.89）	104.02（90.57，118.65）	-0.13(-0.14，-0.11）	457.14（205.06，1006.60）	481.94（209.69，1098.25）	0.18(0.15，0.20）	26.14（6.07，70.91）	28.76（6.68，76.15）	0.31(0.27，0.34）
Dominica	569.19（493.80，648.33）	552.34（475.16，630.23）	-0.09(-0.10，-0.08）	99.50（86.83，113.70）	96.57（83.47，110.40）	-0.09(-0.10，-0.08）	329.64（155.79，712.60）	321.07（152.68，665.17）	-0.07(-0.08，-0.05）	16.67（4.07，46.25）	16.46（4.04，43.80）	-0.01(-0.04，0.02）
Dominican Republic	566.19（491.01，645.07）	572.64（494.88，653.00）	-0.11(-0.15，-0.07）	99.14（86.33，112.62）	99.53（86.68，113.07）	-0.12(-0.15，-0.08）	332.28（160.12，721.37）	324.75（156.90，659.73）	-0.07(-0.10，-0.04）	17.10（4.03，48.37）	16.39（3.85，43.31）	-0.03(-0.08，0.02）
Ecuador	466.53（403.61，534.38）	455.05（388.62，521.67）	-0.04(-0.06，-0.02）	83.15（71.74，95.91）	81.16（69.99，93.40）	-0.03(-0.05，-0.02）	280.71（132.75，606.44）	270.88（129.20，583.75）	-0.09(-0.11，-0.07）	14.53（3.40，39.17）	13.95（3.22，38.79）	-0.11(-0.13，-0.09）
Egypt	754.52（663.10，848.65）	726.90（630.54，823.49）	-0.06(-0.09，-0.03）	130.73（116.04，147.50）	126.46（111.45，144.07）	-0.04(-0.07，-0.02）	491.36（225.85，1097.70）	451.66（212.99，989.38）	-0.29(-0.31，-0.27）	27.08（6.42，77.65）	24.37（5.88，66.23）	-0.38(-0.40，-0.35）
El Salvador	630.69（545.57，718.39）	637.92（552.58，726.95）	0.07(0.05，0.08）	111.03（97.01，126.66）	112.22（98.14，128.45）	0.06(0.05，0.08）	365.91（180.64，780.61）	361.86（179.07，738.85）	-0.03(-0.04，-0.02）	18.68（4.71，48.91）	18.28（4.54，46.04）	-0.07(-0.09，-0.04）
Equatorial Guinea	771.03（667.01，877.69）	736.91（636.47，836.63）	-0.17(-0.18，-0.16）	129.40（113.39，147.34）	124.45（108.79，141.57）	-0.15(-0.16，-0.14）	530.02（238.47，1152.80）	562.36（244.05，1253.71）	0.20(0.15，0.26）	29.58（7.12，80.48）	33.05（7.47，86.51）	0.40(0.32，0.49）
Eritrea	642.78（555.48，731.56）	602.42（518.16，687.76）	-0.20(-0.20，-0.19）	110.64（96.26，126.77）	104.21（90.44，119.18）	-0.18(-0.19，-0.17）	436.83（201.55，978.38）	499.44（210.46，1155.36）	0.44(0.39，0.49）	23.99（5.81，66.86）	30.04（7.02，80.75）	0.73(0.65，0.81）
Estonia	657.29（561.79，755.68）	648.82（559.06，741.98）	-0.01(-0.06，0.04）	113.98（99.49，130.87）	112.78（98.89，128.28）	-0.01(-0.06，0.04）	392.77（190.10，850.86）	402.96（193.00，850.91）	0.10(0.06，0.13）	20.76（4.96，57.16）	21.97（5.44，57.14）	0.19(0.17，0.20）
Eswatini	604.07（520.51，691.04）	579.24（499.45，661.83）	-0.14(-0.16，-0.13）	105.08（91.43，120.24）	101.07（88.65，115.80）	-0.13(-0.14，-0.11）	419.59（187.58，922.54）	420.94（191.51，955.49）	0.10(0.07，0.12）	23.35（5.75，64.74）	23.38（5.48，63.37）	0.18(0.13，0.23）

Ethiopia	638.14（554.00，727.32）	586.55（509.54，668.44）	-0.24(-0.28，-0.21）	110.74（96.77，126.37）	102.87（90.07，116.73）	-0.21(-0.24，-0.17）	472.01（207.19，1059.78）	482.27（208.72，1084.59）	0.08(0.01，0.15）	26.85（6.56，74.38）	28.67（7.39，74.05）	0.23(0.15，0.31）
Fiji	650.89（555.43，752.89）	637.89（544.12，735.60）	-0.09(-0.10，-0.07）	112.73（96.97，130.52）	110.65（95.50，127.91）	-0.08(-0.09，-0.07）	425.25（192.76，954.02）	404.94（191.74，883.50）	-0.20(-0.22，-0.18）	23.56（5.67，67.01）	22.54（5.70，59.17）	-0.21(-0.25，-0.18）
Finland	690.51（593.57，781.35）	616.90（523.13，709.76）	-0.40(-0.41，-0.39）	121.24（105.54，137.41）	108.45（92.52，124.81）	-0.40(-0.41，-0.39）	476.52（222.43，1036.19）	438.11（204.07，904.23）	-0.25(-0.28，-0.23）	28.12（7.30，73.19）	26.14（7.00，64.65）	-0.18(-0.22，-0.14）
France	553.50（491.88，615.65）	525.22（458.80，593.27）	-0.20(-0.23，-0.16）	99.39（89.46，109.62）	93.63（82.25，105.56）	-0.22(-0.26，-0.19）	388.02（176.76，835.11）	366.86（171.08，763.86）	-0.18(-0.20，-0.17）	22.98（5.79，60.15）	21.69（5.61，54.30）	-0.18(-0.20，-0.16）
Gabon	753.38（649.45，860.80）	734.43（633.02，837.16）	-0.05(-0.06，-0.04）	125.84（109.61，143.75）	123.01（107.72，140.92）	-0.05(-0.07，-0.04）	592.49（256.17，1284.17）	588.29（255.87，1336.53）	-0.02(-0.03，-0.01）	35.33（8.96，91.15）	35.44（8.87，93.89）	0.01(0.00，0.01）
Gambia	473.34（405.47，538.30）	436.83（374.25，499.13）	-0.27(-0.28，-0.26）	84.22（73.25，96.89）	77.92（67.57，89.88）	-0.26(-0.27，-0.25）	341.30（154.14，743.82）	341.38（142.91，788.35）	-0.07(-0.12，-0.02）	19.76（4.77，53.04）	20.15（4.74，54.10）	0.00(-0.05，0.04）
Georgia	651.05（562.33，741.66）	651.46（562.89，743.97）	0.01(0.00，0.03）	113.74（99.03，129.23）	113.98（99.53，129.49）	0.01(0.00，0.02）	391.78（185.70，839.12）	398.83（191.96，827.23）	0.06(0.02，0.11）	20.59（5.04，56.28）	21.11（5.51，55.26）	0.09(0.00，0.17）
Germany	809.57（715.41，909.95）	820.50（713.82，930.77）	0.06(0.03，0.08）	145.12（130.54，161.52）	142.13（124.57，159.72）	-0.10(-0.13，-0.07）	510.92（245.56，1063.38）	508.64（250.83，1028.42）	-0.05(-0.09，-0.01）	29.50（7.52，73.80）	29.25（7.91，69.29）	-0.07(-0.12，-0.02）
Ghana	429.93（369.00，491.63）	419.76（360.59，476.55）	-0.06(-0.07，-0.05）	76.92（66.34，88.65）	75.09（64.72，86.42）	-0.06(-0.07，-0.05）	297.50（135.33，682.06）	327.77（144.37，776.72）	0.33(0.29，0.38）	16.74（4.02，46.59）	19.38（4.60，54.77）	0.50(0.45，0.55）
Greece	703.66（602.22，809.60）	671.46（575.61，767.68）	-0.13(-0.15，-0.11）	124.20（107.47，142.66）	119.17（103.16，136.72）	-0.11(-0.13，-0.09）	442.61（209.80，948.67）	429.55（206.36，907.38）	-0.10(-0.11，-0.09）	24.85（6.22，66.57）	24.23（6.08，61.76）	-0.09(-0.11，-0.08）
Greenland	805.34（683.87，935.07）	786.97（673.97，906.26）	-0.09(-0.11，-0.06）	138.09（119.55，159.13）	135.63（117.63，156.11）	-0.07(-0.10，-0.05）	485.34（233.26，1051.99）	470.67（229.50，964.22）	-0.08(-0.10，-0.05）	26.47（6.28，71.42）	25.68（6.77，65.52）	-0.06(-0.09，-0.03）

Grenada	570.58（491.20，651.70）	562.63（487.52，643.75）	-0.02(-0.05，0.01）	99.53（86.87，113.92）	98.02（85.92，112.22）	-0.02(-0.05，0.01）	337.48（163.41，723.45）	326.57（158.38，707.55）	0.08(0.04，0.13）	17.70（4.20，47.39）	16.75（3.95，46.67）	0.17(0.07，0.26）
Guam	632.58（536.40，731.64）	629.81（535.65，725.95）	-0.03(-0.04，-0.02）	109.67（94.24，128.33）	108.97（93.85，125.54）	-0.03(-0.03，-0.02）	421.56（191.40，927.54）	368.22（185.54，718.58）	-0.43(-0.47，-0.39）	23.14（5.54，63.24）	18.31（5.14，42.90）	-0.70(-0.77，-0.63）
Guatemala	646.74（561.57，736.53）	639.86（553.28，730.62）	-0.02(-0.03，0.00）	113.72（99.12，129.64）	112.54（98.73，127.70）	-0.02(-0.03，0.00）	369.03（176.80，798.15）	360.29（173.83，750.81）	-0.09(-0.10，-0.07）	18.90（4.49，51.79）	18.29（4.47，49.71）	-0.13(-0.16，-0.11）
Guinea	466.54（403.27，530.43）	438.63（379.03，498.93）	-0.20(-0.22，-0.18）	83.16（72.43，95.24）	78.39（67.86，90.01）	-0.19(-0.21，-0.18）	310.30（137.96，669.31）	321.03（144.10，737.33）	0.12(0.09，0.15）	17.04（4.13，46.39）	18.50（4.17，50.59）	0.31(0.28，0.35）
Guinea-Bissau	443.86（380.49，503.71）	431.13（370.44，493.27）	-0.08(-0.09，-0.07）	79.09（68.36，91.21）	77.05（66.66，89.04）	-0.07(-0.08，-0.06）	329.06（142.99，735.28）	338.39（143.13，800.84）	0.10(0.08，0.12）	18.90（4.65，51.96）	19.74（4.59，54.93）	0.17(0.13，0.20）
Guyana	554.65（477.24，635.00）	549.03（471.54，629.36）	-0.04(-0.05，-0.03）	97.11（84.10，111.09）	96.25（83.56，110.32）	-0.04(-0.05，-0.03）	303.85（145.81，649.61）	307.66（149.80，659.64）	0.00(-0.07，0.08）	15.25（3.67，41.62）	15.56（3.64，41.96）	0.01(-0.10，0.13）
Haiti	586.32（506.12，662.48）	541.86（468.24，619.70）	-0.28(-0.29，-0.26）	102.30（89.46，116.07）	94.82（82.42，108.66）	-0.26(-0.28，-0.25）	340.60（164.07，747.26）	322.24（153.66，721.17）	-0.17(-0.18，-0.16）	17.13（4.07，48.43）	16.49（3.62，46.24）	-0.10(-0.11，-0.09）
Honduras	653.72（564.16，740.88）	641.67（553.16，730.68）	-0.06(-0.07，-0.06）	115.03（100.77，131.35）	113.19（98.75，129.15）	-0.05(-0.06，-0.05）	396.47（190.93，839.37）	427.72（199.15，959.38）	0.24(0.19，0.29）	20.35（5.00，54.04）	22.97（5.57，62.96）	0.36(0.27，0.44）
Hungary	645.34（551.33，739.87）	638.10（548.27，732.18）	-0.02(-0.06，0.02）	112.52（98.61，128.63）	111.47（96.69，127.63）	-0.02(-0.05，0.02）	385.80（181.75，842.77）	380.96（184.01，785.57）	-0.01(-0.02，0.01）	20.73（5.04，57.34）	20.10（4.89，53.41）	-0.06(-0.07，-0.05）
Iceland	761.00（654.06，859.57）	683.68（586.64，767.04）	-0.40(-0.42，-0.38）	135.01（117.98，152.05）	122.36（106.34，137.53）	-0.36(-0.38，-0.35）	454.55（223.51，942.86）	429.58（210.02，870.83）	-0.16(-0.19，-0.13）	24.68（6.16，64.12）	24.30（6.45，60.39）	0.00(-0.05，0.05）
India	440.05（382.11，503.90）	436.13（376.02，501.15）	-0.16(-0.21，-0.12）	79.45（68.69，90.82）	78.92（68.29，90.58）	-0.16(-0.21，-0.11）	262.07（122.60，600.90）	305.73（135.42，676.67）	0.52(0.48，0.56）	13.35（3.01，38.49）	16.98（4.05，46.35）	0.89(0.82，0.95）

Indonesia	678.72（587.97，777.61）	662.13（571.11，761.15）	-0.08(-0.10，-0.05）	115.97（101.07，132.00）	113.31（98.38，129.52）	-0.07(-0.09，-0.05）	365.07（178.21，779.12）	422.00（194.37，933.56）	0.41(0.35，0.48）	17.55（4.10，47.58）	22.97（5.62，63.06）	0.77(0.68，0.87）
Iran (Islamic Republic of)	804.38（692.92，921.70）	775.51（671.84，887.85）	-0.11(-0.12，-0.10）	137.67（120.79，156.35）	133.73（117.53，151.85）	-0.08(-0.09，-0.07）	506.44（241.22，1099.44）	473.64（225.81，1003.84）	-0.25(-0.26，-0.23）	27.30（6.77，73.62）	25.18（6.28，66.26）	-0.29(-0.32，-0.27）
Iraq	796.72（678.19，914.39）	759.67（652.95，868.12）	-0.19(-0.20，-0.17）	135.28（117.85，154.35）	129.62（113.05，148.98）	-0.17(-0.19，-0.16）	490.69（231.46，1051.37）	473.14（223.03，1007.50）	-0.20(-0.23，-0.17）	26.16（6.43，67.77）	25.77（6.39，66.80）	-0.16(-0.19，-0.12）
Ireland	691.14（590.07，791.51）	629.49（535.55，725.24）	-0.31(-0.34，-0.29）	122.27（105.91，140.72）	111.99（95.75，128.99）	-0.30(-0.32，-0.27）	444.18（210.96，962.81）	417.27（199.94，861.14）	-0.17(-0.20，-0.15）	25.29（6.23，67.37）	24.09（6.25，60.28）	-0.09(-0.12，-0.07）
Israel	678.13（581.61，781.72）	636.97（546.32，734.42）	-0.21(-0.22，-0.21）	120.30（103.78，138.38）	113.13（97.01，130.26）	-0.21(-0.22，-0.20）	433.13（204.21，934.20）	421.50（202.20，878.99）	-0.09(-0.12，-0.07）	24.43（6.15，64.85）	24.29（6.31，60.01）	-0.02(-0.06，0.02）
Italy	692.96（589.47，798.14）	759.15（652.54，869.83）	0.13(-0.01，0.27）	116.29（98.96，133.09）	134.76（116.74，153.65）	0.28(0.09，0.47）	516.62（233.92，1110.05）	507.40（243.87，1036.59）	-0.03(-0.06，0.00）	31.50（8.08，81.26）	29.75（8.10，72.88）	-0.09(-0.13，-0.05）
Jamaica	643.37（553.27，732.56）	609.75（523.06，695.65）	-0.20(-0.21，-0.18）	110.63（96.95，126.27）	104.77（91.01，120.07）	-0.20(-0.21，-0.18）	340.78（169.90，704.29）	328.53（164.60，683.23）	-0.14(-0.17，-0.11）	16.42（3.99，45.09）	16.02（4.00，43.04）	-0.09(-0.12，-0.05）
Japan	649.68（564.43，739.33）	674.10（584.78，770.24）	0.30(0.24，0.36）	115.81（101.01，131.94）	117.23（102.05，133.68）	0.20(0.15，0.26）	460.57（214.58，985.33）	456.00（221.79，920.17）	0.03(0.01，0.06）	27.18（7.14，69.33）	26.33（7.39，62.16）	-0.07(-0.10，-0.04）
Jordan	784.45（675.13，901.07）	786.72（675.70，906.66）	0.05(0.01，0.09）	133.38（116.04，152.85）	134.51（116.99，153.81）	0.07(0.03，0.11）	487.44（236.71，1035.26）	450.02（219.99，960.28）	-0.29(-0.31，-0.26）	26.24（6.72，70.10）	23.37（5.78，64.07）	-0.43(-0.46，-0.40）
Kazakhstan	649.97（561.89，746.48）	634.04（542.82，724.42）	-0.08(-0.11，-0.06）	113.75（99.27，130.56）	111.10（96.76，126.81）	-0.08(-0.10，-0.05）	390.89（184.64，843.79）	371.56（177.75，812.03）	-0.25(-0.30，-0.20）	20.79（5.15，55.93）	19.44（4.58，55.94）	-0.33(-0.39，-0.27）
Kenya	613.02（529.32，697.76）	605.07（525.16，689.45）	-0.04(-0.07，-0.02）	106.99（93.29，121.75）	105.14（91.55，119.62）	-0.06(-0.08，-0.04）	424.09（190.83，967.98）	480.57（205.49，1052.02）	0.46(0.44，0.47）	23.88（5.75，66.29）	28.70（7.01，72.33）	0.67(0.65，0.70）

Kiribati	699.11（598.21，802.88）	701.89（602.78，803.92）	0.00(-0.03，0.04）	121.16（104.74，139.34）	121.84（106.12，138.96）	0.01(-0.02，0.04）	444.73（206.23，1011.07）	484.20（221.09，1072.53）	0.27(0.24，0.31）	23.75（5.39，63.95）	27.39（6.35，72.65）	0.46(0.42，0.50）
Kuwait	820.13（709.23，935.64）	771.61（668.04，878.03）	-0.20(-0.22，-0.17）	137.77（120.63，157.00）	131.46（115.44，149.34）	-0.15(-0.17，-0.13）	504.17（243.96，1060.09）	457.75（224.95，949.83）	-0.33(-0.40，-0.26）	26.98（6.93，67.44）	24.36（6.33，63.30）	-0.36(-0.47，-0.24）
Kyrgyzstan	645.42（559.15，733.86）	649.99（562.04，741.22）	0.05(0.04，0.06）	112.94（98.41，128.40）	113.88（99.39，130.07）	0.05(0.04，0.06）	396.12（189.20，837.35）	386.99（186.91，800.00）	-0.08(-0.10，-0.07）	21.24（5.22，56.57）	20.09（5.07，52.52）	-0.21(-0.24，-0.19）
Lao People's Democratic Republic	674.31（585.55，768.35）	655.85（567.01，749.46）	-0.07(-0.09，-0.06）	114.26（100.61，130.80）	111.82（97.43，127.89）	-0.06(-0.07，-0.04）	389.09（188.34，847.42）	407.47（187.60，907.38）	0.19(0.16，0.21）	19.59（4.60，54.66）	21.60（5.14，60.21）	0.36(0.32，0.40）
Latvia	659.29（565.04，757.79）	660.59（570.40，758.14）	0.04(0.02，0.07）	114.61（99.77，131.15）	114.97（99.97，131.00）	0.04(0.02，0.06）	392.89（186.13，839.82）	392.16（187.44，818.04）	0.02(-0.01，0.05）	20.83（5.03，57.58）	20.79（5.08，55.96）	0.02(-0.01，0.04）
Lebanon	826.34（713.64，946.49）	828.25（710.40，948.14）	-0.02(-0.04，-0.01）	139.66（122.17，159.68）	140.48（122.53，159.62）	0.00(-0.01，0.01）	473.64（232.18，975.21）	460.13（229.84，949.69）	-0.07(-0.15，0.01）	24.70（6.34，67.28）	23.83（6.08，62.44）	-0.05(-0.17，0.06）
Lesotho	636.50（551.08，723.89）	625.49（537.97，713.46）	-0.03(-0.04，-0.02）	110.50（95.98，126.11）	108.92（94.83，124.74）	-0.02(-0.03，-0.01）	413.57（194.21，873.14）	430.58（193.02，983.97）	0.25(0.18，0.32）	22.14（5.27，60.07）	23.44（5.96，65.49）	0.40(0.29，0.51）
Liberia	435.63（377.36，494.91）	419.94（360.58，478.03）	-0.12(-0.13，-0.11）	77.77（67.19，89.07）	75.16（64.95，86.14）	-0.11(-0.12，-0.10）	308.34（138.41，703.75）	307.31（137.60，719.91）	0.03(0.00，0.06）	17.77（4.21，50.37）	17.86（4.03，49.79）	0.07(0.03，0.11）
Libya	805.14（695.68，912.97）	762.64（660.37，872.63）	-0.18(-0.19，-0.16）	137.01（120.05，155.79）	130.47（114.04，148.13）	-0.16(-0.18，-0.14）	550.60（256.35，1138.12）	502.17（241.77，1123.68）	-0.26(-0.29，-0.23）	30.80（8.02，76.76）	28.04（6.73，75.56）	-0.21(-0.26，-0.16）
Lithuania	650.56（561.65，749.04）	648.52（560.43，740.20）	0.02(-0.01，0.05）	113.06（98.58，129.94）	112.73（98.22，129.20）	0.02(-0.01，0.05）	391.67（185.38，838.74）	393.44（190.01，837.06）	0.01(-0.01，0.04）	20.74（4.96，57.05）	21.02（5.20，56.80）	0.04(0.02，0.06）
Luxembourg	551.09（468.86，636.23）	473.86（402.43，551.80）	-0.53(-0.60，-0.46）	100.78（86.37，116.34）	87.36（74.31，101.13）	-0.51(-0.57，-0.44）	325.91（156.55，696.57）	308.22（144.66，646.56）	-0.17(-0.20，-0.15）	17.61（4.20，47.35）	17.72（4.55，44.71）	0.06(0.02，0.10）

Madagascar	606.36（524.14，687.06）	574.95（493.29，652.02）	-0.16(-0.17，-0.14）	105.10（91.62，120.32）	99.76（86.80，114.13）	-0.15(-0.17，-0.14）	360.43（172.97，789.78）	365.63（170.82，805.91）	0.06(0.05，0.08）	18.82（4.53，52.84）	19.74（4.71，52.59）	0.16(0.14，0.18）
Malawi	607.88（526.91，694.55）	605.24（517.75，689.89）	0.00(-0.01，0.02）	105.40（91.35，120.59）	104.71（90.82，119.89）	-0.01(-0.02，0.01）	422.24（194.25，958.33）	463.75（206.32，1030.55）	0.34(0.31，0.37）	23.75（5.81，68.09）	27.07（6.32，73.02）	0.49(0.45，0.53）
Malaysia	695.54（601.08，791.73）	657.55（561.66，750.29）	-0.13(-0.16，-0.11）	116.13（101.28，132.46）	111.29（95.99，127.46）	-0.12(-0.13，-0.10）	445.10（211.57，957.71）	445.34（203.45，976.51）	-0.05(-0.09，0.00）	23.63（6.21，62.13）	24.39（6.02，65.59）	0.02(-0.06，0.10）
Maldives	647.37（556.97，738.29）	667.30（572.03，764.23）	0.10(0.07，0.13）	109.84（95.49，126.07）	113.22（98.24，129.67）	0.10(0.07，0.13）	362.78（172.44，801.40）	384.86（186.59，802.36）	0.18(0.13，0.23）	17.74（4.14，50.44）	19.55（4.80，52.57）	0.30(0.21，0.39）
Mali	460.80（396.23，525.50）	439.59（379.27，499.83）	-0.15(-0.15，-0.14）	81.91（71.19，93.65）	78.55（68.40，89.84）	-0.13(-0.14，-0.12）	366.70（156.49，838.12）	363.61（153.57，846.85）	0.01(-0.02，0.04）	22.03（5.35，60.22）	21.85（5.18，61.16）	0.04(0.00，0.08）
Malta	695.45（597.76，804.83）	642.06（551.42，738.48）	-0.24(-0.27，-0.21）	122.80（106.32，141.45）	113.91（98.03，130.83）	-0.22(-0.25，-0.20）	442.14（207.61，934.70）	421.98（204.82，872.32）	-0.19(-0.22，-0.17）	24.71（6.13，65.73）	24.00（6.24，59.13）	-0.17(-0.20，-0.13）
Marshall Islands	617.40（523.92，711.85）	594.07（502.31，685.62）	-0.13(-0.13，-0.12）	107.43（92.62，124.49）	103.94（89.43，120.50）	-0.11(-0.11，-0.10）	450.32（202.99，1044.26）	420.98（191.27，996.32）	-0.24(-0.25，-0.23）	25.92（6.15，72.44）	24.17（5.79，69.03）	-0.27(-0.28，-0.25）
Mauritania	467.98（400.78，533.61）	433.09（374.33，493.21）	-0.25(-0.26，-0.24）	83.53（71.72，96.45）	77.53（67.33，88.83）	-0.24(-0.25，-0.23）	331.16（148.76，747.32）	329.08（146.78，795.20）	-0.07(-0.09，-0.05）	18.80（4.37，52.50）	19.08（4.23，56.09）	-0.02(-0.04，0.01）
Mauritius	663.44（568.33，762.47）	657.17（567.03，748.30）	0.00(-0.02，0.03）	112.36（97.71，130.05）	111.73（96.55，127.55）	0.01(-0.02，0.04）	410.23（193.18，896.71）	390.08（186.92，832.38）	-0.18(-0.25，-0.11）	21.92（5.34，58.62）	20.30（5.00，55.30）	-0.31(-0.40，-0.22）
Mexico	585.32（506.17，669.22）	534.20（460.41，612.87）	-0.20(-0.24，-0.16）	106.03（91.84，120.63）	97.19（84.27，111.26）	-0.19(-0.23，-0.15）	309.40（150.68，660.70）	296.84（144.66，642.67）	-0.11(-0.12，-0.10）	15.10（3.65，41.69）	14.80（3.49，41.39）	-0.07(-0.09，-0.06）
Micronesia (Federated States of)	691.53（590.09，798.37）	695.38（594.38，799.43）	0.05(0.04，0.06）	119.80（102.66，137.56）	120.71（104.84，139.46）	0.06(0.05，0.07）	476.07（218.92，1100.61）	468.56（217.61，1082.02）	-0.04(-0.07，0.00）	26.52（6.41，74.67）	26.24（6.29，73.73）	-0.03(-0.07，0.01）

Monaco	696.22（595.18，799.43）	639.25（542.47，742.18）	-0.30(-0.30，-0.29）	123.30（106.75，141.67）	113.72（97.81，131.33）	-0.28(-0.28，-0.27）	453.72（215.99，1003.31）	450.66（212.60，919.67）	-0.01(-0.03，0.01）	25.82（6.09，70.75）	27.22（6.93，64.58）	0.20(0.17，0.24）
Mongolia	653.39（565.27，748.42）	660.02（573.53，749.23）	0.05(0.04，0.06）	114.40（99.85，130.62）	115.83（101.21，132.52）	0.05(0.04，0.06）	412.86（193.85，881.39）	402.27（192.91，881.94）	-0.15(-0.18，-0.13）	22.65（5.65，59.43）	21.66（5.32，59.88）	-0.25(-0.29，-0.20）
Montenegro	656.91（570.25，753.22）	639.32（547.61，729.15）	-0.13(-0.17，-0.08）	114.61（100.10，131.64）	111.66（97.13，128.15）	-0.12(-0.16，-0.08）	392.24（185.50，838.01）	383.69（179.33，823.48）	-0.09(-0.12，-0.06）	20.59（4.90，55.78）	20.47（5.01，54.91）	0.00(-0.07，0.06）
Morocco	804.49（696.28，914.48）	748.52（644.54，856.83）	-0.24(-0.25，-0.23）	136.84（120.00，155.37）	127.85（111.31，146.15）	-0.23(-0.24，-0.22）	498.06（241.30，1079.72）	479.67（223.95，1061.79）	-0.09(-0.11，-0.07）	26.47（6.53，70.71）	26.13（6.16，70.40）	0.01(-0.02，0.05）
Mozambique	624.73（540.32，711.49）	605.09（520.43，691.27）	-0.08(-0.10，-0.06）	107.72（94.15，122.63）	104.33（90.49，119.04）	-0.08(-0.10，-0.07）	456.75（201.73，1027.76）	490.46（207.92，1152.97）	0.35(0.31，0.38）	26.85（6.81，72.77）	29.76（7.04，83.45）	0.49(0.44，0.54）
Myanmar	720.54（625.38，824.31）	663.53（571.48，757.76）	-0.28(-0.29，-0.26）	122.22（107.45，139.08）	113.09（99.06，128.82）	-0.27(-0.28，-0.25）	390.49（192.75，822.66）	407.47（187.74，911.87）	0.12(0.09，0.14）	19.06（4.60，51.48）	21.50（5.04，60.71）	0.36(0.32，0.41）
Namibia	616.32（530.85，701.68）	593.68（514.23，676.82）	-0.11(-0.12，-0.10）	107.16（93.53，121.95）	103.46（89.84，117.70）	-0.11(-0.12，-0.09）	402.65（187.31，907.77）	432.91（196.45，1035.86）	0.24(0.22，0.26）	21.69（5.39，62.32）	24.26（5.55，69.21）	0.39(0.36，0.43）
Nauru	632.73（535.53，733.18）	652.63（555.94，749.51）	0.07(0.03，0.12）	110.81（95.43，128.03）	113.97（98.71，131.23）	0.06(0.02，0.10）	447.11（201.16，1005.34）	448.40（201.57，1042.39）	0.03(0.00，0.05）	25.22（5.94，69.48）	25.19（5.89，71.70）	0.04(0.01，0.06）
Nepal	510.81（441.59，585.55）	454.19（392.41，519.26）	-0.46(-0.49，-0.43）	91.22（79.31，104.24）	81.31（70.51，93.70）	-0.45(-0.48，-0.42）	280.91（139.12，594.87）	310.85（140.50，699.68）	0.34(0.29，0.39）	13.97（3.22，38.57）	17.32（4.03，48.09）	0.77(0.70，0.84）
Netherlands	711.18（625.56，787.39）	700.28（610.56，794.56）	0.01(-0.03，0.04）	127.60（112.23，140.58）	121.79（106.84，137.86）	-0.14(-0.17，-0.11）	482.11（228.55，1010.99）	474.98（223.60，983.43）	-0.03(-0.04，-0.02）	28.41（7.26，71.50）	27.94（7.49，70.46）	-0.03(-0.05，-0.01）
New Zealand	728.11（627.47，840.73）	684.59（589.02，787.91）	-0.24(-0.29，-0.20）	128.02（110.92，146.45）	120.84（104.92，138.57）	-0.22(-0.26，-0.19）	465.69（219.62，984.68）	441.17（210.93，909.28）	-0.21(-0.25，-0.16）	26.12（6.67，68.20）	24.83（6.58，64.00）	-0.19(-0.23，-0.14）

Nicaragua	660.24（569.18，749.74）	654.85（569.17，748.60）	0.00(-0.01，0.01）	115.45（101.47，131.74）	114.62（100.58，131.05）	0.00(-0.01，0.01）	365.48（181.44，755.05）	356.84（181.81，732.29）	-0.05(-0.07，-0.02）	18.28（4.67，47.36）	17.57（4.39，46.45）	-0.08(-0.12，-0.05）
Niger	470.52（404.38，536.04）	439.52（380.44，501.25）	-0.22(-0.23，-0.21）	83.26（72.29，95.94）	78.36（68.05，90.06）	-0.20(-0.21，-0.19）	330.91（147.98，724.22）	331.02（147.57，734.60）	0.07(0.03，0.11）	18.87（4.68，50.47）	19.32（4.53，52.21）	0.18(0.12，0.25）
Nigeria	417.35（360.95，476.61）	377.62（326.26，432.75）	-0.35(-0.39，-0.31）	75.45（65.40，86.12）	68.83（59.58，78.60）	-0.31(-0.36，-0.27）	296.84（131.50，656.93）	309.45（131.77，715.01）	0.22(0.15，0.28）	17.36（4.33，47.96）	19.01（4.63，51.88）	0.43(0.35，0.50）
Niue	635.45（538.72，734.68）	616.47（519.99，710.42）	-0.13(-0.14，-0.11）	110.25（95.02，127.06）	107.28（91.33，123.83）	-0.11(-0.12，-0.09）	478.71（213.11，1086.49）	434.13（196.03，954.18）	-0.33(-0.35，-0.32）	27.77（6.79，75.94）	24.96（6.15，65.56）	-0.37(-0.39，-0.35）
North Macedonia	641.10（548.24，734.12）	636.56（546.06，726.34）	-0.01(-0.02，0.01）	111.85（97.22，128.30）	111.12（96.41，127.65）	-0.01(-0.02，0.00）	371.22（179.98，794.46）	373.95（177.20，816.33）	-0.01(-0.03，0.02）	19.21（4.79，52.96）	19.67（4.83，54.02）	0.02(-0.03，0.06）
Northern Mariana Islands	630.79（530.47，734.74）	621.13（528.08，721.28）	-0.04(-0.06，-0.03）	109.52（93.43，127.91）	107.94（92.46，125.17）	-0.04(-0.05，-0.03）	420.69（193.45，939.21）	410.96（189.74，911.58）	-0.05(-0.09，-0.01）	23.33（5.66，64.94）	22.81（5.53，62.38）	-0.05(-0.11，0.01）
Norway	769.17（664.98，878.38）	639.18（549.63，735.45）	-0.67(-0.72，-0.63）	136.54（119.38，155.02）	114.30（98.69，131.41）	-0.64(-0.69，-0.60）	477.46（227.82，1011.83）	427.76（202.41，901.90）	-0.38(-0.41，-0.35）	26.54（6.74，69.38）	24.87（6.46，63.48）	-0.21(-0.26，-0.16）
Oman	787.34（678.30，903.05）	728.25（624.77，840.49）	-0.24(-0.28，-0.19）	132.92（116.54，151.46）	124.36（108.14，143.21）	-0.20(-0.24，-0.15）	512.85（245.40，1088.66）	472.28（223.68，1076.18）	-0.26(-0.36，-0.17）	28.27（6.99，74.05）	26.12（6.65，71.88）	-0.24(-0.37，-0.11）
Pakistan	464.81（400.97，530.52）	433.58（374.32，499.28）	-0.25(-0.26，-0.24）	84.44（73.08，97.03）	78.80（67.99，90.38）	-0.25(-0.26，-0.24）	310.92（138.64，710.19）	333.84（140.09，758.92）	0.16(0.12，0.20）	17.08（4.08，48.08）	19.59（4.73，53.82）	0.36(0.30，0.42）
Palau	616.08（520.12，716.46）	594.28（501.85，687.51）	-0.11(-0.12，-0.10）	107.16（91.89，124.54）	103.98（88.42，120.53）	-0.09(-0.09，-0.08）	435.69（194.62，990.99）	389.68（175.49，883.59）	-0.33(-0.37，-0.30）	24.72（6.01，68.55）	21.70（5.15，62.95）	-0.39(-0.44，-0.34）
Palestine	803.58（690.88，915.33）	773.83（669.58，881.66）	-0.14(-0.16，-0.11）	136.47（119.22，154.40）	131.55（115.83，150.08）	-0.13(-0.16，-0.10）	494.79（232.85，1111.89）	461.77（223.02，1011.99）	-0.23(-0.29，-0.17）	26.68（6.42，73.99）	24.68（6.05，67.81）	-0.25(-0.32，-0.18）

Panama	628.65（543.24，715.98）	620.46（530.21，708.40）	-0.05(-0.05，-0.04）	110.21（95.80，125.86）	108.64（94.58，124.54）	-0.05(-0.06，-0.04）	349.94（172.43，743.98）	348.86（174.09，698.83）	0.00(-0.02，0.02）	17.53（4.34，46.56）	17.60（4.37，44.67）	0.04(0.01，0.07）
Papua New Guinea	693.65（602.01，797.17）	649.65（557.31，742.38）	-0.26(-0.29，-0.24）	120.02（105.08，136.87）	112.89（97.86，129.70）	-0.25(-0.28，-0.22）	413.72（196.86，911.19）	393.40（182.94，868.87）	-0.21(-0.24，-0.19）	21.26（5.20，58.69）	20.56（4.68，59.98）	-0.15(-0.17，-0.13）
Paraguay	729.89（629.40，832.72）	687.12（593.84，779.77）	-0.22(-0.23，-0.21）	123.19（107.15，139.90）	115.77（101.31，132.33）	-0.22(-0.23，-0.21）	467.69（223.43，985.68）	465.79（215.80，974.39）	0.00(-0.02，0.01）	24.93（6.03，64.42）	25.62（6.40，65.43）	0.13(0.10，0.17）
Peru	441.69（382.66，505.23）	436.46（377.50，501.78）	-0.09(-0.11，-0.07）	78.97（68.46，90.10）	78.28（67.57，89.77）	-0.09(-0.11，-0.06）	274.95（128.33，602.68）	268.50（129.48，574.76）	-0.14(-0.17，-0.11）	14.42（3.48，39.42）	13.90（3.49，37.12）	-0.17(-0.21，-0.13）
Philippines	691.99（601.34，792.19）	662.94（575.83，762.08）	-0.17(-0.20，-0.15）	119.03（104.70，135.62）	113.91（99.74，130.00）	-0.18(-0.19，-0.16）	393.94（186.07，844.77）	410.29（191.00，846.37）	0.15(0.14，0.17）	20.02（4.85，56.29）	21.49（5.23，54.38）	0.29(0.26，0.32）
Poland	683.36（589.12，787.44）	645.55（558.48，741.84）	-0.19(-0.20，-0.18）	120.58（105.09，137.37）	114.27（99.44，130.40）	-0.18(-0.19，-0.17）	406.31（192.93，884.27）	394.77（187.59，844.38）	-0.09(-0.10，-0.08）	21.47（5.29，58.89）	21.08（5.36，55.12）	-0.05(-0.07，-0.03）
Portugal	672.60（577.21，772.67）	659.36（561.71，757.50）	-0.04(-0.06，-0.02）	118.74（102.91，136.33）	116.97（100.45，134.76）	-0.02(-0.04，0.00）	444.35（208.98，981.30）	432.79（205.33，900.75）	-0.07(-0.09，-0.05）	25.65（6.17，70.72）	24.79（6.47，62.09）	-0.09(-0.12，-0.07）
Puerto Rico	576.17（496.29，659.73）	563.62（484.51，646.62）	-0.08(-0.09，-0.07）	99.86（86.41，113.62）	97.80（84.88，112.14）	-0.08(-0.09，-0.07）	332.94（158.99，712.73）	318.47（158.35，648.17）	-0.16(-0.17，-0.14）	16.88（4.05，46.86）	15.96（4.06，40.88）	-0.18(-0.20，-0.16）
Qatar	761.25（655.07，876.05）	740.91（636.99，852.34）	-0.02(-0.06，0.02）	129.42（112.13，147.88）	126.56（110.10，145.40）	-0.01(-0.05，0.03）	518.27（237.90，1181.00）	467.95（215.80，1029.22）	-0.31(-0.36，-0.26）	29.97（7.17，82.30）	26.54（6.57，73.95）	-0.41(-0.48，-0.34）
Republic of Korea	751.01（651.71，856.59）	739.81（647.70，836.31）	0.01(-0.05，0.06）	127.70（112.52，145.12）	124.63（109.44，140.89）	0.01(-0.04，0.06）	580.65（260.79，1256.03）	500.05（234.92，1004.38）	-0.36(-0.41，-0.31）	35.68（9.05，90.00）	29.02（7.87，69.71）	-0.53(-0.60，-0.46）
Republic of Moldova	633.94（547.62，724.01）	640.52（549.24，730.71）	0.03(0.01，0.05）	110.64（96.04，126.38）	111.97（97.12，128.21）	0.03(0.01，0.05）	387.64（183.52，834.96）	372.43（187.00，797.15）	-0.14(-0.17，-0.11）	20.76（4.93，57.70）	19.02（4.72，50.98）	-0.29(-0.32，-0.26）

Romania	638.60（547.75，734.41）	637.20（547.10，729.32）	-0.04(-0.05，-0.02）	111.56（96.96，128.05）	111.32（96.82，127.73）	-0.03(-0.05，-0.02）	382.93（182.26，838.65）	382.75（185.17，803.55）	-0.02(-0.03，-0.01）	20.17（4.82，55.77）	20.06（4.83，53.48）	-0.03(-0.04，-0.02）
Russian Federation	669.98（580.01，769.14）	661.42（573.18，758.61）	-0.08(-0.13，-0.03）	117.07（102.29，133.67）	115.89（101.23，131.98）	-0.07(-0.12，-0.03）	406.03（192.39，878.44）	397.87（188.83，852.89）	-0.08(-0.10，-0.06）	21.62（5.27，59.64）	21.05（5.19，58.31）	-0.09(-0.10，-0.08）
Rwanda	619.83（537.09，704.91）	616.11（532.05，699.77）	0.04(0.02，0.06）	107.03（93.96，122.04）	106.57（93.24，120.76）	0.04(0.02，0.06）	438.80（199.65，980.20）	492.89（213.52，1114.28）	0.41(0.39，0.43）	25.00（6.26，69.32）	29.40（7.10，78.95）	0.56(0.54，0.59）
Saint Kitts and Nevis	540.25（465.78，620.82）	534.03（461.67，613.08）	-0.05(-0.06，-0.04）	94.29（82.57，107.74）	93.38（80.85，107.47）	-0.04(-0.05，-0.03）	314.31（149.41，678.21）	307.67（147.14，668.04）	0.02(-0.02，0.06）	16.15（3.76，45.57）	15.52（3.73，42.23）	0.03(-0.03，0.10）
Saint Lucia	568.49（488.10，652.02）	551.55（473.03，632.04）	-0.13(-0.15，-0.10）	98.80（85.78，113.56）	95.98（82.99，109.74）	-0.12(-0.14，-0.09）	325.28（155.18，704.03）	318.21（152.98，674.90）	-0.14(-0.16，-0.11）	16.63（3.90，46.02）	16.40（3.92，45.00）	-0.14(-0.18，-0.11）
Saint Vincent and the Grenadines	583.48（499.61，667.35）	553.95（476.99，632.63）	-0.16(-0.17，-0.16）	101.60（88.04，115.94）	96.56（84.19，110.27）	-0.16(-0.17，-0.16）	343.72（163.72，733.64）	313.61（151.38，678.48）	-0.22(-0.27，-0.17）	17.79（4.27，48.01）	15.86（3.81，43.79）	-0.23(-0.31，-0.14）
Samoa	643.90（547.03，740.91）	622.74（531.09，716.31）	-0.13(-0.14，-0.12）	111.48（96.61，128.63）	108.37（93.13，125.19）	-0.11(-0.12，-0.10）	479.55（214.02，1073.98）	457.23（202.49，1060.53）	-0.15(-0.19，-0.12）	27.74（6.81，74.15）	26.49（6.49，74.06）	-0.15(-0.21，-0.09）
San Marino	704.99（610.50，806.61）	623.02（535.54，718.97）	-0.41(-0.43，-0.39）	124.29（108.62，141.54）	110.90（96.02，127.71）	-0.38(-0.40，-0.36）	426.84（208.93，870.41）	384.39（193.81，772.22）	-0.28(-0.32，-0.24）	23.65（6.08，60.98）	21.49（5.71，51.92）	-0.20(-0.27，-0.13）
Sao Tome and Principe	442.32（381.71，502.56）	410.94（351.44，470.85）	-0.23(-0.25，-0.21）	78.52（67.94，90.02）	73.40（63.08，84.79）	-0.21(-0.23，-0.19）	311.00（139.50，704.54）	305.55（136.27，683.23）	-0.05(-0.09，0.00）	17.81（4.11，49.62）	17.68（4.41，47.61）	-0.01(-0.07，0.05）
Saudi Arabia	747.42（639.76，857.11）	701.74（600.97，807.91）	-0.20(-0.22，-0.19）	127.34（111.13，146.35）	120.44（104.42，138.45）	-0.19(-0.20，-0.17）	501.05（232.40，1064.97）	456.71（208.96，1019.77）	-0.33(-0.35，-0.31）	28.48（6.96，74.02）	25.85（6.36，71.39）	-0.36(-0.39，-0.33）
Senegal	462.03（395.41，527.14）	429.79（370.45，490.06）	-0.26(-0.27，-0.25）	82.21（71.27，94.43）	76.64（66.53，88.26）	-0.26(-0.27，-0.24）	330.44（147.37，740.71）	349.47（149.30，822.30）	0.17(0.14，0.19）	18.91（4.71，50.97）	20.86（4.67，57.98）	0.30(0.27，0.32）

Serbia	636.43（544.83，728.62）	638.51（545.56，731.10）	-0.01(-0.02，0.01）	111.13（96.63，127.15）	111.65（96.91，128.06）	0.00(-0.01，0.01）	383.30（178.08，837.65）	378.95（180.18，812.59）	-0.10(-0.13，-0.07）	20.31（4.77，57.47）	19.91（4.90，52.96）	-0.15(-0.19，-0.12）
Seychelles	667.93（571.70，769.28）	643.49（544.72，741.31）	-0.14(-0.14，-0.13）	113.14（98.39，129.40）	109.25（94.47，126.60）	-0.13(-0.14，-0.13）	417.52（195.33，908.01）	390.79（185.16，847.26）	-0.20(-0.22，-0.18）	22.01（5.49，61.18）	20.76（4.98，56.32）	-0.15(-0.18，-0.12）
Sierra Leone	469.47（403.35，535.67）	442.53（380.65，506.69）	-0.19(-0.20，-0.18）	83.78（72.77，95.51）	79.15（68.54，91.02）	-0.18(-0.19，-0.17）	304.50（140.12，675.61）	303.05（134.33，690.10）	0.00(-0.02，0.02）	16.94（3.86，46.86）	17.08（4.10，47.04）	0.02(0.00，0.05）
Singapore	516.08（443.63，583.07）	534.49（474.45，589.82）	0.11(0.07，0.15）	91.55（80.57，102.50）	94.88（85.54，104.85）	0.15(0.12，0.17）	334.82（160.51，718.72）	327.69（160.06，667.67）	-0.01(-0.03，0.02）	18.80（4.79，49.20）	17.90（4.76，44.10）	-0.08(-0.11，-0.04）
Slovakia	646.50（555.59，735.34）	635.02（547.19，728.34）	-0.06(-0.07，-0.05）	112.67（98.27，128.86）	110.87（97.08，127.00）	-0.05(-0.06，-0.04）	392.50（186.93，840.91）	386.66（186.37，808.10）	-0.02(-0.04，0.00）	20.91（5.16，57.44）	20.67（5.22，54.33）	0.02(-0.02，0.05）
Slovenia	634.22（546.70，725.16）	635.78（543.67，726.69）	0.05(0.02，0.08）	110.10（96.03，125.51）	110.51（96.63，126.67）	0.05(0.02，0.08）	382.00（178.71，834.83）	385.66（186.57，793.44）	0.05(0.00，0.10）	20.17（4.97，53.70）	20.80（5.25，51.88）	0.12(0.05，0.18）
Solomon Islands	654.61（557.57，751.56）	652.57（555.17，753.61）	0.03(0.01，0.06）	113.58（98.83，130.20）	113.74（98.07，131.08）	0.05(0.02，0.07）	431.35（195.74，993.79）	431.44（198.77，981.36）	0.02(-0.02，0.06）	23.45（5.50，69.31）	23.63（5.50，67.30）	0.02(-0.01，0.04）
Somalia	623.18（537.91，706.76）	617.03（535.75，699.67）	-0.01(-0.02，0.00）	107.79（94.20，122.85）	106.72（93.34，121.51）	-0.02(-0.02，-0.01）	415.69（191.62，905.33）	444.96（198.60，1030.23）	0.34(0.29，0.38）	22.73（5.37，62.88）	24.59（5.68，70.98）	0.44(0.38，0.51）
South Africa	646.32（559.39，738.74）	609.51（526.77，695.77）	-0.16(-0.17，-0.15）	114.18（99.52，130.09）	107.78（93.63，122.59）	-0.16(-0.17，-0.14）	396.83（186.63，850.32）	406.65（185.95，901.85）	0.07(0.01，0.13）	21.16（5.19，57.34）	22.58（5.40，61.80）	0.18(0.09，0.26）
South Sudan	616.16（531.63，702.53）	572.38（489.94，650.54）	-0.23(-0.26，-0.20）	105.62（92.12，120.57）	99.16（85.85，113.38）	-0.19(-0.21，-0.16）	411.64（184.29，891.43）	413.22（178.87，939.24）	0.00(-0.04，0.05）	23.10（5.34，61.24）	23.56（5.50，63.58）	0.05(0.01，0.09）
Spain	676.37（589.33，747.58）	582.15（504.96，664.35）	-0.37(-0.44，-0.30）	117.06（103.03，129.59）	102.50（88.99，116.45）	-0.37(-0.44，-0.29）	460.46（211.09，964.80）	418.19（195.24，860.08）	-0.25(-0.28，-0.22）	26.55（6.65，68.37）	24.86（6.56，61.21）	-0.17(-0.19，-0.14）

Sri Lanka	658.88（567.42，750.44）	634.13（545.74，727.67）	-0.13(-0.15，-0.12）	111.29（96.77，127.29）	107.46（93.30，123.54）	-0.13(-0.14，-0.11）	374.41（178.08，808.50）	369.00（177.21，764.88）	-0.04(-0.06，-0.02）	18.87（4.43，51.85）	18.92（4.40，49.86）	0.03(-0.01，0.07）
Sudan	790.96（686.08，900.75）	732.33（633.47，834.54）	-0.26(-0.27，-0.25）	134.77（118.25，154.07）	125.81（110.08，143.27）	-0.23(-0.24，-0.22）	478.34（226.03，1088.16）	442.22（209.16，934.95）	-0.28(-0.30，-0.26）	25.22（6.00，70.09）	23.36（5.68，62.70）	-0.27(-0.29，-0.24）
Suriname	602.96（519.13，689.29）	574.18（495.68，655.29）	-0.17(-0.19，-0.16）	105.02（91.54，120.14）	100.19（87.41，115.47）	-0.16(-0.18，-0.15）	333.79（166.14，711.38）	323.39（161.32，680.94）	-0.11(-0.13，-0.08）	16.58（4.06，44.78）	16.30（4.12，43.02）	-0.04(-0.08，0.00）
Sweden	755.36（656.33，856.34）	697.20（600.37，799.05）	-0.18(-0.22，-0.13）	137.72（121.98，154.24）	126.32（109.55，144.06）	-0.24(-0.27，-0.20）	447.24（215.79，921.22）	416.99（202.33，857.28）	-0.16(-0.18，-0.13）	24.65（6.34，63.75）	23.20（6.05，59.29）	-0.12(-0.18，-0.06）
Switzerland	715.63（614.44，820.18）	639.88（549.35，735.13）	-0.39(-0.42，-0.37）	124.61（108.66，142.14）	113.27（97.88，129.61）	-0.34(-0.36，-0.31）	449.64（212.92，955.62）	425.52（205.38，877.70）	-0.19(-0.26，-0.12）	24.88（6.22，64.30）	24.63（6.67，60.24）	-0.04(-0.14，0.06）
Syrian Arab Republic	819.88（707.35，932.52）	753.82（648.01，860.72）	-0.28(-0.29，-0.27）	139.41（122.36，159.02）	129.77（113.50，147.95）	-0.24(-0.25，-0.23）	482.32（235.56，1046.32）	451.67（219.33，983.43）	-0.23(-0.25，-0.21）	25.32（6.15，69.13）	23.83（5.62，66.40）	-0.21(-0.24，-0.18）
Taiwan (Province of China)	507.64（438.25，581.48）	555.31（472.14，624.76）	0.46(0.35，0.57）	91.21（78.72，104.55）	98.10（83.69，110.20）	0.38(0.28，0.47）	350.75（161.94，766.05）	372.14（175.26，790.04）	0.00(-0.11，0.10）	20.20（5.01，53.73）	21.13（5.44，53.08）	-0.24(-0.40，-0.08）
Tajikistan	638.14（552.15，725.33）	597.35（515.42，684.84）	-0.23(-0.24，-0.22）	111.93（98.19，127.63）	104.98（92.03，120.40）	-0.23(-0.23，-0.22）	392.87（186.09，849.45）	391.81（182.51，858.86）	-0.03(-0.05，0.00）	20.91（5.16，57.22）	21.59（5.44，58.06）	0.08(0.04，0.12）
Thailand	601.34（522.75，679.65）	603.22（522.58，689.60）	0.15(0.07，0.22）	102.51（89.15，116.40）	103.27（90.64，117.58）	0.12(0.06，0.17）	405.10（188.11，872.98）	395.44（185.94，837.14）	-0.15(-0.19，-0.12）	22.17（5.37，58.87）	21.11（5.47，54.92）	-0.34(-0.41，-0.28）
Timor-Leste	711.62（616.21，810.55）	664.45（572.75，759.71）	-0.21(-0.23，-0.18）	120.37（105.37，137.35）	113.20（98.46，129.74）	-0.18(-0.21，-0.16）	382.17（185.81，835.81）	399.65（185.73，906.77）	0.20(0.16，0.24）	18.75（4.45，53.28）	20.93（5.00，59.12）	0.42(0.38，0.46）
Togo	454.23（390.67，518.47）	438.21（378.97，499.37）	-0.10(-0.11，-0.09）	81.07（70.07，92.89）	78.26（67.73，90.00）	-0.09(-0.10，-0.09）	324.67（143.62，721.44）	340.84（148.60，819.20）	0.17(0.14，0.20）	18.55（4.34，50.51）	20.09（4.63，57.30）	0.26(0.22，0.30）

Tokelau	640.34（542.80，734.01）	626.80（531.25，725.34）	-0.07(-0.08，-0.06）	111.36（95.94，128.64）	109.37（94.11，126.60）	-0.06(-0.07，-0.05）	491.49（217.21，1100.53）	446.50（201.71，992.48）	-0.33(-0.33，-0.32）	28.32（6.98，78.23）	25.39（6.23，67.04）	-0.38(-0.39，-0.37）
Tonga	665.84（572.95，764.80）	644.39（551.18，740.15）	-0.12(-0.13，-0.10）	115.12（99.63，133.62）	111.80（97.12，128.75）	-0.11(-0.12，-0.09）	466.65（214.52，1043.66）	451.66（205.62，990.56）	-0.07(-0.09，-0.06）	26.42（6.50，71.51）	25.89（6.42，67.51）	-0.01(-0.04，0.01）
Trinidad and Tobago	578.04（497.45，659.10）	564.63（486.16，644.68）	-0.06(-0.07，-0.06）	100.82（87.88，115.33）	98.60（85.73，112.51）	-0.06(-0.07，-0.05）	326.90（158.27，713.26）	314.81（154.46，665.73）	-0.07(-0.09，-0.05）	16.66（3.98，45.80）	15.74（3.87，42.35）	-0.10(-0.14，-0.07）
Tunisia	846.86（730.77，961.13）	791.09（685.56，901.07）	-0.21(-0.22，-0.20）	142.94（125.86，162.19）	134.32（117.99，153.04）	-0.19(-0.20，-0.18）	527.40（245.11，1131.51）	485.94（234.03，1019.04）	-0.27(-0.28，-0.26）	28.25（6.90，75.36）	26.02（6.42，67.84）	-0.28(-0.30，-0.26）
Turkey	873.29（753.94，992.75）	819.36（706.20，936.59）	-0.25(-0.27，-0.23）	147.22（129.54，166.99）	139.10（120.57，158.16）	-0.22(-0.24，-0.20）	551.48（267.29，1158.43）	491.99（236.53，1031.11）	-0.40(-0.47，-0.32）	30.08（7.54，79.27）	26.16（6.61，69.03）	-0.45(-0.59，-0.32）
Turkmenistan	641.51（553.07，728.88）	609.86（523.20，698.24）	-0.18(-0.21，-0.15）	112.55（98.74，128.50）	107.05（93.39，122.99）	-0.18(-0.21，-0.16）	394.54（187.86，847.99）	373.23（181.83，824.24）	-0.26(-0.29，-0.23）	21.00（5.19，56.74）	19.71（4.78，54.98）	-0.32(-0.37，-0.26）
Tuvalu	668.63（572.05，767.56）	650.70（560.53，748.55）	-0.09(-0.10，-0.07）	116.09（100.33，134.46）	113.41（97.97，130.03）	-0.07(-0.08，-0.06）	479.06（219.38，1112.09）	456.66（210.11，1054.29）	-0.17(-0.18，-0.17）	26.98（6.56，77.12）	25.84（6.14，72.71）	-0.18(-0.19，-0.16）
Uganda	601.84（519.22，682.95）	594.04（508.15，676.31）	-0.05(-0.07，-0.02）	104.09（91.02，118.56）	102.68（89.05，117.37）	-0.05(-0.07，-0.02）	394.33（180.61，852.94）	453.76（201.58，1038.69）	0.49(0.45，0.52）	22.03（5.53，58.86）	26.87（6.75，71.15）	0.70(0.65，0.74）
Ukraine	672.20（576.91，781.63）	651.18（564.01，749.50）	-0.12(-0.17，-0.08）	119.03（103.58，135.93）	115.57（100.64，132.68）	-0.11(-0.15，-0.07）	402.57（191.76，867.85）	396.30（181.53，863.58）	-0.06(-0.08，-0.04）	21.50（5.16，60.47）	21.01（5.07，57.17）	-0.09(-0.11，-0.08）
United Arab Emirates	723.88（612.89，834.43）	652.43（552.16，753.80）	-0.33(-0.37，-0.29）	123.53（107.13，142.63）	113.49（98.05，131.08）	-0.26(-0.29，-0.23）	507.16（231.84，1108.06）	427.48（198.64，928.24）	-0.42(-0.52，-0.33）	28.84（7.07，74.40）	24.18（6.18，63.07）	-0.37(-0.51，-0.24）
United Kingdom	625.60（539.95，712.30）	591.08（508.13，674.77）	-0.13(-0.18，-0.09）	114.58（99.74，130.77）	107.93（93.37，123.89）	-0.17(-0.20，-0.13）	409.43（191.44，870.23）	390.40（183.98，817.67）	-0.10(-0.13，-0.08）	23.69（5.96，62.48）	22.61（5.88，57.62）	-0.09(-0.13，-0.05）

United Republic of Tanzania	620.11（533.93，703.93）	569.90（503.41，636.97）	-0.25(-0.27，-0.24）	107.04（92.95，122.58）	98.48（87.78，110.14）	-0.25(-0.26，-0.23）	448.87（200.94，973.23）	441.03（193.01，993.62）	-0.06(-0.08，-0.04）	25.72（6.30，68.21）	25.84（6.63，68.55）	0.00(-0.03，0.03）
United States of America	809.27（700.87，926.57）	773.07（670.26，887.66）	-0.17(-0.19，-0.16）	138.61（120.85，157.59）	131.29（113.93，149.60）	-0.19(-0.20，-0.17）	528.79（248.85，1122.13）	509.74（240.09，1068.07）	-0.15(-0.16，-0.13）	29.64（7.72，76.08）	29.17（7.74，73.52）	-0.08(-0.09，-0.06）
United States Virgin Islands	560.83（481.51，642.06）	543.82（470.45，627.55）	-0.11(-0.12，-0.10）	97.53（84.95，111.49）	94.57（82.02，109.17）	-0.11(-0.12，-0.11）	337.77（159.15，712.10）	302.56（148.84，624.93）	-0.34(-0.35，-0.33）	17.44（4.33，47.57）	15.20（3.67，40.13）	-0.40(-0.42，-0.38）
Uruguay	628.76（541.01，717.19）	595.24（515.90，680.56）	-0.23(-0.25，-0.20）	112.68（98.67，128.38）	107.09（93.07，121.78）	-0.21(-0.24，-0.19）	381.12（181.52，811.18）	379.78（180.21，797.09）	-0.03(-0.03，-0.02）	20.70（5.04，56.48）	21.17（5.41，54.56）	0.08(0.06，0.09）
Uzbekistan	608.52（524.64，693.37）	605.59（519.15，691.73）	-0.02(-0.04，0.00）	106.71（92.50，122.44）	106.19（92.22，122.08）	-0.02(-0.04，-0.01）	378.52（178.10，809.14）	367.07（176.44，801.55）	-0.09(-0.10，-0.08）	20.28（4.95，54.41）	19.32（4.83，54.55）	-0.15(-0.16，-0.13）
Vanuatu	617.37（527.17，714.38）	604.27（513.73，697.46）	-0.09(-0.11，-0.07）	107.32（92.84，124.06）	105.30（90.64，121.61）	-0.08(-0.10，-0.06）	414.90（186.63，961.38）	414.82（185.48，949.40）	-0.04(-0.06，-0.02）	22.86（5.23，64.50）	23.09（5.33，64.74）	-0.01(-0.03，0.01）
Venezuela (Bolivarian Republic of)	732.83（636.65，830.94）	717.86（620.45，816.18）	-0.08(-0.13，-0.04）	127.09（110.71，143.85）	124.37（108.40，141.62）	-0.08(-0.12，-0.05）	415.88（202.04，869.58）	409.15（203.98，846.15）	-0.06(-0.08，-0.05）	21.20（5.32，55.75）	20.72（5.12，52.50）	-0.06(-0.08，-0.03）
Viet Nam	689.39（596.14，787.87）	649.42（556.89，744.39）	-0.23(-0.24，-0.22）	116.54（102.61，133.79）	110.09（96.21，126.57）	-0.22(-0.23，-0.21）	454.56（211.42，984.40）	476.41（214.33，1068.35）	0.13(0.10，0.17）	24.62（5.91，65.66）	27.29（6.67，75.83）	0.31(0.27，0.36）
Yemen	856.69（745.98，974.32）	777.94（673.66，883.49）	-0.32(-0.35，-0.30）	144.23（127.32，163.56）	133.11（117.14，151.66）	-0.27(-0.29，-0.24）	521.37（246.81，1115.43）	486.21（234.59，1103.25）	-0.25(-0.27，-0.22）	27.41（6.57，74.87）	26.09（6.21，74.58）	-0.17(-0.19，-0.15）
Zambia	589.24（508.96，670.23）	591.53（514.14，674.45）	0.06(0.04，0.08）	102.35（89.13，116.62）	102.65（89.99，117.22）	0.06(0.04，0.08）	396.43（182.19，890.11）	431.45（196.36，999.22）	0.35(0.30，0.39）	21.96（5.30，62.38）	24.58（5.92，67.83）	0.46(0.40，0.52）

Zimbabwe	611.51（525.80，699.36）	584.43（506.45，665.47）	-0.14(-0.15，-0.13）	106.36（93.06，121.67）	102.44（89.41，116.93）	-0.11(-0.12，-0.10）	434.92（201.17，966.47）	417.09（191.94，965.99）	-0.11(-0.18，-0.05）	24.81（6.39，65.56）	23.11（5.38，63.68）	-0.16(-0.25，-0.06）

ASPR: Age-standardized prevalence rate; ASIR: Age-standardized incidence rate; ASYR: Age-standardized disability-adjusted life-years rate; ASDR: Age-standardized death rate;

UI: uncertainty intervals; CI: confidence interval; EAPC: estimated annual percentage change;

Supplementary table 3. The alzheimer's disease and other dementias burden by sex in global and different SDI regions from 1990 to 2021

Location	Female						Male					
	Number×10³ (95% UI)		EAPC of number (95% CI)	ASR (per 100,000 population) (95% UI)		EAPC of ASR (95% CI)	Number×10³ (95% UI)		EAPC of number (95% CI)	ASR (per 100,000 population) (95% UI)		EAPC of ASR (95% CI)
	1990	2021		1990	2021		1990	2021		1990	2021	
**Prevalence**												
Global	14143.45(12361.84,16105.29)	36103.38(31468.18,41117.47)	3.00（2.96，3.05）	736.15（646.05，834.27）	769.94（670.71，877.57）	0.04（0.01，0.06）	7656.32(6611.28,8728.10)	20753.31(17769.42,23796.80)	3.26（3.21，3.32）	571.47（497.63，654.31）	589.47（507.48，678.79）	0.03（0.01，0.05）
High SDI	5555.31(4870.46,6289.14)	11181.38(9802.82,12647.33)	2.33（2.31，2.34）	779.53（688.19，876.30）	785.95（688.16，890.84）	0.04（0.03，0.06）	2538.67(2204.76,2907.91)	6034.81(5187.53,6941.63)	2.97（2.89，3.05）	626.08（545.76，714.16）	605.22（522.16，693.98）	-0.08（-0.11，-0.05）
High-middle SDI	3836.78(3329.02,4397.74)	9789.84(8480.72,11201.32)	2.95（2.88，3.02）	737.93（643.63，839.80）	843.16（733.40，964.07）	0.27（0.22，0.31）	1843.79(1578.60,2120.90)	5135.26(4355.31,5916.71)	3.32（3.27，3.36）	589.50（510.81，677.40）	649.37（553.21，750.99）	0.20（0.17，0.23）
Middle SDI	2948.72(2552.22,3371.90)	10470.72(9083.47,12003.63)	3.97（3.88，4.05）	725.07（631.65，825.97）	809.65（701.25，927.83）	0.11（0.05，0.17）	1882.90(1616.75,2148.96)	6331.53(5391.99,7261.25)	3.88（3.82，3.94）	553.15（480.49，635.90）	608.64（520.50，703.76）	0.15（0.12，0.19）
Low-middle SDI	1313.97(1137.74,1489.92)	3524.33(3056.14,4010.45)	3.27（3.24，3.30）	602.52（527.44，682.93）	568.49（493.61，645.46）	-0.24（-0.26，-0.22）	1036.26(895.43,1184.83)	2485.05(2142.54,2829.99)	2.83（2.79，2.86）	479.39（417.72，546.49）	470.74（408.92，538.63）	-0.14（-0.17，-0.12）
Low SDI	472.47(405.64,535.45)	1105.90(957.44,1250.78)	2.82（2.77，2.87）	619.99（539.69，701.01）	587.31（511.26，666.01）	-0.20（-0.21，-0.19）	345.68(296.59,394.17)	749.10(644.73,852.84)	2.51（2.46，2.56）	453.10（394.41，514.95）	430.26（374.07，490.13）	-0.23（-0.25，-0.21）
**Incidence**												

Global	2482.21(2183.97,2820.92)	6191.56(5432.75,7009.23)	2.93（2.89，2.97）	127.82（112.82，144.18）	132.29（116.30，149.80）	0.00（-0.03，0.03）	1352.32(1177.68,1551.79)	3645.49(3144.74,4183.54)	3.25（3.20，3.30）	100.69（88.05，114.43）	103.40（89.45，118.45）	0.02（0.00，0.03）
High SDI	978.95(867.43,1105.65)	1892.16(1676.95,2140.30)	2.18（2.16，2.19）	136.53（121.26，152.80）	135.14（119.13，152.48）	-0.03（-0.04，-0.01）	456.47(396.16,521.51)	1059.99(914.02,1211.00)	2.89（2.81，2.96）	111.51（97.94，126.03）	106.34（92.24，121.37）	-0.12（-0.14，-0.11）
High-middle SDI	667.32(579.59,765.01)	1678.92(1469.52,1904.99)	2.91（2.85，2.97）	127.20（111.61，143.80）	145.37（127.62，165.13）	0.27（0.23，0.32）	323.30(278.56,373.47)	903.43(771.15,1042.12)	3.33（3.29，3.37）	103.15（89.78，117.91）	114.05（97.80，131.27）	0.21（0.18，0.24）
Middle SDI	517.09(450.78,589.03)	1795.52(1574.35,2041.90)	3.91（3.83，3.99）	125.88（110.73，143.03）	137.75（120.93，156.84）	0.07（0.02，0.12）	327.42(283.73,374.38)	1106.56(949.94,1274.80)	3.90（3.84，3.95）	96.86（84.21，110.85）	106.23（91.67，122.15）	0.15（0.11，0.18）
Low-middle SDI	232.18(203.07,263.24)	623.19(546.50,706.85)	3.28（3.24，3.31）	105.75（92.30，119.89）	100.27（87.57，114.21）	-0.23（-0.25，-0.21）	183.21(159.78,208.75)	440.08(384.92,501.59)	2.83（2.80，2.87）	84.93（73.77，97.27）	83.35（72.46，95.38）	-0.14（-0.17，-0.12）
Low SDI	83.84(73.22,94.95)	196.36(171.79,222.48)	2.82（2.77，2.87）	109.03（95.07，123.92）	103.42（90.00，117.04）	-0.20（-0.21，-0.19）	60.33(52.35,68.61)	132.35(115.52,150.72)	2.55（2.51，2.60）	80.31（69.79，92.07）	76.65（66.62，87.44）	-0.21（-0.23，-0.19）
**DALYs**												
Global	9106.75(4330.35,19615.10)	23808.56(11368.14,49746.52)	3.12（3.07，3.17）	495.05（231.43，1054.27）	504.87（241.04，1055.02）	0.00（-0.01，0.02）	4465.56(2092.52,9971.77)	12524.13(5871.76,27158.68)	3.40（3.33，3.48）	362.99（164.92，799.27）	372.53（170.89，805.03）	0.05（0.03，0.07）
High SDI	3687.80(1750.59,7789.84)	7903.18(3802.46,15828.73)	2.50（2.48，2.52）	517.49（245.57，1089.20）	512.73（249.67，1037.58）	-0.04（-0.05，-0.03）	1490.52(702.68,3285.78)	3828.80(1754.29,8207.68)	3.19（3.11，3.28）	393.36（180.72，854.70）	383.32（175.81，818.46）	-0.08（-0.10，-0.06）
High-middle SDI	2455.09(1149.17,5310.72)	6209.41(2990.66,13030.89)	2.99（2.93，3.06）	498.74（230.18，1065.12）	530.35（255.11，1113.12）	0.12（0.10，0.14）	1095.30(511.54,2484.75)	3034.36(1422.31,6692.58)	3.33（3.27，3.40）	386.01（174.11，859.76）	402.84（184.37，884.29）	0.07（0.05，0.09）
Middle SDI	1869.93(886.79,4086.77)	6465.46(3117.57,13738.27)	3.99（3.90，4.07）	491.87（229.28，1064.42）	512.74（244.54，1088.44）	-0.01（-0.04，0.03）	1105.71(514.20,2482.36)	3675.70(1756.56,8024.60)	3.90（3.82，3.97）	355.01（160.38，787.81）	376.17（174.32，825.87）	0.09（0.06，0.11）

Low-middle SDI	787.55(371.69,1738.33)	2421.83(1085.17,5320.29)	3.75（3.72，3.78）	382.96（180.33，833.50）	406.42（183.27，888.16）	0.17（0.16，0.19）	570.27(270.43,1283.77)	1503.44(686.85,3310.28)	3.21（3.18，3.23）	284.02（132.66，632.89）	303.52（137.60，662.39）	0.22（0.21，0.23）
Low SDI	296.86(137.32,657.65)	789.03(351.08,1772.49)	3.29（3.21，3.37）	423.11（194.04，918.42）	454.65（201.89，1019.44）	0.24（0.20，0.29）	198.85(91.93,449.03)	471.58(211.06,1098.18)	2.89（2.82，2.97）	286.16（131.28，653.22）	298.27（130.91，693.97）	0.16（0.11，0.22）
**Deaths**												
Global	462.91(115.21,1215.53)	1325.80(356.48,3316.45)	3.45（3.40，3.50）	27.60（6.96，71.87）	27.88（7.48，69.79）	-0.01（-0.02，0.01）	200.38(47.76,550.52)	626.87(153.87,1677.85)	3.80（3.72，3.88）	20.18（4.98，55.70）	20.71（5.19，55.50）	0.07（0.05，0.09）
High SDI	208.26(53.58,535.73)	502.40(141.79,1192.46)	2.87（2.84，2.90）	29.16（7.58，74.16）	28.83（7.93，68.94）	-0.05（-0.06，-0.04）	73.64(17.77,203.09)	216.94(54.89,577.00)	3.65（3.55，3.74）	22.34（5.59，60.40）	21.72（5.52，57.51）	-0.09（-0.12，-0.07）
High-middle SDI	122.15(30.18,328.58)	340.63(91.13,878.05)	3.36（3.28，3.44）	27.77（6.85，73.87）	28.70（7.68，73.94）	0.06（0.04，0.09）	48.56(11.53,134.01)	149.47(36.89,404.09)	3.73（3.64，3.83）	21.68（5.26，59.77）	22.18（5.58，59.95）	0.02（0.00，0.04）
Middle SDI	85.01(20.41,222.95)	324.06(82.25,842.12)	4.37（4.27，4.46）	26.73（6.51，70.81）	27.36（7.07，70.95）	-0.02（-0.05，0.00）	45.83(10.79,127.05)	169.36(41.42,458.73)	4.29（4.20，4.38）	19.16（4.60，53.53）	20.31（5.02，54.96）	0.11（0.09，0.14）
Low-middle SDI	34.29(8.04,94.13)	119.91(29.33,319.54)	4.22（4.19，4.26）	20.17（4.83，55.71）	22.54（5.53，60.25）	0.37（0.35，0.40）	24.13(5.66,68.19)	69.68(16.59,186.52)	3.56（3.52，3.59）	15.01（3.67，41.80）	16.69（4.01，44.88）	0.41（0.38，0.43）
Low SDI	12.73(3.08,34.50)	37.73(9.21,105.27)	3.72（3.64，3.79）	23.03（5.68，63.28）	26.28（6.44，72.40）	0.46（0.39，0.54）	8.00(1.89,22.98)	20.90(4.81,58.92)	3.30（3.24，3.36）	15.42（3.68，43.98）	16.82（3.96，46.92）	0.37（0.29，0.45）

SDI: socio-demographic index; DALYs: disability-adjusted life-years; UI: uncertainty intervals; CI: confidence intervals; EAPC: estimated annual percentage change; ASR: age-standardized rate;

Supplementary table 4. The change of three risk factors contributed to alzheimer's disease and other dementias burden by sex in global from 1990 to 2021

Measure	Number×10³ (95% UI)		EAPC of number (95% CI)	PAF (per 100,000) (95% UI)		EAPC of ASR (95% CI)	ASR (per 100,000 population) (95% UI)		EAPC of ASR (95% CI)
	1990	2021		1990	2021		1990	2021	
**High fasting plasma glucose**									
DALYs	1441.54(82.70,3878.46)	5348.85(308.06,14351.16)	4.43(4.38,4.48)	10.50(0.89,21.20)	14.64(1.21,29.44)	1.19(1.10,1.29)	47.07(2.72,126.46)	66.42(3.83,178.85)	1.18(1.09,1.26)
Deaths	71.47(2.85,221.70)	290.03(11.76,916.71)	4.76(4.70,4.82)	10.48(0.89,21.03)	14.70(1.21,29.41)	1.22(1.12,1.32)	2.64(0.11,8.38)	3.73(0.15,11.84)	1.20(1.10,1.29)
**Female**									
DALYs	943.70(54.23,2541.83)	3419.72(198.32,9146.43)	4.32(4.27,4.37)	10.21(0.87,20.58)	14.28(1.17,28.63)	1.20(1.10,1.29)	50.81(2.93,136.84)	72.55(4.21,193.70)	1.20(1.11,1.29)
Deaths	48.40(1.95,148.60)	191.13(7.85,600.48)	4.64(4.58,4.70)	10.19(0.86,20.40)	14.32(1.17,28.78)	1.22(1.12,1.31)	2.83(0.11,8.93)	4.03(0.17,12.61)	1.21(1.11,1.31)
**Male**									
DALYs	497.84(28.47,1368.86)	1929.13(109.74,5194.92)	4.62(4.57,4.67)	11.22(0.96,22.70)	15.41(1.28,31.18)	1.16(1.06,1.25)	40.93(2.36,112.07)	57.71(3.29,155.61)	1.21(1.12,1.29)
Deaths	23.07(0.90,74.59)	98.90(3.91,316.13)	5.00(4.94,5.06)	11.31(0.97,22.72)	15.63(1.29,31.59)	1.18(1.07,1.28)	2.30(0.09,7.47)	3.26(0.13,10.51)	1.25(1.16,1.33)
**High body-mass index**									
DALYs	644.75(-42.11,2422.44)	2665.75(-494.34,9332.49)	4.66(4.60,4.72)	4.61(-0.44,13.50)	7.07(-1.71,19.86)	1.37(1.36,1.38)	21.39(-1.39,80.20)	32.86(-5.97,115.18)	1.32(1.30,1.35)
Deaths	31.58(-1.62,142.81)	139.44(-21.84,580.29)	4.88(4.82,4.94)	4.59(-0.41,13.46)	6.80(-1.52,19.23)	1.23(1.21,1.25)	1.22(-0.07,5.43)	1.79(-0.27,7.44)	1.17(1.15,1.20)
**Female**									
DALYs	493.77(-47.61,1827.37)	1881.16(-377.25,6557.38)	4.34(4.27,4.41)	5.24(-0.70,15.24)	7.68(-2.06,21.38)	1.20(1.17,1.22)	26.91(-2.52,99.27)	39.91(-8.02,138.91)	1.18(1.14,1.21)
Deaths	24.82(-1.87,109.38)	100.59(-16.80,413.61)	4.53(4.47,4.60)	5.13(-0.63,15.04)	7.25(-1.76,20.42)	1.04(1.01,1.07)	1.50(-0.11,6.53)	2.12(-0.35,8.69)	1.01(0.97,1.05)

**Male**									
DALYs	150.98(-3.04,614.70)	784.58(-117.10,2808.64)	5.55(5.51,5.60)	3.21(-0.08,9.46)	6.01(-1.12,17.15)	2.14(2.10,2.19)	12.33(-0.23,50.00)	23.18(-3.28,82.98)	2.12(2.09,2.14)
Deaths	6.76(-0.09,32.94)	38.85(-4.83,165.65)	5.94(5.88,6.00)	3.18(-0.06,9.41)	5.91(-1.03,16.96)	2.14(2.08,2.19)	0.70(-0.01,3.41)	1.29(-0.15,5.45)	2.09(2.06,2.12)
**Smoking**									
DALYs	794.92(344.38,1839.71)	1533.21(662.72,3496.42)	2.08(1.98,2.18)	5.25(3.69,6.80)	4.08(2.85,5.31)	-0.86(-0.90,-0.82)	23.33(9.99,54.46)	18.36(7.90,42.07)	-0.88(-0.92,-0.83)
Deaths	32.16(7.45,89.32)	67.18(15.69,184.66)	2.33(2.25,2.42)	4.32(3.02,5.64)	3.31(2.30,4.34)	-0.95(-0.98,-0.91)	1.08(0.25,3.03)	0.84(0.19,2.29)	-0.95(-0.99,-0.91)
**Female**									
DALYs	262.31(112.86,602.15)	423.19(185.08,947.54)	1.41(1.37,1.46)	2.77(1.92,3.64)	1.79(1.24,2.36)	-1.50(-1.55,-1.46)	13.65(5.80,31.32)	9.01(3.94,20.16)	-1.50(-1.56,-1.45)
Deaths	11.66(2.79,31.82)	20.42(4.95,54.00)	1.65(1.59,1.71)	2.43(1.67,3.25)	1.55(1.06,2.07)	-1.59(-1.67,-1.52)	0.67(0.16,1.83)	0.43(0.10,1.14)	-1.60(-1.68,-1.52)
**Male**									
DALYs	532.61(227.76,1241.83)	1110.02(474.04,2558.50)	2.38(2.25,2.51)	10.36(7.35,13.37)	8.25(5.80,10.68)	-0.78(-0.83,-0.72)	37.45(15.98,87.69)	30.56(12.72,71.50)	-0.73(-0.80,-0.66)
Deaths	20.50(4.68,57.87)	46.76(11.06,132.71)	2.69(2.56,2.82)	8.85(6.31,11.42)	6.92(4.87,9.12)	-0.87(-0.91,-0.82)	1.78(0.40,5.14)	1.43(0.34,4.05)	-0.79(-0.86,-0.73)

DALYs: disability-adjusted life-years; UI: uncertainty intervals; CI: confidence intervals; EAPC: estimated annual percentage change; ASR: age-standardized rate; PAF: population attributable fraction;

Supplementary table 5. The change of high fasting plasma glucose contributed to alzheimer's disease and other dementias burden by sex at 21 GBD regions from 1990 to 2021

Location	Number×10^3 (95% UI)		EAPC of number (95% CI)	PAF (%) (95% UI)		EAPC of ASR (95% CI)	ASR (per 100,000 population) (95% UI)		EAPC of ASR (95% CI)
	1990	2021		1990	2021		1990	2021	
**DALYs**									
Andean Latin America	3.92(0.22,10.50)	21.94(1.25,59.87)	6.12(5.96,6.28)	8.30(0.69,17.07)	14.51(1.13,30.32)	1.96(1.85,2.07)	23.22(1.30,61.77)	39.74(2.27,108.36)	1.85(1.74,1.96)
Australasia	9.18(0.59,24.35)	33.12(2.07,86.66)	4.23(4.13,4.32)	9.25(0.83,18.27)	13.10(1.21,24.50)	1.21(1.17,1.26)	41.29(2.61,109.78)	53.45(3.37,140.67)	0.90(0.85,0.95)
Caribbean	10.33(0.59,28.01)	30.09(1.73,79.96)	3.42(3.37,3.47)	14.18(1.11,28.85)	17.28(1.40,35.03)	0.62(0.60,0.65)	45.81(2.58,124.65)	54.44(3.15,143.84)	0.50(0.47,0.52)
Central Asia	10.00(0.56,27.03)	30.98(1.63,90.21)	4.03(3.81,4.26)	6.68(0.54,13.86)	13.35(0.99,28.17)	2.60(2.45,2.75)	26.09(1.43,71.86)	50.89(2.64,148.85)	2.50(2.35,2.64)
Central Europe	56.07(3.41,149.74)	160.79(9.66,419.38)	3.69(3.60,3.77)	11.38(0.97,22.38)	16.99(1.42,33.62)	1.34(1.29,1.38)	44.82(2.72,120.49)	65.91(3.96,171.31)	1.29(1.23,1.34)
Central Latin America	32.49(1.96,85.55)	137.13(8.08,365.35)	4.76(4.72,4.80)	14.41(1.16,29.37)	17.51(1.38,36.13)	0.50(0.47,0.53)	49.62(2.94,130.32)	59.09(3.48,158.37)	0.44(0.41,0.47)
Central Sub-Saharan Africa	6.81(0.39,17.81)	24.29(1.30,67.88)	4.15(4.10,4.21)	11.37(0.98,22.21)	13.63(1.11,27.65)	0.54(0.51,0.58)	61.21(3.49,163.56)	80.90(4.25,226.58)	0.90(0.84,0.97)
East Asia	294.75(16.20,827.60)	1246.62(73.20,3299.33)	4.83(4.68,4.98)	10.59(0.92,21.34)	11.89(1.01,23.77)	0.62(0.44,0.79)	56.22(3.10,154.24)	66.37(3.89,176.69)	0.59(0.43,0.75)
Eastern Europe	63.68(3.63,177.12)	156.20(8.87,425.39)	3.19(3.02,3.35)	6.73(0.53,13.69)	10.74(0.88,21.64)	1.71(1.64,1.79)	27.35(1.55,75.53)	42.84(2.44,117.18)	1.64(1.57,1.72)
Eastern Sub-Saharan Africa	13.92(0.70,39.35)	45.67(2.15,132.68)	3.99(3.86,4.12)	7.92(0.60,17.28)	9.46(0.70,20.32)	0.58(0.56,0.61)	34.28(1.72,96.43)	43.80(2.07,126.35)	0.83(0.79,0.86)
High-income Asia Pacific	106.88(6.75,271.52)	451.64(28.33,1154.34)	4.79(4.61,4.97)	13.33(1.21,25.48)	14.69(1.30,27.80)	0.19(0.10,0.29)	63.12(3.95,161.32)	68.17(4.29,174.66)	0.20(0.12,0.29)
High-income North America	213.37(13.11,557.95)	731.69(43.23,1958.23)	4.26(4.10,4.42)	10.89(1.01,20.81)	19.83(1.66,39.37)	2.22(2.03,2.41)	57.20(3.52,148.96)	99.65(5.91,264.59)	2.03(1.84,2.23)
North Africa and Middle East	66.26(3.76,180.36)	300.18(17.51,793.11)	5.29(5.21,5.38)	11.45(0.93,23.39)	19.13(1.57,38.59)	1.82(1.76,1.87)	59.36(3.36,161.27)	91.57(5.33,245.55)	1.54(1.48,1.59)

Oceania	1.03(0.06,2.92)	3.30(0.19,8.94)	3.75(3.69,3.81)	15.66(1.26,32.40)	18.95(1.53,38.17)	0.64(0.62,0.67)	67.24(3.80,189.18)	75.67(4.18,205.90)	0.35(0.33,0.37)
South Asia	115.26(6.30,304.14)	569.30(30.29,1590.85)	5.35(5.31,5.39)	11.74(0.97,24.39)	16.49(1.35,33.39)	1.07(1.03,1.10)	32.12(1.76,87.08)	51.08(2.72,142.69)	1.48(1.45,1.51)
Southeast Asia	74.19(4.02,202.01)	309.89(16.18,870.21)	4.69(4.64,4.75)	11.04(0.86,23.40)	15.25(1.14,32.48)	1.09(1.03,1.15)	44.42(2.34,122.98)	64.21(3.31,182.73)	1.21(1.12,1.30)
Southern Latin America	15.03(0.91,40.44)	53.99(3.17,142.99)	4.18(4.06,4.29)	9.77(0.82,19.65)	15.76(1.30,31.67)	1.53(1.49,1.56)	37.22(2.24,100.30)	58.30(3.42,154.79)	1.44(1.39,1.48)
Southern Sub-Saharan Africa	9.36(0.46,26.86)	26.57(1.37,76.34)	3.58(3.49,3.68)	11.66(0.87,25.75)	15.65(1.14,33.32)	1.17(1.09,1.25)	47.16(2.29,136.25)	64.33(3.31,187.21)	1.22(1.10,1.33)
Tropical Latin America	41.89(2.31,117.13)	185.36(10.59,498.43)	5.12(5.05,5.18)	11.99(0.97,25.05)	15.06(1.25,30.28)	0.90(0.85,0.95)	61.55(3.44,169.07)	76.27(4.36,204.86)	0.85(0.81,0.89)
Western Europe	282.17(16.23,751.57)	779.50(43.78,2116.19)	3.40(3.37,3.43)	10.03(0.82,20.43)	14.32(1.13,29.22)	1.16(1.10,1.22)	46.35(2.67,124.41)	63.81(3.60,173.67)	1.04(0.98,1.11)
Western Sub-Saharan Africa	14.95(0.80,41.31)	50.61(2.52,144.78)	4.13(4.06,4.21)	8.40(0.66,17.72)	12.44(0.94,26.23)	1.33(1.28,1.37)	26.33(1.40,73.22)	40.06(1.99,116.54)	1.46(1.40,1.53)
**Female**									
High SDI	386.97(23.17,1016.46)	1230.59(72.61,3285.99)	3.90(3.81,3.99)	10.30(0.90,20.23)	15.54(1.28,30.74)	1.42(1.31,1.52)	53.60(3.21,140.55)	80.12(4.74,211.61)	1.38(1.27,1.48)
middle SDI	244.67(14.11,668.08)	820.05(49.11,2165.51)	4.07(3.99,4.16)	9.79(0.82,19.68)	13.10(1.09,26.04)	1.08(0.95,1.20)	49.14(2.83,134.02)	69.86(4.18,183.97)	1.20(1.07,1.32)
Middle SDI	204.87(11.10,562.05)	893.60(51.31,2418.27)	4.91(4.85,4.97)	10.92(0.90,22.68)	13.70(1.12,27.97)	0.88(0.80,0.96)	54.00(2.92,149.19)	70.76(4.06,191.57)	0.88(0.80,0.95)
middle SDI	80.50(4.36,215.73)	376.65(20.23,1017.03)	5.20(5.17,5.23)	10.39(0.84,21.37)	15.52(1.25,31.68)	1.33(1.29,1.37)	39.95(2.17,106.25)	63.39(3.41,173.52)	1.52(1.48,1.55)
Low SDI	25.57(1.36,70.06)	95.63(4.86,269.31)	4.45(4.39,4.52)	8.77(0.71,18.10)	12.01(0.95,24.82)	1.04(1.01,1.08)	37.29(1.99,101.63)	54.86(2.79,153.91)	1.29(1.27,1.32)
Andean Latin America	2.25(0.12,6.00)	13.02(0.72,35.09)	1.94(1.82,2.06)	8.18(0.67,17.03)	14.34(1.11,29.33)	1.85(1.72,1.98)	25.05(1.38,66.93)	43.23(2.40,116.38)	6.17(6.01,6.32)
Australasia	6.23(0.40,16.59)	21.06(1.29,54.30)	1.27(1.21,1.33)	8.71(0.78,17.34)	12.57(1.17,23.41)	1.03(0.96,1.09)	44.49(2.85,119.13)	59.86(3.72,154.70)	3.97(3.85,4.10)
Caribbean	5.85(0.34,15.77)	17.89(1.04,46.89)	0.61(0.57,0.64)	14.00(1.09,28.33)	17.03(1.39,34.51)	0.48(0.44,0.51)	48.49(2.76,129.91)	57.45(3.35,149.57)	3.55(3.49,3.62)

Central Asia	7.18(0.39,19.57)	21.48(1.13,62.37)	2.71(2.53,2.89)	6.69(0.54,13.94)	13.65(1.01,29.03)	2.62(2.44,2.80)	27.90(1.52,77.35)	55.68(2.91,161.89)	3.89(3.65,4.13)
Central Europe	38.20(2.34,102.01)	109.87(6.70,284.73)	1.29(1.25,1.33)	11.36(0.97,22.08)	16.76(1.41,33.02)	1.26(1.21,1.30)	48.23(2.94,128.87)	70.34(4.28,181.65)	3.68(3.60,3.77)
Central Latin America	19.52(1.17,51.60)	85.53(5.09,231.51)	0.45(0.41,0.50)	14.73(1.18,29.66)	17.69(1.41,35.98)	0.37(0.33,0.42)	56.49(3.34,149.38)	65.71(3.91,177.85)	4.88(4.83,4.92)
Central Sub-Saharan Africa	3.90(0.22,10.27)	15.81(0.85,44.02)	0.72(0.67,0.77)	9.78(0.86,19.40)	12.40(1.01,25.23)	0.94(0.85,1.03)	65.03(3.69,172.44)	86.93(4.60,247.34)	4.56(4.52,4.60)
East Asia	190.25(10.48,526.08)	802.24(47.99,2135.07)	0.59(0.42,0.76)	10.57(0.91,21.29)	11.84(1.00,23.62)	0.58(0.41,0.74)	62.71(3.46,172.65)	74.37(4.44,197.36)	4.77(4.63,4.92)
Eastern Europe	49.09(2.83,134.83)	116.49(6.70,318.01)	1.72(1.64,1.80)	6.65(0.52,13.45)	10.73(0.88,21.51)	1.70(1.61,1.78)	28.42(1.62,78.80)	45.67(2.63,124.21)	3.03(2.87,3.19)
Eastern Sub-Saharan Africa	8.04(0.41,22.39)	27.56(1.32,79.07)	0.62(0.59,0.66)	7.08(0.55,15.17)	8.47(0.64,17.98)	0.84(0.79,0.89)	37.07(1.88,102.02)	47.06(2.25,134.59)	4.20(4.07,4.34)
High-income Asia Pacific	74.32(4.73,189.77)	301.99(19.02,761.07)	0.03(-0.07,0.14)	13.01(1.18,24.93)	13.76(1.23,26.53)	0.06(-0.03,0.16)	69.30(4.38,177.91)	72.16(4.60,177.29)	4.62(4.40,4.84)
High-income North America	138.86(8.56,359.95)	457.41(26.59,1236.33)	2.30(2.13,2.47)	10.21(0.95,19.43)	19.07(1.55,38.30)	2.20(2.01,2.39)	57.83(3.55,150.40)	105.85(6.19,281.91)	4.10(3.94,4.26)
North Africa and Middle East	37.68(2.13,102.67)	169.95(10.08,444.76)	1.83(1.78,1.88)	11.35(0.92,23.32)	19.17(1.58,38.41)	1.57(1.50,1.63)	65.13(3.69,178.58)	102.00(6.04,269.18)	5.24(5.16,5.32)
Oceania	0.54(0.03,1.52)	1.85(0.11,4.84)	0.89(0.84,0.93)	14.44(1.17,29.74)	18.75(1.51,38.12)	0.57(0.53,0.61)	70.74(3.99,195.12)	84.77(4.74,226.57)	3.93(3.83,4.03)
South Asia	60.96(3.22,164.70)	338.23(17.79,936.75)	1.14(1.09,1.20)	11.32(0.93,23.63)	16.23(1.32,32.99)	1.53(1.49,1.56)	35.11(1.87,95.86)	56.71(2.99,158.50)	5.80(5.76,5.83)
Southeast Asia	47.76(2.55,128.70)	202.54(10.54,566.57)	1.11(1.05,1.17)	10.96(0.84,23.59)	15.09(1.13,32.17)	1.22(1.13,1.31)	49.86(2.60,135.69)	71.84(3.69,203.46)	4.79(4.73,4.86)
Southern Latin America	10.39(0.63,28.58)	36.87(2.21,95.31)	1.41(1.37,1.46)	9.82(0.82,19.74)	15.47(1.30,30.47)	1.33(1.28,1.37)	42.41(2.59,116.58)	64.72(3.87,167.47)	4.11(3.98,4.24)
Southern Sub-Saharan Africa	6.87(0.33,19.47)	19.87(1.03,57.28)	1.19(1.11,1.27)	11.82(0.88,26.15)	15.95(1.16,33.94)	1.19(1.09,1.30)	55.22(2.66,157.08)	74.44(3.86,216.84)	3.64(3.52,3.75)

Tropical Latin America	26.10(1.42,71.41)	115.96(6.67,310.25)	0.78(0.72,0.84)	12.12(0.97,25.46)	14.77(1.23,29.32)	0.68(0.64,0.73)	68.14(3.77,188.72)	81.01(4.66,216.80)	5.09(5.03,5.15)
Western Europe	201.11(11.55,539.55)	513.25(28.85,1387.40)	1.12(1.07,1.18)	9.85(0.81,20.15)	13.94(1.10,28.71)	1.06(0.99,1.12)	49.74(2.86,133.14)	68.94(3.90,186.86)	3.09(3.05,3.13)
Western Sub-Saharan Africa	8.60(0.47,23.63)	30.85(1.56,88.88)	1.56(1.51,1.62)	7.61(0.61,15.66)	11.88(0.91,24.84)	1.82(1.73,1.91)	27.51(1.49,75.44)	45.54(2.29,134.49)	4.35(4.25,4.46)
**Male**									
High SDI	178.78(10.65,477.45)	680.39(40.37,1802.56)	4.62(4.54,4.70)	11.79(1.04,23.49)	17.57(1.48,34.55)	1.41(1.27,1.55)	46.63(2.79,124.98)	67.74(4.02,180.00)	1.33(1.21,1.44)
middle SDI	113.14(6.47,315.16)	417.55(24.02,1111.70)	4.51(4.41,4.61)	10.37(0.89,20.73)	13.73(1.16,27.50)	1.10(0.96,1.23)	40.27(2.32,109.65)	55.55(3.18,147.43)	1.16(1.04,1.29)
Middle SDI	123.05(6.72,341.32)	517.51(28.85,1401.85)	4.84(4.77,4.91)	11.47(0.96,23.64)	14.12(1.15,29.04)	0.81(0.73,0.89)	40.93(2.25,114.66)	53.36(2.96,143.44)	0.89(0.82,0.97)
middle SDI	62.16(3.53,166.52)	245.47(13.24,699.71)	4.60(4.57,4.63)	11.07(0.92,22.80)	16.36(1.32,33.81)	1.30(1.27,1.33)	31.62(1.78,85.73)	49.93(2.69,142.12)	1.52(1.49,1.55)
Low SDI	20.11(1.11,55.19)	66.46(3.38,186.82)	3.96(3.89,4.02)	10.89(0.88,22.62)	14.46(1.12,30.67)	0.88(0.86,0.90)	31.37(1.72,87.68)	43.27(2.20,124.00)	1.04(0.98,1.09)
Andean Latin America	1.67(0.10,4.61)	8.91(0.53,24.10)	1.99(1.90,2.09)	8.46(0.70,17.34)	14.80(1.17,31.00)	1.85(1.75,1.94)	21.10(1.20,57.44)	35.54(2.09,95.95)	6.05(5.89,6.22)
Australasia	2.95(0.19,8.04)	12.07(0.78,32.93)	0.98(0.95,1.01)	10.74(0.97,20.42)	14.19(1.29,26.75)	0.88(0.86,0.91)	35.30(2.15,96.37)	45.17(2.93,123.65)	4.72(4.68,4.76)
Caribbean	4.49(0.25,12.49)	12.20(0.70,33.07)	0.66(0.64,0.67)	14.42(1.13,29.95)	17.66(1.41,35.66)	0.51(0.50,0.53)	42.61(2.36,120.32)	50.63(2.88,137.19)	3.24(3.20,3.28)
Central Asia	2.82(0.16,7.69)	9.50(0.50,27.22)	2.35(2.26,2.43)	6.64(0.56,13.61)	12.67(0.94,26.41)	2.32(2.24,2.41)	22.23(1.25,60.58)	42.23(2.16,124.02)	4.38(4.17,4.60)
Central Europe	17.87(1.09,48.27)	50.92(2.99,132.85)	1.43(1.36,1.50)	11.34(0.97,22.41)	17.37(1.44,34.71)	1.37(1.29,1.44)	38.48(2.31,102.62)	57.90(3.38,153.04)	3.69(3.59,3.79)
Central Latin America	12.97(0.79,34.82)	51.60(2.99,141.55)	0.58(0.54,0.63)	13.88(1.14,28.45)	17.24(1.35,35.95)	0.47(0.42,0.52)	41.90(2.49,110.86)	50.53(2.93,136.82)	4.57(4.50,4.63)
Central Sub-Saharan Africa	2.91(0.17,7.89)	8.48(0.46,23.86)	0.57(0.54,0.59)	14.59(1.24,28.61)	17.37(1.45,35.60)	0.75(0.71,0.79)	56.98(3.29,156.24)	71.13(3.76,204.65)	3.51(3.39,3.62)
East Asia	104.49(5.73,299.51)	444.38(25.85,1205.69)	0.65(0.47,0.83)	10.67(0.96,21.72)	12.00(1.04,24.32)	0.72(0.56,0.88)	45.45(2.51,127.37)	54.86(3.10,146.57)	4.93(4.78,5.09)

Eastern Europe	14.60(0.82,40.51)	39.70(2.17,110.49)	1.65(1.54,1.77)	7.16(0.56,14.79)	10.86(0.87,22.36)	1.61(1.50,1.73)	24.43(1.35,67.31)	36.63(2.01,101.64)	3.69(3.50,3.88)
Eastern Sub-Saharan Africa	5.88(0.30,16.83)	18.10(0.84,53.46)	0.63(0.61,0.65)	9.64(0.71,21.51)	11.79(0.84,25.69)	0.76(0.74,0.78)	31.38(1.54,93.99)	39.75(1.83,116.86)	3.68(3.55,3.82)
High-income Asia Pacific	32.56(2.00,84.56)	149.65(9.31,391.42)	0.45(0.37,0.54)	14.21(1.31,27.22)	16.70(1.46,32.01)	0.54(0.48,0.61)	51.58(3.14,135.92)	61.00(3.79,159.78)	5.16(5.05,5.27)
High-income North America	74.51(4.57,194.37)	274.28(16.65,739.82)	1.97(1.74,2.21)	12.52(1.15,23.95)	21.26(1.87,41.21)	1.79(1.58,2.00)	56.64(3.48,149.28)	91.76(5.57,249.41)	4.56(4.40,4.71)
North Africa and Middle East	28.58(1.64,77.67)	130.23(7.42,348.35)	1.78(1.72,1.85)	11.63(0.96,23.46)	19.08(1.54,38.63)	1.51(1.45,1.56)	53.31(3.02,145.94)	80.79(4.59,219.95)	5.36(5.26,5.46)
Oceania	0.49(0.03,1.38)	1.45(0.08,4.04)	0.30(0.30,0.31)	17.58(1.41,36.73)	19.25(1.56,39.18)	0.10(0.08,0.13)	63.54(3.59,183.53)	66.29(3.62,190.11)	3.54(3.51,3.57)
South Asia	54.30(3.10,145.18)	231.07(12.69,656.45)	0.99(0.95,1.03)	12.25(1.03,25.20)	16.86(1.38,34.41)	1.30(1.24,1.36)	29.24(1.65,79.84)	44.30(2.38,126.81)	4.79(4.71,4.86)
Southeast Asia	26.44(1.47,71.50)	107.35(5.62,302.80)	1.05(0.98,1.12)	11.24(0.90,23.75)	15.59(1.16,33.04)	1.14(1.04,1.24)	36.94(2.00,102.86)	53.10(2.77,153.30)	4.50(4.44,4.57)
Southern Latin America	4.64(0.27,12.47)	17.12(0.96,49.18)	1.79(1.74,1.84)	9.63(0.81,19.32)	16.47(1.30,33.33)	1.71(1.65,1.77)	28.75(1.69,77.34)	48.02(2.71,137.05)	4.32(4.23,4.41)
Southern Sub-Saharan Africa	2.49(0.13,7.20)	6.70(0.34,19.48)	1.14(1.05,1.22)	11.21(0.83,24.97)	14.85(1.09,31.81)	1.16(1.06,1.26)	33.25(1.66,98.49)	44.57(2.24,133.23)	3.43(3.38,3.47)
Tropical Latin America	15.78(0.89,44.35)	69.41(3.92,189.21)	1.12(1.06,1.18)	11.81(0.97,24.48)	15.65(1.28,31.73)	1.11(1.05,1.16)	52.70(3.00,146.59)	69.60(3.93,191.59)	5.15(5.07,5.23)
Western Europe	81.06(4.68,221.83)	266.25(14.87,733.58)	1.20(1.13,1.28)	10.48(0.86,21.60)	15.10(1.19,31.38)	1.18(1.11,1.25)	39.22(2.27,107.61)	55.85(3.11,153.39)	4.08(4.03,4.12)
Western Sub-Saharan Africa	6.34(0.33,18.03)	19.76(0.96,57.65)	0.95(0.90,1.00)	10.03(0.78,21.80)	13.57(1.01,29.06)	0.97(0.92,1.02)	25.12(1.29,70.94)	33.76(1.63,100.58)	3.83(3.76,3.90)
**Deaths**									
Andean Latin America	0.19(0.01,0.64)	1.12(0.05,3.66)	6.35(6.19,6.51)	8.28(0.67,16.93)	14.53(1.11,30.24)	1.97(1.86,2.09)	1.21(0.05,3.99)	2.07(0.08,6.73)	1.85(1.73,1.97)
Australasia	0.47(0.02,1.49)	1.95(0.08,5.95)	4.74(4.61,4.87)	9.13(0.81,18.00)	12.80(1.18,24.00)	1.17(1.13,1.22)	2.26(0.10,7.24)	3.00(0.13,9.17)	1.00(0.95,1.05)

Caribbean	0.48(0.02,1.58)	1.58(0.06,5.16)	3.84(3.75,3.93)	14.20(1.12,29.14)	17.39(1.40,35.41)	0.63(0.61,0.65)	2.34(0.09,7.89)	2.77(0.11,9.07)	0.52(0.50,0.55)
Central Asia	0.49(0.02,1.63)	1.48(0.05,4.92)	3.89(3.62,4.17)	6.69(0.55,13.86)	13.20(0.97,27.92)	2.56(2.40,2.72)	1.39(0.05,4.67)	2.67(0.09,9.01)	2.44(2.28,2.59)
Central Europe	2.63(0.10,8.45)	8.63(0.35,27.52)	4.16(4.03,4.29)	11.45(0.97,22.42)	17.09(1.42,33.76)	1.33(1.29,1.38)	2.39(0.09,7.83)	3.52(0.14,11.21)	1.29(1.24,1.34)
Central Latin America	1.41(0.05,4.59)	6.65(0.26,21.54)	5.11(5.04,5.17)	13.86(1.11,28.37)	17.13(1.34,35.54)	0.53(0.50,0.57)	2.40(0.09,7.84)	2.91(0.11,9.44)	0.47(0.42,0.51)
Central Sub-Saharan Africa	0.29(0.01,0.89)	1.12(0.04,3.57)	4.57(4.54,4.60)	11.51(0.99,22.70)	13.39(1.10,26.94)	0.42(0.38,0.46)	3.53(0.15,11.07)	4.70(0.18,15.26)	0.93(0.85,1.01)
East Asia	13.42(0.53,44.75)	61.21(2.43,189.56)	5.15(5.00,5.31)	10.63(0.92,21.48)	11.79(1.00,23.66)	0.58(0.40,0.76)	3.31(0.13,10.45)	3.62(0.14,11.46)	0.39(0.24,0.55)
Eastern Europe	2.97(0.11,9.58)	8.18(0.31,26.02)	3.58(3.30,3.86)	6.72(0.52,13.80)	10.72(0.86,21.63)	1.72(1.64,1.80)	1.46(0.05,4.81)	2.27(0.08,7.25)	1.62(1.55,1.70)
Eastern Sub-Saharan Africa	0.66(0.02,2.19)	2.28(0.08,7.47)	4.19(4.08,4.30)	8.28(0.61,18.02)	9.38(0.69,20.16)	0.41(0.40,0.43)	2.02(0.07,6.75)	2.57(0.09,8.60)	0.83(0.81,0.86)
High-income Asia Pacific	5.81(0.26,17.46)	29.85(1.41,88.27)	5.41(5.25,5.57)	13.47(1.22,25.98)	14.93(1.31,28.45)	0.22(0.15,0.30)	3.78(0.17,11.46)	4.00(0.19,11.88)	0.13(0.07,0.19)
High-income North America	11.46(0.50,35.03)	43.13(1.76,137.44)	4.63(4.43,4.83)	10.58(0.98,20.12)	19.87(1.63,39.72)	2.35(2.14,2.55)	3.09(0.14,9.47)	5.66(0.23,18.07)	2.23(2.02,2.44)
North Africa and Middle East	3.07(0.12,9.85)	14.26(0.57,45.46)	5.50(5.38,5.62)	11.34(0.91,23.07)	18.97(1.54,38.12)	1.82(1.77,1.87)	3.20(0.13,10.32)	4.89(0.19,15.67)	1.51(1.44,1.58)
Oceania	0.04(0.00,0.14)	0.14(0.01,0.46)	4.05(3.96,4.15)	15.60(1.24,32.08)	18.91(1.51,38.55)	0.65(0.64,0.67)	3.60(0.14,12.10)	4.01(0.15,13.44)	0.32(0.30,0.33)
South Asia	5.03(0.18,16.40)	27.99(1.03,90.78)	5.82(5.77,5.87)	12.01(0.98,25.08)	16.59(1.34,34.03)	1.02(0.99,1.05)	1.71(0.06,5.56)	2.87(0.10,9.42)	1.74(1.70,1.77)
Southeast Asia	3.45(0.13,11.28)	15.38(0.57,51.42)	4.91(4.83,4.99)	11.54(0.88,24.51)	15.68(1.17,33.53)	1.05(0.98,1.12)	2.40(0.09,7.87)	3.58(0.13,11.99)	1.30(1.18,1.41)
Southern Latin America	0.73(0.03,2.41)	2.95(0.12,9.43)	4.58(4.42,4.74)	9.59(0.80,19.18)	15.59(1.28,31.11)	1.55(1.51,1.59)	1.99(0.08,6.51)	3.15(0.12,10.08)	1.49(1.45,1.53)
Southern Sub-Saharan Africa	0.47(0.02,1.57)	1.27(0.05,4.34)	3.36(3.27,3.46)	12.10(0.88,26.73)	15.56(1.12,33.10)	1.00(0.94,1.06)	2.64(0.09,8.79)	3.56(0.12,12.35)	1.14(1.02,1.25)

Tropical Latin America	1.95(0.08,6.27)	9.63(0.40,29.68)	5.54(5.45,5.63)	11.70(0.94,24.23)	14.66(1.21,29.19)	0.89(0.84,0.94)	3.31(0.13,10.48)	4.04(0.17,12.44)	0.83(0.78,0.89)
Western Europe	15.71(0.62,49.39)	48.69(1.95,153.97)	3.76(3.73,3.80)	9.97(0.82,20.28)	14.28(1.12,29.06)	1.17(1.11,1.22)	2.69(0.11,8.61)	3.71(0.15,11.65)	1.06(1.00,1.13)
Western Sub-Saharan Africa	0.74(0.03,2.44)	2.55(0.09,8.56)	4.24(4.16,4.32)	8.56(0.66,18.20)	12.16(0.91,25.37)	1.18(1.14,1.21)	1.55(0.06,5.12)	2.36(0.08,8.03)	1.48(1.40,1.55)
**Female**									
High SDI	21.66(0.91,67.73)	77.38(3.26,239.14)	4.28(4.19,4.38)	10.21(0.89,19.97)	15.53(1.27,30.66)	1.45(1.35,1.56)	3.00(0.13,9.43)	4.51(0.19,13.97)	1.40(1.30,1.51)
middle SDI	12.37(0.49,38.77)	45.04(1.84,139.99)	4.40(4.30,4.49)	9.86(0.82,19.90)	13.10(1.08,26.09)	1.05(0.92,1.18)	2.76(0.11,8.76)	3.79(0.15,11.75)	1.12(0.98,1.27)
Middle SDI	9.52(0.36,30.33)	44.96(1.76,143.57)	5.24(5.17,5.30)	10.90(0.89,22.74)	13.63(1.11,27.85)	0.87(0.78,0.95)	2.94(0.11,9.36)	3.77(0.15,12.14)	0.85(0.78,0.93)
middle SDI	3.65(0.13,11.69)	18.93(0.70,60.64)	5.59(5.55,5.64)	10.52(0.85,21.74)	15.50(1.24,31.54)	1.29(1.25,1.34)	2.13(0.08,6.71)	3.52(0.13,11.44)	1.68(1.64,1.73)
Low SDI	1.14(0.04,3.72)	4.65(0.17,14.93)	4.80(4.73,4.87)	8.83(0.71,18.31)	11.79(0.92,24.20)	0.97(0.93,1.02)	2.05(0.08,6.53)	3.12(0.11,10.25)	1.45(1.40,1.50)
Andean Latin America	0.11(0.00,0.36)	0.67(0.03,2.17)	1.97(1.83,2.10)	8.09(0.66,16.95)	14.29(1.07,29.41)	1.85(1.71,1.99)	1.28(0.05,4.16)	2.20(0.08,7.18)	6.43(6.28,6.59)
Australasia	0.33(0.01,1.06)	1.28(0.06,3.84)	1.21(1.16,1.27)	8.68(0.77,17.12)	12.32(1.14,23.05)	1.14(1.08,1.21)	2.41(0.10,7.71)	3.34(0.15,10.05)	4.48(4.31,4.65)
Caribbean	0.27(0.01,0.88)	0.94(0.04,3.04)	0.61(0.57,0.65)	14.03(1.10,28.50)	17.12(1.40,34.79)	0.50(0.46,0.54)	2.42(0.09,8.02)	2.85(0.11,9.21)	4.03(3.93,4.13)
Central Asia	0.36(0.01,1.19)	1.06(0.04,3.58)	2.67(2.48,2.86)	6.70(0.54,13.95)	13.50(0.99,28.72)	2.54(2.35,2.74)	1.47(0.06,4.98)	2.88(0.10,9.81)	3.79(3.52,4.05)
Central Europe	1.83(0.07,5.91)	6.08(0.25,19.15)	1.29(1.26,1.33)	11.49(0.98,22.20)	16.96(1.42,33.22)	1.27(1.23,1.31)	2.55(0.10,8.30)	3.73(0.15,11.70)	4.17(4.04,4.30)
Central Latin America	0.84(0.03,2.71)	4.17(0.17,13.61)	0.50(0.45,0.55)	14.04(1.11,28.74)	17.25(1.36,35.33)	0.40(0.34,0.46)	2.70(0.10,8.78)	3.21(0.13,10.49)	5.27(5.20,5.34)
Central Sub-Saharan Africa	0.16(0.01,0.50)	0.75(0.03,2.39)	0.63(0.59,0.68)	9.84(0.87,19.40)	12.20(1.00,24.65)	0.99(0.88,1.09)	3.67(0.16,11.17)	4.98(0.20,16.29)	5.15(5.09,5.20)
East Asia	9.00(0.37,29.12)	40.63(1.60,128.45)	0.56(0.39,0.74)	10.60(0.90,21.41)	11.75(0.99,23.63)	0.37(0.21,0.54)	3.65(0.14,11.50)	3.97(0.16,12.65)	5.09(4.94,5.24)
Eastern Europe	2.34(0.09,7.56)	6.22(0.23,19.50)	1.72(1.63,1.80)	6.62(0.51,13.56)	10.66(0.86,21.34)	1.68(1.59,1.76)	1.49(0.06,4.91)	2.38(0.09,7.43)	3.39(3.13,3.66)

Eastern Sub-Saharan Africa	0.38(0.01,1.24)	1.39(0.05,4.52)	0.47(0.44,0.50)	7.26(0.55,15.48)	8.31(0.62,17.70)	0.87(0.82,0.92)	2.13(0.08,6.87)	2.73(0.10,9.11)	4.44(4.32,4.56)
High-income Asia Pacific	4.17(0.19,12.56)	20.84(1.02,61.13)	0.10(0.01,0.19)	13.16(1.18,25.09)	14.12(1.25,27.16)	0.01(-0.05,0.07)	4.14(0.19,12.37)	4.25(0.21,12.44)	5.29(5.09,5.50)
High-income North America	7.75(0.34,23.30)	27.61(1.12,89.45)	2.42(2.24,2.60)	9.97(0.93,18.93)	19.19(1.53,38.66)	2.36(2.17,2.56)	3.11(0.14,9.42)	5.92(0.24,18.67)	4.41(4.22,4.61)
North Africa and Middle East	1.75(0.07,5.63)	7.94(0.32,25.01)	1.83(1.78,1.88)	11.16(0.89,22.85)	18.88(1.55,37.92)	1.56(1.47,1.64)	3.43(0.14,10.95)	5.35(0.21,16.95)	5.39(5.28,5.50)
Oceania	0.02(0.00,0.07)	0.08(0.00,0.26)	0.88(0.85,0.90)	14.48(1.16,29.54)	18.69(1.50,37.84)	0.50(0.49,0.51)	3.74(0.14,12.55)	4.40(0.16,14.32)	4.21(4.09,4.34)
South Asia	2.68(0.09,8.70)	17.14(0.62,55.45)	1.09(1.03,1.15)	11.70(0.95,24.45)	16.44(1.33,33.64)	1.78(1.73,1.83)	1.87(0.06,6.14)	3.21(0.11,10.43)	6.33(6.27,6.39)
Southeast Asia	2.28(0.08,7.35)	10.29(0.38,34.05)	1.06(0.99,1.13)	11.44(0.86,24.36)	15.45(1.16,33.06)	1.30(1.19,1.42)	2.67(0.10,8.64)	3.98(0.15,13.22)	5.02(4.94,5.09)
Southern Latin America	0.52(0.02,1.71)	2.08(0.08,6.47)	1.43(1.39,1.48)	9.67(0.80,19.47)	15.30(1.28,29.88)	1.37(1.32,1.41)	2.27(0.09,7.41)	3.49(0.14,10.90)	4.52(4.35,4.70)
Southern Sub-Saharan Africa	0.36(0.01,1.17)	0.98(0.03,3.35)	1.01(0.95,1.07)	12.24(0.89,26.73)	15.80(1.14,33.56)	1.11(0.99,1.23)	3.05(0.11,10.12)	4.08(0.14,13.88)	3.45(3.34,3.56)
Tropical Latin America	1.25(0.05,3.94)	6.23(0.27,19.08)	0.77(0.70,0.83)	11.82(0.94,24.49)	14.34(1.20,28.38)	0.69(0.63,0.75)	3.65(0.14,11.44)	4.29(0.19,13.11)	5.55(5.47,5.64)
Western Europe	11.57(0.46,36.43)	33.19(1.35,103.50)	1.13(1.07,1.18)	9.85(0.81,20.14)	13.95(1.10,28.47)	1.08(1.01,1.14)	2.87(0.12,9.13)	3.99(0.16,12.41)	3.46(3.40,3.52)
Western Sub-Saharan Africa	0.43(0.02,1.39)	1.56(0.06,5.18)	1.43(1.38,1.48)	7.61(0.61,15.83)	11.45(0.87,23.92)	1.89(1.79,1.98)	1.58(0.06,5.14)	2.66(0.09,9.18)	4.44(4.35,4.53)
**Male**									
High SDI	8.89(0.37,28.34)	38.73(1.58,124.19)	5.09(5.00,5.17)	11.73(1.04,23.32)	17.65(1.48,34.71)	1.46(1.31,1.60)	2.64(0.11,8.64)	3.87(0.16,12.41)	1.36(1.24,1.49)
middle SDI	5.18(0.20,17.19)	20.92(0.85,67.95)	4.85(4.74,4.96)	10.48(0.91,20.78)	13.83(1.15,27.73)	1.07(0.94,1.21)	2.29(0.09,7.43)	3.09(0.12,9.93)	1.10(0.96,1.23)
Middle SDI	5.34(0.20,17.75)	24.38(0.95,78.10)	5.15(5.06,5.24)	11.62(0.97,24.09)	14.23(1.14,29.28)	0.78(0.70,0.87)	2.25(0.08,7.34)	2.91(0.11,9.26)	0.89(0.82,0.97)

middle SDI	2.75(0.10,8.91)	11.67(0.43,38.37)	4.90(4.85,4.95)	11.16(0.92,22.99)	16.38(1.31,33.67)	1.28(1.25,1.31)	1.69(0.06,5.51)	2.76(0.10,9.17)	1.69(1.65,1.73)
Low SDI	0.88(0.03,2.90)	3.11(0.11,10.14)	4.26(4.20,4.32)	11.35(0.90,23.73)	14.61(1.12,30.54)	0.79(0.77,0.81)	1.77(0.06,5.89)	2.47(0.09,8.20)	1.15(1.07,1.24)
Andean Latin America	0.08(0.00,0.27)	0.46(0.02,1.45)	2.00(1.91,2.10)	8.52(0.70,17.35)	14.94(1.16,31.23)	1.84(1.75,1.94)	1.13(0.04,3.75)	1.90(0.08,6.09)	6.25(6.07,6.42)
Australasia	0.14(0.01,0.45)	0.67(0.03,2.13)	0.93(0.91,0.96)	10.67(0.97,20.46)	13.93(1.28,26.31)	0.94(0.91,0.97)	1.94(0.08,6.32)	2.51(0.11,7.95)	5.33(5.27,5.39)
Caribbean	0.21(0.01,0.71)	0.64(0.02,2.07)	0.67(0.65,0.69)	14.43(1.14,30.07)	17.83(1.40,36.15)	0.55(0.53,0.57)	2.23(0.08,7.73)	2.67(0.10,8.65)	3.58(3.50,3.67)
Central Asia	0.13(0.01,0.44)	0.42(0.01,1.37)	2.26(2.18,2.35)	6.67(0.56,13.61)	12.47(0.92,25.83)	2.25(2.17,2.33)	1.20(0.05,3.96)	2.25(0.08,7.36)	4.19(3.88,4.51)
Central Europe	0.79(0.03,2.60)	2.55(0.10,8.30)	1.43(1.36,1.50)	11.34(0.96,22.36)	17.38(1.41,34.85)	1.37(1.30,1.43)	2.06(0.08,6.70)	3.11(0.12,10.29)	4.12(3.98,4.26)
Central Latin America	0.57(0.02,1.88)	2.47(0.10,8.16)	0.60(0.55,0.65)	13.50(1.11,27.63)	16.97(1.31,35.60)	0.49(0.43,0.54)	2.06(0.08,6.77)	2.52(0.10,8.27)	4.86(4.77,4.94)
Central Sub-Saharan Africa	0.12(0.00,0.41)	0.37(0.01,1.25)	0.47(0.46,0.49)	14.99(1.28,29.14)	17.42(1.46,35.82)	0.75(0.69,0.82)	3.37(0.13,11.00)	4.22(0.16,14.19)	3.63(3.55,3.72)
East Asia	4.42(0.17,14.70)	20.58(0.82,67.92)	0.60(0.41,0.78)	10.77(0.98,21.94)	11.92(1.02,24.08)	0.54(0.38,0.70)	2.68(0.10,8.65)	3.04(0.12,9.70)	5.27(5.09,5.44)
Eastern Europe	0.63(0.02,2.10)	1.96(0.07,6.54)	1.66(1.53,1.78)	7.26(0.57,14.91)	10.98(0.88,22.64)	1.59(1.46,1.72)	1.34(0.05,4.51)	1.99(0.08,6.70)	4.24(3.90,4.57)
Eastern Sub-Saharan Africa	0.28(0.01,0.94)	0.89(0.03,2.99)	0.46(0.45,0.47)	10.58(0.76,23.66)	12.20(0.85,26.71)	0.75(0.74,0.77)	1.88(0.06,6.71)	2.36(0.08,7.94)	3.84(3.73,3.95)
High-income Asia Pacific	1.64(0.07,5.20)	9.01(0.39,27.94)	0.45(0.38,0.52)	14.58(1.35,27.86)	17.09(1.48,32.38)	0.44(0.39,0.49)	3.03(0.13,9.41)	3.51(0.15,10.86)	5.71(5.65,5.76)
High-income North America	3.71(0.16,11.90)	15.52(0.66,50.36)	2.07(1.83,2.32)	12.31(1.13,23.58)	21.34(1.84,41.48)	2.00(1.76,2.24)	3.13(0.14,10.07)	5.34(0.23,17.34)	5.06(4.85,5.27)
North Africa and Middle East	1.32(0.05,4.30)	6.32(0.25,20.51)	1.78(1.71,1.84)	11.67(0.96,23.45)	19.06(1.53,38.76)	1.47(1.41,1.53)	2.96(0.11,9.67)	4.43(0.17,14.46)	5.64(5.50,5.77)
Oceania	0.02(0.00,0.06)	0.06(0.00,0.20)	0.31(0.31,0.32)	17.52(1.39,36.71)	19.26(1.54,38.93)	0.11(0.07,0.15)	3.43(0.13,11.64)	3.59(0.13,12.05)	3.86(3.80,3.92)
South Asia	2.35(0.08,7.84)	10.85(0.41,35.69)	0.94(0.90,0.99)	12.39(1.03,25.58)	16.83(1.36,34.15)	1.52(1.46,1.59)	1.55(0.05,5.02)	2.44(0.09,8.08)	5.14(5.06,5.21)

Southeast Asia	1.17(0.04,3.89)	5.09(0.19,16.92)	1.01(0.93,1.08)	11.89(0.94,25.30)	16.23(1.20,34.24)	1.22(1.11,1.34)	2.00(0.07,6.72)	2.96(0.11,10.03)	4.71(4.60,4.81)
Southern Latin America	0.21(0.01,0.70)	0.87(0.03,2.98)	1.85(1.79,1.90)	9.42(0.79,18.91)	16.39(1.28,33.40)	1.80(1.74,1.86)	1.49(0.06,4.87)	2.54(0.10,8.62)	4.74(4.60,4.87)
Southern Sub-Saharan Africa	0.12(0.00,0.40)	0.29(0.01,0.99)	0.96(0.90,1.03)	11.76(0.86,25.99)	14.93(1.08,31.74)	1.04(0.95,1.14)	1.85(0.06,6.27)	2.43(0.08,8.82)	3.11(3.05,3.16)
Tropical Latin America	0.70(0.03,2.30)	3.40(0.13,10.79)	1.13(1.07,1.19)	11.56(0.95,23.91)	15.39(1.25,31.22)	1.06(0.99,1.12)	2.82(0.11,9.19)	3.65(0.14,11.69)	5.51(5.39,5.63)
Western Europe	4.13(0.16,13.63)	15.50(0.59,50.80)	1.22(1.15,1.30)	10.41(0.85,21.31)	15.09(1.18,31.23)	1.22(1.15,1.29)	2.24(0.09,7.34)	3.21(0.12,10.54)	4.53(4.48,4.57)
Western Sub-Saharan Africa	0.31(0.01,1.04)	0.99(0.03,3.36)	0.79(0.74,0.83)	10.63(0.80,22.94)	13.70(1.00,29.17)	0.92(0.86,0.98)	1.52(0.05,5.18)	2.01(0.06,6.99)	3.97(3.88,4.05)

DALYs: disability-adjusted life-years; UI: uncertainty intervals; CI: confidence intervals; PAF: population attributable fraction; EAPC: estimated annual percentage change; GBD: Global Burden of Diseases; ASR: age-standardized rate;

Supplementary table 6. The change of high fasting plasma glucose contributed to alzheimer's disease and other dementias burden at 204 countries and territories from 1990 to 2021

Location	ASYR (per 100,000 population) (95% UI)		EAPC of ASYR (95% CI)	ASDR (per 100,000 population) (95% UI)		EAPC of ASDR (95% CI)
	1990	2021		1990	2021	
Afghanistan	82.25(4.02,237.27)	118.82(6.65,330.77)	1.21(1.18,1.24)	4.71(0.17,15.28)	6.80(0.29,22.10)	1.22(1.19,1.26)
Albania	32.34(1.96,85.08)	46.16(2.75,124.10)	1.19(1.13,1.25)	1.78(0.07,5.60)	2.49(0.10,8.09)	1.12(1.06,1.17)
Algeria	73.16(4.06,198.10)	99.90(6.04,266.86)	1.03(0.93,1.14)	4.03(0.15,13.14)	5.49(0.23,17.66)	1.06(0.94,1.19)
American Samoa	89.12(5.09,254.47)	91.55(6.05,242.35)	0.10(0.08,0.13)	4.92(0.20,16.48)	4.97(0.24,16.09)	0.08(0.03,0.13)
Andorra	39.98(2.43,113.20)	54.71(3.06,149.53)	1.05(0.98,1.12)	2.29(0.09,7.18)	3.14(0.12,9.97)	1.08(1.00,1.16)
Angola	52.49(2.51,143.76)	76.95(3.49,221.24)	1.23(1.20,1.27)	2.96(0.10,9.64)	4.44(0.14,14.73)	1.28(1.24,1.33)
Antigua and Barbuda	53.62(2.85,153.53)	58.95(3.29,163.39)	0.40(0.36,0.43)	2.73(0.10,9.43)	2.99(0.10,10.25)	0.43(0.38,0.48)
Argentina	39.14(2.38,105.32)	56.78(3.39,151.17)	1.14(1.08,1.20)	2.11(0.08,6.84)	3.09(0.12,9.81)	1.21(1.15,1.27)
Armenia	29.43(1.52,80.01)	51.52(2.66,144.89)	1.82(1.57,2.07)	1.49(0.05,4.86)	2.70(0.09,8.95)	1.98(1.73,2.23)
Australia	40.12(2.55,106.58)	51.95(3.24,137.74)	0.90(0.84,0.97)	2.19(0.09,6.93)	2.93(0.13,9.08)	1.03(0.96,1.10)
Austria	25.91(1.55,69.60)	37.83(2.32,102.60)	1.21(1.10,1.33)	1.49(0.06,4.77)	2.14(0.09,6.83)	1.19(1.09,1.29)
Azerbaijan	27.60(1.49,77.03)	52.52(2.75,153.41)	2.42(2.21,2.62)	1.46(0.05,4.87)	2.78(0.10,9.45)	2.46(2.21,2.71)
Bahrain	96.37(5.08,281.98)	101.62(6.42,269.07)	0.14(0.07,0.21)	5.32(0.19,17.88)	5.55(0.22,18.02)	0.08(-0.03,0.19)
Bangladesh	30.56(1.65,85.61)	48.26(2.51,132.17)	1.49(1.38,1.60)	1.69(0.06,5.78)	2.72(0.09,8.71)	1.51(1.34,1.69)
Barbados	60.20(3.65,159.61)	64.41(4.12,167.84)	0.19(0.14,0.24)	3.01(0.11,10.06)	3.27(0.15,10.42)	0.26(0.18,0.33)
Belarus	21.88(1.17,61.16)	30.61(1.69,84.02)	1.27(1.12,1.42)	1.14(0.04,3.87)	1.58(0.05,5.55)	1.25(1.08,1.42)

Belgium	43.45(2.55,119.52)	55.01(3.28,149.48)	0.73(0.71,0.75)	2.48(0.10,7.85)	3.17(0.14,10.10)	0.80(0.77,0.84)
Belize	42.76(2.31,120.52)	54.30(3.02,149.51)	0.89(0.77,1.01)	2.11(0.07,7.24)	2.73(0.10,9.14)	0.96(0.83,1.09)
Benin	25.11(1.27,70.65)	39.41(1.86,114.70)	1.56(1.47,1.65)	1.45(0.05,4.76)	2.25(0.07,7.79)	1.50(1.40,1.59)
Bermuda	39.41(2.44,106.78)	51.27(3.22,129.33)	0.85(0.78,0.91)	2.03(0.08,6.74)	2.62(0.12,8.08)	0.85(0.76,0.93)
Bhutan	28.91(1.58,80.53)	53.23(2.52,155.03)	2.14(2.08,2.20)	1.56(0.06,5.25)	3.14(0.11,10.74)	2.46(2.39,2.53)
Bolivia	29.19(1.46,88.17)	47.62(2.68,131.54)	1.59(1.45,1.72)	1.49(0.05,5.30)	2.51(0.10,8.30)	1.68(1.52,1.85)
Bosnia and Herzegovina	41.15(2.40,111.57)	73.69(4.45,203.76)	2.04(1.81,2.28)	2.17(0.09,7.08)	3.88(0.15,12.57)	2.06(1.87,2.25)
Botswana	44.82(1.99,135.85)	57.80(2.78,173.40)	1.00(0.90,1.11)	2.53(0.07,8.52)	3.12(0.11,10.92)	0.84(0.73,0.95)
Brazil	62.11(3.47,170.86)	76.30(4.36,204.99)	0.83(0.78,0.87)	3.34(0.13,10.59)	4.04(0.17,12.44)	0.81(0.75,0.86)
Brunei	68.71(3.77,191.04)	81.57(4.54,225.97)	0.63(0.56,0.70)	3.83(0.15,12.72)	4.81(0.19,16.00)	0.83(0.74,0.93)
Bulgaria	42.53(2.67,115.56)	64.49(3.68,183.56)	1.47(1.31,1.63)	2.17(0.08,7.26)	3.40(0.12,11.60)	1.57(1.40,1.75)
Burkina Faso	29.08(1.41,89.70)	40.25(1.91,123.19)	1.02(0.95,1.09)	1.79(0.06,6.26)	2.34(0.08,8.18)	0.79(0.72,0.86)
Burundi	31.69(1.57,94.24)	40.64(1.94,119.80)	0.85(0.83,0.88)	1.81(0.06,6.35)	2.32(0.08,7.80)	0.87(0.84,0.90)
Cambodia	30.28(1.65,86.94)	63.55(3.10,190.15)	2.44(2.17,2.72)	1.65(0.06,5.62)	3.71(0.12,13.03)	2.75(2.49,3.01)
Cameroon	30.55(1.70,87.90)	43.61(2.27,131.32)	1.28(1.22,1.35)	1.84(0.07,6.19)	2.51(0.09,8.96)	1.11(1.03,1.18)
Canada	33.07(1.94,89.51)	59.05(3.36,155.97)	1.83(1.71,1.95)	1.61(0.06,5.46)	3.00(0.11,9.91)	2.04(1.91,2.18)
Cape Verde	34.50(1.85,97.80)	54.44(2.90,153.59)	1.57(1.51,1.63)	1.96(0.07,6.60)	3.10(0.12,10.35)	1.54(1.50,1.57)
Central African Republic	60.63(3.54,165.47)	77.06(3.86,219.57)	0.70(0.68,0.73)	3.47(0.15,11.08)	4.24(0.16,14.00)	0.59(0.55,0.62)
Chad	28.19(1.56,74.95)	42.15(2.02,124.20)	1.27(1.19,1.35)	1.59(0.07,5.05)	2.39(0.08,7.83)	1.29(1.21,1.37)

Chile	35.05(2.09,94.95)	63.02(3.63,169.15)	1.97(1.86,2.09)	1.84(0.07,6.00)	3.35(0.13,10.76)	2.02(1.91,2.14)
China	57.10(3.15,157.09)	66.72(3.91,177.01)	0.57(0.41,0.74)	3.38(0.13,10.63)	3.64(0.14,11.53)	0.37(0.20,0.53)
Colombia	42.53(2.31,116.70)	57.46(3.18,155.51)	0.62(0.50,0.74)	2.11(0.07,6.93)	2.90(0.11,9.43)	0.66(0.53,0.79)
Comoros	41.06(1.91,128.15)	51.50(2.25,160.83)	0.77(0.75,0.78)	2.46(0.08,8.43)	3.09(0.10,10.71)	0.77(0.75,0.79)
Congo	62.85(3.07,175.86)	82.71(3.89,251.85)	0.93(0.90,0.96)	3.80(0.14,12.33)	4.91(0.17,17.22)	0.86(0.83,0.88)
Cook Islands	92.62(5.63,258.58)	93.72(6.53,235.86)	0.04(0.00,0.07)	5.19(0.21,17.04)	5.06(0.27,15.45)	-0.07(-0.12,-0.02)
Costa Rica	44.66(2.52,122.12)	64.82(3.69,180.83)	1.08(0.95,1.20)	2.28(0.08,7.34)	3.31(0.12,11.04)	1.09(0.97,1.21)
Cote d'Ivoire	28.98(1.52,85.30)	44.14(2.08,134.96)	1.51(1.41,1.61)	1.75(0.06,5.81)	2.55(0.08,8.84)	1.35(1.26,1.44)
Croatia	44.72(2.68,119.48)	65.42(3.96,172.03)	1.37(1.29,1.46)	2.36(0.09,7.93)	3.48(0.14,11.05)	1.37(1.29,1.46)
Cuba	44.87(2.51,124.44)	55.22(3.11,147.60)	0.49(0.44,0.55)	2.36(0.09,8.09)	2.84(0.10,9.46)	0.49(0.44,0.54)
Cyprus	80.97(4.04,243.46)	73.94(3.87,209.59)	-0.39(-0.42,-0.35)	4.70(0.15,16.42)	4.21(0.15,13.89)	-0.48(-0.54,-0.43)
Czech Republic	43.46(2.61,120.01)	75.38(4.44,196.97)	1.89(1.83,1.94)	2.34(0.09,7.99)	4.04(0.16,13.07)	1.91(1.85,1.96)
Democratic Republic of the Congo	62.57(3.70,164.15)	81.30(4.42,226.27)	0.86(0.77,0.94)	3.60(0.16,11.30)	4.73(0.20,14.69)	0.90(0.79,1.01)
Denmark	22.95(1.27,67.24)	37.41(2.08,104.28)	1.58(1.49,1.67)	1.33(0.05,4.54)	2.31(0.09,7.25)	1.79(1.68,1.89)
Djibouti	32.38(1.59,92.61)	50.27(2.26,153.02)	1.57(1.52,1.61)	1.94(0.06,6.18)	2.95(0.10,9.95)	1.48(1.44,1.52)
Dominica	55.80(3.07,162.56)	64.56(3.79,178.23)	0.48(0.44,0.52)	2.79(0.10,9.53)	3.30(0.14,10.99)	0.55(0.50,0.60)
Dominican Republic	26.04(1.50,71.23)	38.72(2.29,102.31)	1.35(1.26,1.44)	1.37(0.06,4.58)	1.92(0.08,6.24)	1.26(1.15,1.37)
Ecuador	29.50(1.80,77.92)	49.07(2.87,131.29)	1.79(1.64,1.93)	1.52(0.06,4.85)	2.51(0.09,8.18)	1.78(1.64,1.93)
Egypt	36.00(2.02,98.32)	79.61(4.36,226.33)	2.98(2.84,3.12)	1.94(0.07,6.56)	4.24(0.16,14.15)	2.94(2.79,3.09)

El Salvador	32.21(1.85,86.68)	58.13(3.31,165.69)	1.79(1.72,1.85)	1.65(0.06,5.31)	2.89(0.11,9.83)	1.68(1.61,1.74)
Equatorial Guinea	55.57(3.02,160.55)	95.46(5.35,275.27)	1.96(1.88,2.04)	3.18(0.12,10.83)	5.46(0.20,18.54)	1.97(1.88,2.06)
Eritrea	26.67(1.19,78.23)	47.46(2.07,144.77)	1.98(1.90,2.06)	1.53(0.05,5.18)	2.79(0.08,9.57)	2.05(1.92,2.18)
Estonia	27.23(1.47,76.38)	47.70(2.71,128.87)	2.09(1.89,2.30)	1.43(0.05,4.82)	2.61(0.09,8.36)	2.24(2.05,2.43)
Ethiopia	42.09(2.13,115.19)	43.91(2.14,122.85)	0.15(0.05,0.24)	2.46(0.09,7.95)	2.66(0.10,8.34)	0.31(0.23,0.40)
Federated States of Micronesia	81.26(4.30,227.39)	90.79(4.72,257.57)	0.33(0.26,0.39)	4.39(0.17,14.78)	4.98(0.19,16.85)	0.39(0.33,0.45)
Fiji	83.29(4.89,228.18)	90.31(6.36,230.52)	0.18(0.14,0.23)	4.54(0.18,15.27)	4.91(0.24,15.38)	0.15(0.09,0.22)
Finland	58.74(3.40,161.44)	74.16(4.03,203.98)	0.86(0.80,0.92)	3.56(0.14,11.48)	4.45(0.18,14.36)	0.86(0.79,0.93)
France	26.69(1.62,74.76)	34.97(2.13,95.42)	0.88(0.86,0.89)	1.55(0.06,5.15)	2.02(0.08,6.44)	0.85(0.83,0.87)
Gabon	70.75(3.88,200.96)	96.10(5.33,273.60)	0.99(0.92,1.05)	4.26(0.17,13.64)	5.61(0.21,18.16)	0.86(0.80,0.92)
Georgia	23.72(1.24,65.06)	54.25(2.73,161.20)	3.39(3.14,3.63)	1.23(0.04,4.04)	2.87(0.10,10.13)	3.50(3.24,3.76)
Germany	58.19(3.23,152.13)	87.40(4.65,236.65)	1.32(1.23,1.41)	3.36(0.13,10.67)	5.10(0.19,16.22)	1.31(1.20,1.43)
Ghana	27.82(1.56,76.95)	46.43(2.51,128.66)	1.86(1.73,1.98)	1.63(0.06,5.53)	2.71(0.09,9.17)	1.83(1.73,1.94)
Greece	43.90(2.75,116.18)	53.47(3.28,138.11)	0.54(0.48,0.59)	2.48(0.10,7.93)	3.03(0.13,9.58)	0.55(0.50,0.60)
Greenland	21.66(1.06,58.43)	56.80(2.87,165.07)	3.17(2.98,3.37)	1.09(0.04,3.68)	3.11(0.12,10.28)	3.46(3.23,3.70)
Grenada	55.54(2.97,161.84)	62.57(3.33,172.57)	0.58(0.53,0.63)	2.91(0.10,10.12)	3.21(0.11,10.68)	0.66(0.57,0.75)
Guam	65.71(4.01,176.02)	77.73(5.05,196.97)	0.65(0.54,0.76)	3.57(0.15,11.33)	3.83(0.18,11.51)	0.36(0.22,0.51)
Guatemala	36.11(2.10,96.78)	64.14(3.61,178.31)	1.77(1.46,2.08)	1.81(0.07,6.03)	3.24(0.12,10.76)	1.78(1.47,2.09)
Guinea	24.02(1.31,63.64)	38.12(1.87,113.48)	1.23(1.07,1.40)	1.34(0.05,4.30)	2.13(0.07,7.34)	1.22(1.04,1.40)

Guinea-Bissau	29.13(1.47,82.99)	46.75(2.22,137.80)	1.67(1.59,1.75)	1.69(0.06,5.76)	2.67(0.08,9.41)	1.59(1.54,1.65)
Guyana	54.66(3.14,147.71)	66.26(4.16,175.16)	0.53(0.44,0.63)	2.68(0.09,8.95)	3.31(0.14,10.84)	0.58(0.44,0.71)
Haiti	49.97(2.67,136.31)	56.51(2.85,164.30)	0.46(0.44,0.49)	2.48(0.09,8.11)	2.94(0.10,10.12)	0.63(0.59,0.67)
Honduras	45.98(2.45,131.56)	73.23(3.98,208.01)	1.29(1.09,1.49)	2.37(0.08,7.68)	3.97(0.14,13.14)	1.44(1.20,1.67)
Hungary	47.69(2.71,132.78)	68.49(3.67,187.20)	0.96(0.78,1.14)	2.57(0.09,8.64)	3.63(0.13,12.32)	0.89(0.72,1.06)
Iceland	38.47(2.34,101.26)	54.79(3.43,146.82)	1.20(1.16,1.24)	2.10(0.09,6.61)	3.10(0.14,9.62)	1.34(1.30,1.39)
India	31.99(1.76,88.50)	50.63(2.71,141.50)	1.44(1.39,1.48)	1.68(0.06,5.62)	2.84(0.10,9.41)	1.74(1.68,1.80)
Indonesia	31.24(1.79,80.51)	57.38(3.00,164.98)	2.14(1.90,2.39)	1.56(0.06,4.91)	3.13(0.11,10.51)	2.44(2.11,2.77)
Iran	52.90(2.95,146.24)	84.59(4.70,233.13)	1.69(1.61,1.77)	2.85(0.11,9.24)	4.52(0.17,14.87)	1.66(1.58,1.74)
Iraq	71.84(4.34,191.97)	115.59(7.08,312.44)	1.68(1.63,1.72)	3.64(0.15,11.64)	6.15(0.25,19.64)	1.80(1.76,1.83)
Ireland	39.70(2.36,108.94)	51.84(3.08,140.13)	0.92(0.85,0.98)	2.27(0.09,7.39)	2.98(0.12,9.42)	0.97(0.90,1.04)
Israel	48.26(2.83,139.64)	77.72(4.40,214.90)	1.55(1.37,1.73)	2.67(0.10,9.10)	4.43(0.18,14.42)	1.66(1.49,1.84)
Italy	56.66(3.34,149.61)	69.32(4.28,177.43)	0.59(0.53,0.65)	3.48(0.14,10.80)	4.07(0.18,12.16)	0.53(0.45,0.60)
Jamaica	39.47(2.05,119.54)	45.65(2.47,132.18)	0.50(0.46,0.55)	1.92(0.06,6.62)	2.23(0.08,7.57)	0.55(0.50,0.59)
Japan	63.37(3.99,161.69)	67.66(4.29,175.19)	0.13(0.04,0.22)	3.79(0.17,11.47)	4.00(0.19,11.91)	0.08(0.02,0.14)
Jordan	87.96(5.29,237.11)	104.59(6.50,267.84)	0.54(0.51,0.57)	4.64(0.17,15.49)	5.34(0.22,16.64)	0.39(0.34,0.43)
Kazakhstan	31.70(1.72,88.63)	58.29(3.00,170.22)	2.23(2.10,2.36)	1.72(0.07,5.81)	3.08(0.10,10.41)	2.09(1.97,2.21)
Kenya	33.63(1.69,91.43)	43.26(2.19,122.41)	0.87(0.84,0.89)	1.99(0.07,6.39)	2.53(0.09,7.93)	0.85(0.82,0.89)
Kiribati	83.44(4.84,218.58)	106.00(6.41,290.38)	0.77(0.70,0.84)	4.33(0.19,14.03)	5.90(0.27,19.24)	1.01(0.93,1.08)

Kuwait	95.74(5.15,258.19)	97.71(6.25,247.88)	0.11(0.01,0.21)	5.17(0.19,17.24)	5.24(0.22,16.35)	0.08(-0.06,0.23)
Kyrgyzstan	21.88(1.25,61.36)	37.42(2.05,103.26)	1.54(1.49,1.59)	1.18(0.04,4.12)	1.94(0.07,6.65)	1.36(1.29,1.43)
Laos	53.38(2.76,149.91)	67.67(3.35,198.31)	0.71(0.63,0.78)	2.80(0.10,9.08)	3.70(0.12,12.90)	0.84(0.74,0.94)
Latvia	26.97(1.58,72.84)	44.54(2.53,122.70)	1.78(1.65,1.91)	1.44(0.05,4.69)	2.34(0.09,7.77)	1.73(1.61,1.85)
Lebanon	76.29(4.38,213.68)	95.35(5.93,253.96)	0.77(0.64,0.90)	3.98(0.13,13.41)	4.94(0.20,16.27)	0.78(0.61,0.95)
Lesotho	31.68(1.37,88.68)	56.83(2.61,169.92)	2.23(2.01,2.44)	1.75(0.05,5.69)	3.05(0.10,10.69)	2.14(1.90,2.38)
Liberia	32.03(1.57,91.01)	46.02(2.25,139.70)	1.32(1.24,1.39)	1.89(0.06,6.26)	2.65(0.09,9.33)	1.22(1.15,1.29)
Libya	75.38(3.65,222.10)	94.42(4.72,263.97)	0.83(0.71,0.95)	4.24(0.15,14.36)	5.29(0.18,17.30)	0.86(0.73,0.98)
Lithuania	25.60(1.46,69.27)	38.65(2.25,104.98)	1.43(1.33,1.53)	1.37(0.05,4.58)	2.08(0.08,6.76)	1.43(1.34,1.53)
Luxembourg	24.52(1.49,66.59)	55.63(3.08,156.58)	2.87(2.63,3.11)	1.32(0.06,4.48)	3.22(0.14,10.85)	3.13(2.89,3.36)
Macedonia	46.68(2.84,125.53)	73.80(4.13,197.71)	1.43(1.23,1.62)	2.39(0.09,7.93)	3.91(0.14,13.22)	1.54(1.33,1.75)
Madagascar	25.98(1.28,74.36)	32.96(1.64,92.67)	0.81(0.76,0.87)	1.44(0.05,4.88)	1.77(0.06,5.82)	0.71(0.67,0.76)
Malawi	27.47(1.37,77.75)	37.59(1.75,108.31)	0.97(0.92,1.02)	1.65(0.06,5.65)	2.17(0.07,7.60)	0.89(0.86,0.92)
Malaysia	69.67(4.17,188.49)	89.35(5.09,251.88)	0.75(0.65,0.84)	3.84(0.16,12.46)	5.03(0.19,16.44)	0.77(0.64,0.90)
Maldives	46.62(2.53,125.74)	65.61(3.49,185.38)	1.05(0.94,1.16)	2.41(0.09,7.87)	3.48(0.12,11.87)	1.13(0.98,1.29)
Mali	33.01(1.89,91.31)	44.96(2.31,129.04)	1.01(0.98,1.05)	1.94(0.07,6.32)	2.63(0.10,8.73)	1.01(0.97,1.06)
Malta	41.33(2.38,114.84)	68.29(3.74,180.77)	1.55(1.39,1.72)	2.28(0.10,7.44)	3.86(0.15,12.17)	1.60(1.44,1.77)
Marshall Islands	136.43(8.43,377.34)	146.14(9.39,398.94)	0.21(0.18,0.25)	7.98(0.33,25.50)	8.43(0.37,27.28)	0.16(0.12,0.19)
Mauritania	24.76(1.33,71.88)	33.06(1.71,94.09)	0.75(0.64,0.86)	1.43(0.05,5.00)	1.87(0.07,6.40)	0.64(0.52,0.76)

Mauritius	74.54(4.05,210.06)	79.88(4.57,213.12)	0.16(0.10,0.23)	4.07(0.14,14.00)	4.19(0.16,13.77)	0.00(-0.09,0.10)
Mexico	57.39(3.54,149.37)	56.77(3.50,152.20)	-0.08(-0.10,-0.07)	2.72(0.11,9.02)	2.73(0.11,8.76)	-0.10(-0.13,-0.07)
Moldova	30.50(1.68,81.12)	43.13(2.53,117.58)	1.39(1.20,1.58)	1.63(0.06,5.22)	2.21(0.09,7.35)	1.29(1.08,1.50)
Mongolia	18.56(1.05,50.84)	30.58(1.72,82.45)	1.65(1.56,1.74)	1.02(0.04,3.23)	1.64(0.06,5.43)	1.53(1.44,1.62)
Montenegro	50.22(3.15,131.48)	73.33(4.43,195.32)	1.34(1.27,1.41)	2.64(0.11,8.42)	3.93(0.16,12.85)	1.43(1.36,1.50)
Morocco	62.84(3.96,168.06)	115.06(6.67,320.83)	2.27(2.15,2.39)	3.31(0.14,10.88)	6.25(0.24,20.36)	2.38(2.25,2.50)
Mozambique	35.89(1.70,112.87)	48.96(2.03,152.37)	1.19(1.13,1.26)	2.25(0.08,8.00)	2.94(0.08,10.04)	1.05(0.98,1.12)
Myanmar	54.97(3.10,146.25)	73.88(3.68,206.70)	0.88(0.79,0.96)	2.75(0.09,9.01)	3.98(0.14,12.98)	1.11(0.99,1.22)
Namibia	44.01(2.21,127.39)	59.27(2.70,174.27)	1.12(1.04,1.20)	2.44(0.09,8.31)	3.21(0.10,11.22)	1.00(0.94,1.07)
Nepal	28.12(1.62,74.51)	51.40(2.56,149.82)	2.03(1.98,2.08)	1.46(0.05,4.82)	2.93(0.10,9.97)	2.41(2.34,2.47)
Netherlands	42.61(2.51,115.19)	52.98(3.04,140.69)	0.65(0.61,0.70)	2.49(0.10,7.99)	3.05(0.12,9.62)	0.62(0.56,0.67)
New Zealand	47.09(2.82,125.31)	61.78(4.03,160.81)	0.95(0.90,1.01)	2.60(0.12,8.39)	3.39(0.16,10.32)	0.94(0.87,1.00)
Nicaragua	47.13(2.43,136.67)	59.71(3.26,170.32)	0.72(0.68,0.77)	2.31(0.08,7.99)	2.94(0.10,9.93)	0.73(0.68,0.79)
Niger	27.28(1.53,72.60)	40.49(1.95,118.19)	1.37(1.31,1.43)	1.56(0.06,4.98)	2.33(0.08,7.95)	1.42(1.33,1.50)
Nigeria	23.76(1.21,65.46)	35.85(1.74,106.16)	1.46(1.34,1.58)	1.42(0.05,4.64)	2.17(0.07,7.28)	1.56(1.43,1.69)
North Korea	39.46(2.23,106.18)	54.68(2.91,147.98)	1.07(1.04,1.09)	2.19(0.09,7.29)	3.09(0.12,9.98)	1.16(1.12,1.20)
Northern Mariana Islands	73.50(4.71,203.64)	89.33(5.40,245.02)	0.56(0.53,0.60)	3.96(0.16,12.92)	4.91(0.20,16.25)	0.63(0.58,0.68)
Norway	57.76(3.55,151.87)	56.42(3.49,144.66)	-0.12(-0.20,-0.05)	3.32(0.14,10.51)	3.34(0.15,10.17)	-0.01(-0.07,0.05)
Oman	63.06(3.44,183.06)	90.38(5.30,256.39)	1.21(1.11,1.31)	3.34(0.13,11.57)	4.90(0.19,15.69)	1.34(1.23,1.45)

Pakistan	34.41(1.88,92.02)	60.01(3.22,169.21)	2.03(1.91,2.14)	1.88(0.07,5.98)	3.47(0.13,11.36)	2.20(2.08,2.33)
Palestine	71.48(3.79,201.49)	88.98(4.87,255.56)	0.71(0.62,0.81)	3.69(0.12,12.64)	4.73(0.17,16.33)	0.83(0.71,0.95)
Panama	38.37(2.22,102.69)	61.55(3.55,165.97)	1.47(1.40,1.55)	1.90(0.07,6.15)	3.09(0.13,10.09)	1.52(1.44,1.60)
Papua New Guinea	58.19(3.28,168.21)	70.88(3.61,203.67)	0.60(0.55,0.66)	2.95(0.11,10.04)	3.70(0.11,13.12)	0.71(0.67,0.75)
Paraguay	44.24(2.50,121.13)	75.13(4.29,208.00)	1.58(1.49,1.68)	2.32(0.09,7.34)	4.00(0.17,12.87)	1.64(1.54,1.73)
Peru	19.13(1.05,52.53)	33.64(1.94,87.92)	1.99(1.90,2.07)	1.03(0.04,3.49)	1.79(0.07,5.78)	1.98(1.90,2.07)
Philippines	50.54(2.53,148.58)	58.58(2.86,171.78)	0.50(0.48,0.53)	2.69(0.09,9.21)	3.17(0.11,11.14)	0.61(0.57,0.66)
Poland	51.04(3.13,134.69)	70.51(4.34,186.83)	1.11(1.06,1.16)	2.72(0.11,8.60)	3.82(0.16,12.12)	1.16(1.12,1.21)
Portugal	51.23(3.11,140.63)	77.94(4.62,214.67)	1.45(1.33,1.58)	2.93(0.12,9.66)	4.43(0.18,14.26)	1.43(1.31,1.54)
Principality of Monaco	41.53(2.46,115.92)	56.17(3.61,148.62)	0.98(0.96,1.00)	2.38(0.10,8.01)	3.39(0.16,10.54)	1.17(1.15,1.18)
Puerto Rico	60.92(3.59,160.24)	68.18(4.43,172.36)	0.34(0.30,0.38)	3.05(0.12,10.05)	3.41(0.15,10.13)	0.35(0.30,0.39)
Qatar	102.49(5.17,291.13)	113.14(7.55,290.53)	0.35(0.30,0.40)	5.91(0.20,20.13)	6.30(0.29,19.40)	0.20(0.14,0.27)
Republic of Nauru	85.57(4.58,252.88)	93.15(5.12,264.57)	0.26(0.24,0.27)	4.78(0.17,16.55)	5.19(0.19,17.63)	0.28(0.27,0.30)
Republic of Niue	92.33(5.26,261.23)	97.07(6.42,259.53)	0.11(0.10,0.13)	5.28(0.20,17.26)	5.51(0.25,17.11)	0.09(0.07,0.10)
Republic of Palau	89.02(5.44,254.51)	91.82(5.87,257.71)	0.08(0.07,0.10)	4.96(0.21,15.96)	5.06(0.21,17.41)	0.06(0.03,0.08)
Republic of San Marino	38.69(2.46,103.10)	50.16(2.92,135.87)	0.90(0.86,0.95)	2.15(0.09,6.73)	2.80(0.12,8.62)	0.98(0.90,1.05)
Romania	34.07(2.00,91.26)	51.81(3.32,139.62)	1.39(1.34,1.45)	1.81(0.07,5.90)	2.72(0.11,9.05)	1.34(1.29,1.40)
Russian Federation	27.48(1.55,76.44)	45.34(2.58,124.46)	1.85(1.77,1.93)	1.47(0.06,4.94)	2.40(0.09,7.69)	1.83(1.75,1.92)
Rwanda	32.08(1.46,94.76)	43.22(1.76,135.81)	1.01(0.96,1.05)	1.94(0.06,6.87)	2.56(0.07,9.31)	0.93(0.88,0.97)

Saint Kitts and Nevis	51.80(2.87,146.26)	59.18(3.37,158.84)	0.53(0.49,0.57)	2.62(0.09,9.19)	2.99(0.11,9.92)	0.61(0.54,0.67)
Saint Lucia	59.06(3.15,171.63)	63.10(3.64,171.76)	0.14(0.12,0.17)	3.08(0.11,11.16)	3.28(0.13,11.11)	0.08(0.05,0.12)
Saint Vincent and the Grenadines	60.13(3.15,173.74)	60.67(3.49,167.05)	0.16(0.09,0.22)	3.17(0.11,11.00)	3.08(0.12,10.35)	0.10(-0.01,0.20)
Samoa	86.22(4.48,245.77)	97.00(5.62,269.42)	0.43(0.39,0.47)	4.93(0.20,17.20)	5.58(0.22,18.31)	0.46(0.40,0.52)
Sao Tome and Principe	23.89(1.24,70.77)	39.50(2.06,114.73)	1.70(1.65,1.76)	1.43(0.05,4.98)	2.32(0.08,8.08)	1.64(1.58,1.70)
Saudi Arabia	77.77(3.85,239.28)	93.19(5.22,255.96)	0.49(0.43,0.54)	4.43(0.16,15.48)	5.31(0.20,17.50)	0.48(0.42,0.53)
Senegal	43.26(2.49,123.21)	61.71(3.10,183.29)	1.28(1.18,1.37)	2.55(0.10,8.59)	3.68(0.13,12.46)	1.30(1.22,1.38)
Serbia	47.17(2.72,127.31)	72.06(4.10,198.52)	1.35(1.20,1.50)	2.49(0.09,8.43)	3.81(0.14,12.81)	1.35(1.20,1.49)
Seychelles	65.10(3.73,174.14)	79.43(4.48,216.70)	0.47(0.37,0.58)	3.57(0.12,11.85)	4.22(0.16,13.93)	0.40(0.31,0.50)
Sierra Leone	24.53(1.25,72.00)	38.68(1.85,110.65)	1.64(1.58,1.70)	1.40(0.05,4.77)	2.14(0.07,7.24)	1.51(1.45,1.57)
Singapore	52.60(3.26,142.42)	54.18(3.41,138.78)	0.04(0.00,0.07)	2.91(0.12,9.50)	2.96(0.14,9.03)	0.02(-0.02,0.06)
Slovakia	41.27(2.58,111.70)	56.39(3.61,144.85)	1.00(0.95,1.05)	2.21(0.09,7.17)	3.02(0.14,9.62)	1.04(0.99,1.08)
Slovenia	44.47(2.77,121.59)	59.00(3.70,152.93)	0.90(0.76,1.05)	2.36(0.10,7.76)	3.21(0.14,9.74)	0.98(0.83,1.13)
Solomon Islands	50.20(2.56,146.74)	65.16(3.18,194.30)	0.83(0.72,0.94)	2.60(0.08,9.26)	3.52(0.12,12.38)	1.00(0.88,1.11)
Somalia	31.40(1.52,88.37)	44.54(2.07,129.79)	1.32(1.26,1.38)	1.80(0.06,6.02)	2.42(0.08,7.97)	1.18(1.12,1.24)
South Africa	46.93(2.29,134.26)	65.16(3.40,189.75)	1.30(1.17,1.42)	2.60(0.09,8.66)	3.60(0.13,12.48)	1.24(1.11,1.36)
South Korea	60.95(3.57,165.39)	69.36(4.22,173.22)	0.65(0.49,0.81)	3.67(0.15,11.72)	3.96(0.18,11.65)	0.50(0.36,0.64)
South Sudan	31.06(1.47,92.91)	40.24(1.90,118.98)	0.88(0.84,0.92)	1.83(0.06,6.68)	2.31(0.08,7.89)	0.80(0.77,0.83)
Spain	63.04(3.56,177.78)	73.54(4.04,197.78)	0.57(0.37,0.78)	3.58(0.15,11.98)	4.37(0.18,14.01)	0.73(0.51,0.95)

Sri Lanka	49.70(2.58,145.22)	65.21(3.48,179.33)	0.96(0.88,1.04)	2.58(0.09,9.10)	3.37(0.14,11.23)	0.96(0.88,1.04)
Sudan	59.31(3.14,166.41)	83.55(4.57,229.94)	1.06(0.98,1.14)	3.16(0.12,10.46)	4.44(0.17,14.70)	1.06(0.98,1.14)
Suriname	54.81(3.12,156.94)	63.70(3.70,179.00)	0.51(0.46,0.57)	2.72(0.10,9.11)	3.22(0.12,10.87)	0.58(0.52,0.65)
Swaziland	51.34(2.39,148.00)	70.73(3.33,207.97)	1.20(1.12,1.29)	2.93(0.10,9.88)	3.89(0.13,12.86)	1.12(1.02,1.21)
Sweden	39.87(2.34,106.90)	54.47(3.17,145.38)	1.13(1.06,1.20)	2.22(0.09,7.07)	3.02(0.13,9.27)	1.13(1.03,1.22)
Switzerland	52.05(2.90,143.11)	71.53(3.92,201.05)	0.96(0.84,1.08)	2.88(0.11,9.49)	4.17(0.16,13.51)	1.15(1.00,1.29)
Syria	59.47(3.41,166.16)	87.69(4.85,248.67)	1.29(1.18,1.40)	3.09(0.12,10.48)	4.66(0.17,15.84)	1.38(1.27,1.49)
Taiwan (Province of China)	38.64(2.34,105.06)	61.72(3.54,165.01)	1.28(1.17,1.38)	2.19(0.09,7.01)	3.48(0.13,11.12)	1.06(0.90,1.21)
Tajikistan	29.29(1.73,79.17)	45.41(2.56,130.69)	1.49(1.43,1.54)	1.55(0.06,5.10)	2.48(0.09,8.31)	1.58(1.51,1.64)
Tanzania	28.72(1.52,84.78)	39.81(1.80,117.43)	1.04(1.00,1.09)	1.77(0.07,6.14)	2.32(0.08,8.12)	0.83(0.79,0.87)
Thailand	50.10(2.58,139.36)	66.15(3.56,187.77)	0.78(0.74,0.83)	2.89(0.11,9.59)	3.66(0.15,11.77)	0.55(0.49,0.62)
The Bahamas	46.75(2.75,125.14)	59.20(3.33,164.75)	0.80(0.73,0.87)	2.30(0.09,7.53)	2.99(0.11,10.08)	0.91(0.83,0.98)
The Gambia	27.76(1.43,79.03)	45.11(2.13,132.80)	1.64(1.59,1.69)	1.64(0.06,5.46)	2.61(0.08,8.79)	1.54(1.50,1.59)
Timor-Leste	28.22(1.57,72.99)	54.09(2.61,160.21)	1.89(1.56,2.23)	1.47(0.05,4.75)	2.96(0.09,10.42)	2.08(1.74,2.41)
Togo	23.46(1.38,66.56)	32.80(1.68,97.97)	1.14(1.11,1.17)	1.37(0.05,4.54)	1.90(0.07,6.59)	1.09(1.06,1.12)
Tokelau	112.94(6.45,309.03)	120.86(8.36,326.32)	0.22(0.19,0.24)	6.49(0.25,21.40)	6.83(0.34,21.60)	0.15(0.13,0.17)
Tonga	83.11(4.19,236.26)	92.43(5.53,257.58)	0.40(0.38,0.43)	4.64(0.17,15.56)	5.23(0.21,17.30)	0.48(0.45,0.52)
Trinidad and Tobago	57.59(3.15,155.38)	60.02(3.43,161.84)	0.23(0.19,0.28)	2.96(0.10,10.40)	3.02(0.12,9.88)	0.20(0.13,0.26)
Tunisia	72.98(3.60,216.82)	92.78(5.21,253.04)	0.73(0.66,0.80)	3.95(0.13,14.00)	4.96(0.21,16.03)	0.70(0.63,0.77)

Turkey	56.22(3.22,150.18)	87.80(5.22,226.21)	1.64(1.41,1.87)	3.04(0.12,9.88)	4.62(0.19,14.49)	1.59(1.31,1.87)
Turkmenistan	25.23(1.42,72.96)	41.66(2.34,111.08)	1.59(1.54,1.65)	1.35(0.05,4.47)	2.18(0.08,7.19)	1.49(1.40,1.57)
Tuvalu	71.90(3.48,201.46)	75.94(3.61,224.72)	0.12(0.09,0.15)	4.01(0.13,13.38)	4.29(0.14,14.90)	0.15(0.11,0.19)
Uganda	35.54(1.66,100.14)	51.07(2.28,158.19)	1.23(1.19,1.27)	2.08(0.07,6.89)	2.98(0.09,10.20)	1.21(1.17,1.24)
Ukraine	28.22(1.62,76.01)	37.94(2.18,102.38)	1.01(0.93,1.09)	1.51(0.06,4.81)	2.00(0.07,6.38)	0.96(0.89,1.03)
United Arab Emirates	90.93(4.80,266.43)	93.17(6.05,251.19)	0.19(0.08,0.30)	5.05(0.18,16.78)	5.27(0.22,17.07)	0.30(0.15,0.44)
United Kingdom	34.64(1.82,98.09)	55.35(2.88,161.45)	1.68(1.58,1.77)	2.00(0.07,6.62)	3.22(0.11,11.22)	1.72(1.63,1.82)
United States	59.49(3.67,155.31)	104.84(6.26,279.72)	2.09(1.88,2.31)	3.23(0.14,9.83)	6.01(0.25,19.17)	2.30(2.08,2.53)
Uruguay	27.90(1.61,75.69)	53.11(2.90,152.15)	2.15(2.07,2.22)	1.48(0.06,4.80)	2.90(0.11,9.54)	2.26(2.17,2.35)
Uzbekistan	21.57(1.20,59.57)	50.54(2.52,141.33)	3.19(2.98,3.40)	1.16(0.04,3.76)	2.62(0.08,8.79)	3.03(2.81,3.26)
Vanuatu	56.37(3.22,156.99)	79.05(4.19,226.70)	1.12(1.07,1.17)	3.05(0.11,10.33)	4.34(0.17,14.53)	1.16(1.13,1.20)
Venezuela	44.38(2.54,118.10)	65.60(3.89,182.85)	1.12(1.06,1.19)	2.22(0.09,7.08)	3.27(0.14,10.78)	1.13(1.07,1.20)
Vietnam	48.34(2.34,141.71)	65.38(3.06,197.43)	0.96(0.87,1.06)	2.75(0.09,9.43)	3.88(0.13,13.26)	1.11(1.01,1.22)
Virgin Islands, U.S.	61.60(3.39,176.64)	63.34(3.81,162.54)	0.12(0.08,0.15)	3.22(0.11,10.85)	3.19(0.14,10.18)	0.03(-0.01,0.07)
Yemen	47.29(2.55,124.33)	72.27(3.76,211.90)	1.66(1.52,1.80)	2.49(0.10,7.87)	3.89(0.13,12.85)	1.74(1.60,1.89)
Zambia	46.13(2.30,129.33)	59.91(2.93,181.61)	0.96(0.91,1.01)	2.68(0.09,8.96)	3.39(0.12,11.45)	0.89(0.83,0.95)
Zimbabwe	53.86(2.59,167.95)	58.91(2.75,180.07)	0.38(0.31,0.45)	3.22(0.10,11.27)	3.29(0.10,11.68)	0.17(0.08,0.26)

ASPR: Age-standardized prevalence rate; ASIR: Age-standardized incidence rate; ASYR: Age-standardized disability-adjusted life-years rate; ASDR: Age-standardized death rate;

UI: uncertainty intervals; CI: confidence interval; EAPC: estimated annual percentage change;

Supplementary table 6. The change high fasting plasma glucose contributed to alzheimer's disease and other dementias burden by sex in GBD regions from 1990 to 2021

Location	Number×10³ (95% UI)		EAPC of number (95% CI)	PAF (%) (95% UI)		EAPC of ASR (95% CI)	ASR (per 100,000 population) (95% UI)		EAPC of ASR (95% CI)
	1990	2021		1990	2021		1990	2021	
**Female**									
				**DALYs**					
High SDI	386.97(23.17,1016.46)	1230.59(72.61,3285.99)	3.90(3.81,3.99)	10.30(0.90,20.23)	15.54(1.28,30.74)	1.42(1.31,1.52)	53.60(3.21,140.55)	80.12(4.74,211.61)	1.38(1.27,1.48)
middle SDI	244.67(14.11,668.08)	820.05(49.11,2165.51)	4.07(3.99,4.16)	9.79(0.82,19.68)	13.10(1.09,26.04)	1.08(0.95,1.20)	49.14(2.83,134.02)	69.86(4.18,183.97)	1.20(1.07,1.32)
Middle SDI	204.87(11.10,562.05)	893.60(51.31,2418.27)	4.91(4.85,4.97)	10.92(0.90,22.68)	13.70(1.12,27.97)	0.88(0.80,0.96)	54.00(2.92,149.19)	70.76(4.06,191.57)	0.88(0.80,0.95)
middle SDI	80.50(4.36,215.73)	376.65(20.23,1017.03)	5.20(5.17,5.23)	10.39(0.84,21.37)	15.52(1.25,31.68)	1.33(1.29,1.37)	39.95(2.17,106.25)	63.39(3.41,173.52)	1.52(1.48,1.55)
Low SDI	25.57(1.36,70.06)	95.63(4.86,269.31)	4.45(4.39,4.52)	8.77(0.71,18.10)	12.01(0.95,24.82)	1.04(1.01,1.08)	37.29(1.99,101.63)	54.86(2.79,153.91)	1.29(1.27,1.32)
Andean Latin America	2.25(0.12,6.00)	13.02(0.72,35.09)	1.94(1.82,2.06)	8.18(0.67,17.03)	14.34(1.11,29.33)	1.85(1.72,1.98)	25.05(1.38,66.93)	43.23(2.40,116.38)	6.17(6.01,6.32)
Australasia	6.23(0.40,16.59)	21.06(1.29,54.30)	1.27(1.21,1.33)	8.71(0.78,17.34)	12.57(1.17,23.41)	1.03(0.96,1.09)	44.49(2.85,119.13)	59.86(3.72,154.70)	3.97(3.85,4.10)
Caribbean	5.85(0.34,15.77)	17.89(1.04,46.89)	0.61(0.57,0.64)	14.00(1.09,28.33)	17.03(1.39,34.51)	0.48(0.44,0.51)	48.49(2.76,129.91)	57.45(3.35,149.57)	3.55(3.49,3.62)
Central Asia	7.18(0.39,19.57)	21.48(1.13,62.37)	2.71(2.53,2.89)	6.69(0.54,13.94)	13.65(1.01,29.03)	2.62(2.44,2.80)	27.90(1.52,77.35)	55.68(2.91,161.89)	3.89(3.65,4.13)
Central Europe	38.20(2.34,102.01)	109.87(6.70,284.73)	1.29(1.25,1.33)	11.36(0.97,22.08)	16.76(1.41,33.02)	1.26(1.21,1.30)	48.23(2.94,128.87)	70.34(4.28,181.65)	3.68(3.60,3.77)
Central Latin America	19.52(1.17,51.60)	85.53(5.09,231.51)	0.45(0.41,0.50)	14.73(1.18,29.66)	17.69(1.41,35.98)	0.37(0.33,0.42)	56.49(3.34,149.38)	65.71(3.91,177.85)	4.88(4.83,4.92)
Central Sub-Saharan Africa	3.90(0.22,10.27)	15.81(0.85,44.02)	0.72(0.67,0.77)	9.78(0.86,19.40)	12.40(1.01,25.23)	0.94(0.85,1.03)	65.03(3.69,172.44)	86.93(4.60,247.34)	4.56(4.52,4.60)
East Asia	190.25(10.48,526	802.24(47.99,2135.	0.59(0.42,0.76	10.57(0.91,21.	11.84(1.00,23.6	0.58(0.41,0.74)	62.71(3.46,172.6	74.37(4.44,197.36)	4.77(4.63,4.92)

	.08)	07)	)	29)	2)		5)		
Eastern Europe	49.09(2.83,134.83)	116.49(6.70,318.01)	1.72(1.64,1.80)	6.65(0.52,13.45)	10.73(0.88,21.51)	1.70(1.61,1.78)	28.42(1.62,78.80)	45.67(2.63,124.21)	3.03(2.87,3.19)
Eastern Sub-Saharan Africa	8.04(0.41,22.39)	27.56(1.32,79.07)	0.62(0.59,0.66)	7.08(0.55,15.17)	8.47(0.64,17.98)	0.84(0.79,0.89)	37.07(1.88,102.02)	47.06(2.25,134.59)	4.20(4.07,4.34)
High-income Asia Pacific	74.32(4.73,189.77)	301.99(19.02,761.07)	0.03(-0.07,0.14)	13.01(1.18,24.93)	13.76(1.23,26.53)	0.06(-0.03,0.16)	69.30(4.38,177.91)	72.16(4.60,177.29)	4.62(4.40,4.84)
High-income North America	138.86(8.56,359.95)	457.41(26.59,1236.33)	2.30(2.13,2.47)	10.21(0.95,19.43)	19.07(1.55,38.30)	2.20(2.01,2.39)	57.83(3.55,150.40)	105.85(6.19,281.91)	4.10(3.94,4.26)
North Africa and Middle East	37.68(2.13,102.67)	169.95(10.08,444.76)	1.83(1.78,1.88)	11.35(0.92,23.32)	19.17(1.58,38.41)	1.57(1.50,1.63)	65.13(3.69,178.58)	102.00(6.04,269.18)	5.24(5.16,5.32)
Oceania	0.54(0.03,1.52)	1.85(0.11,4.84)	0.89(0.84,0.93)	14.44(1.17,29.74)	18.75(1.51,38.12)	0.57(0.53,0.61)	70.74(3.99,195.12)	84.77(4.74,226.57)	3.93(3.83,4.03)
South Asia	60.96(3.22,164.70)	338.23(17.79,936.75)	1.14(1.09,1.20)	11.32(0.93,23.63)	16.23(1.32,32.99)	1.53(1.49,1.56)	35.11(1.87,95.86)	56.71(2.99,158.50)	5.80(5.76,5.83)
Southeast Asia	47.76(2.55,128.70)	202.54(10.54,566.57)	1.11(1.05,1.17)	10.96(0.84,23.59)	15.09(1.13,32.17)	1.22(1.13,1.31)	49.86(2.60,135.69)	71.84(3.69,203.46)	4.79(4.73,4.86)
Southern Latin America	10.39(0.63,28.58)	36.87(2.21,95.31)	1.41(1.37,1.46)	9.82(0.82,19.74)	15.47(1.30,30.47)	1.33(1.28,1.37)	42.41(2.59,116.58)	64.72(3.87,167.47)	4.11(3.98,4.24)
Southern Sub-Saharan Africa	6.87(0.33,19.47)	19.87(1.03,57.28)	1.19(1.11,1.27)	11.82(0.88,26.15)	15.95(1.16,33.94)	1.19(1.09,1.30)	55.22(2.66,157.08)	74.44(3.86,216.84)	3.64(3.52,3.75)
Tropical Latin America	26.10(1.42,71.41)	115.96(6.67,310.25)	0.78(0.72,0.84)	12.12(0.97,25.46)	14.77(1.23,29.32)	0.68(0.64,0.73)	68.14(3.77,188.72)	81.01(4.66,216.80)	5.09(5.03,5.15)
Western Europe	201.11(11.55,539.55)	513.25(28.85,1387.40)	1.12(1.07,1.18)	9.85(0.81,20.15)	13.94(1.10,28.71)	1.06(0.99,1.12)	49.74(2.86,133.14)	68.94(3.90,186.86)	3.09(3.05,3.13)
Western Sub-Saharan Africa	8.60(0.47,23.63)	30.85(1.56,88.88)	1.56(1.51,1.62)	7.61(0.61,15.66)	11.88(0.91,24.84)	1.82(1.73,1.91)	27.51(1.49,75.44)	45.54(2.29,134.49)	4.35(4.25,4.46)
**Death**									
High SDI	21.66(0.91,67.73)	77.38(3.26,239.14)	4.28(4.19,4.38	10.21(0.89,19.	15.53(1.27,30.6	1.45(1.35,1.56)	3.00(0.13,9.43)	4.51(0.19,13.97)	1.40(1.30,1.51)

			)	97)	6)				
middle SDI	12.37(0.49,38.77)	45.04(1.84,139.99)	4.40(4.30,4.49)	9.86(0.82,19.90)	13.10(1.08,26.09)	1.05(0.92,1.18)	2.76(0.11,8.76)	3.79(0.15,11.75)	1.12(0.98,1.27)
Middle SDI	9.52(0.36,30.33)	44.96(1.76,143.57)	5.24(5.17,5.30)	10.90(0.89,22.74)	13.63(1.11,27.85)	0.87(0.78,0.95)	2.94(0.11,9.36)	3.77(0.15,12.14)	0.85(0.78,0.93)
middle SDI	3.65(0.13,11.69)	18.93(0.70,60.64)	5.59(5.55,5.64)	10.52(0.85,21.74)	15.50(1.24,31.54)	1.29(1.25,1.34)	2.13(0.08,6.71)	3.52(0.13,11.44)	1.68(1.64,1.73)
Low SDI	1.14(0.04,3.72)	4.65(0.17,14.93)	4.80(4.73,4.87)	8.83(0.71,18.31)	11.79(0.92,24.20)	0.97(0.93,1.02)	2.05(0.08,6.53)	3.12(0.11,10.25)	1.45(1.40,1.50)
Andean Latin America	0.11(0.00,0.36)	0.67(0.03,2.17)	1.97(1.83,2.10)	8.09(0.66,16.95)	14.29(1.07,29.41)	1.85(1.71,1.99)	1.28(0.05,4.16)	2.20(0.08,7.18)	6.43(6.28,6.59)
Australasia	0.33(0.01,1.06)	1.28(0.06,3.84)	1.21(1.16,1.27)	8.68(0.77,17.12)	12.32(1.14,23.05)	1.14(1.08,1.21)	2.41(0.10,7.71)	3.34(0.15,10.05)	4.48(4.31,4.65)
Caribbean	0.27(0.01,0.88)	0.94(0.04,3.04)	0.61(0.57,0.65)	14.03(1.10,28.50)	17.12(1.40,34.79)	0.50(0.46,0.54)	2.42(0.09,8.02)	2.85(0.11,9.21)	4.03(3.93,4.13)
Central Asia	0.36(0.01,1.19)	1.06(0.04,3.58)	2.67(2.48,2.86)	6.70(0.54,13.95)	13.50(0.99,28.72)	2.54(2.35,2.74)	1.47(0.06,4.98)	2.88(0.10,9.81)	3.79(3.52,4.05)
Central Europe	1.83(0.07,5.91)	6.08(0.25,19.15)	1.29(1.26,1.33)	11.49(0.98,22.20)	16.96(1.42,33.22)	1.27(1.23,1.31)	2.55(0.10,8.30)	3.73(0.15,11.70)	4.17(4.04,4.30)
Central Latin America	0.84(0.03,2.71)	4.17(0.17,13.61)	0.50(0.45,0.55)	14.04(1.11,28.74)	17.25(1.36,35.33)	0.40(0.34,0.46)	2.70(0.10,8.78)	3.21(0.13,10.49)	5.27(5.20,5.34)
Central Sub-Saharan Africa	0.16(0.01,0.50)	0.75(0.03,2.39)	0.63(0.59,0.68)	9.84(0.87,19.40)	12.20(1.00,24.65)	0.99(0.88,1.09)	3.67(0.16,11.17)	4.98(0.20,16.29)	5.15(5.09,5.20)
East Asia	9.00(0.37,29.12)	40.63(1.60,128.45)	0.56(0.39,0.74)	10.60(0.90,21.41)	11.75(0.99,23.63)	0.37(0.21,0.54)	3.65(0.14,11.50)	3.97(0.16,12.65)	5.09(4.94,5.24)
Eastern Europe	2.34(0.09,7.56)	6.22(0.23,19.50)	1.72(1.63,1.80)	6.62(0.51,13.56)	10.66(0.86,21.34)	1.68(1.59,1.76)	1.49(0.06,4.91)	2.38(0.09,7.43)	3.39(3.13,3.66)
Eastern Sub-Saharan Africa	0.38(0.01,1.24)	1.39(0.05,4.52)	0.47(0.44,0.50)	7.26(0.55,15.48)	8.31(0.62,17.70)	0.87(0.82,0.92)	2.13(0.08,6.87)	2.73(0.10,9.11)	4.44(4.32,4.56)
High-income Asia Pacific	4.17(0.19,12.56)	20.84(1.02,61.13)	0.10(0.01,0.19)	13.16(1.18,25.09)	14.12(1.25,27.16)	0.01(-0.05,0.07)	4.14(0.19,12.37)	4.25(0.21,12.44)	5.29(5.09,5.50)

High-income North America	7.75(0.34,23.30)	27.61(1.12,89.45)	2.42(2.24,2.60)	9.97(0.93,18.93)	19.19(1.53,38.66)	2.36(2.17,2.56)	3.11(0.14,9.42)	5.92(0.24,18.67)	4.41(4.22,4.61)
North Africa and Middle East	1.75(0.07,5.63)	7.94(0.32,25.01)	1.83(1.78,1.88)	11.16(0.89,22.85)	18.88(1.55,37.92)	1.56(1.47,1.64)	3.43(0.14,10.95)	5.35(0.21,16.95)	5.39(5.28,5.50)
Oceania	0.02(0.00,0.07)	0.08(0.00,0.26)	0.88(0.85,0.90)	14.48(1.16,29.54)	18.69(1.50,37.84)	0.50(0.49,0.51)	3.74(0.14,12.55)	4.40(0.16,14.32)	4.21(4.09,4.34)
South Asia	2.68(0.09,8.70)	17.14(0.62,55.45)	1.09(1.03,1.15)	11.70(0.95,24.45)	16.44(1.33,33.64)	1.78(1.73,1.83)	1.87(0.06,6.14)	3.21(0.11,10.43)	6.33(6.27,6.39)
Southeast Asia	2.28(0.08,7.35)	10.29(0.38,34.05)	1.06(0.99,1.13)	11.44(0.86,24.36)	15.45(1.16,33.06)	1.30(1.19,1.42)	2.67(0.10,8.64)	3.98(0.15,13.22)	5.02(4.94,5.09)
Southern Latin America	0.52(0.02,1.71)	2.08(0.08,6.47)	1.43(1.39,1.48)	9.67(0.80,19.47)	15.30(1.28,29.88)	1.37(1.32,1.41)	2.27(0.09,7.41)	3.49(0.14,10.90)	4.52(4.35,4.70)
Southern Sub-Saharan Africa	0.36(0.01,1.17)	0.98(0.03,3.35)	1.01(0.95,1.07)	12.24(0.89,26.73)	15.80(1.14,33.56)	1.11(0.99,1.23)	3.05(0.11,10.12)	4.08(0.14,13.88)	3.45(3.34,3.56)
Tropical Latin America	1.25(0.05,3.94)	6.23(0.27,19.08)	0.77(0.70,0.83)	11.82(0.94,24.49)	14.34(1.20,28.38)	0.69(0.63,0.75)	3.65(0.14,11.44)	4.29(0.19,13.11)	5.55(5.47,5.64)
Western Europe	11.57(0.46,36.43)	33.19(1.35,103.50)	1.13(1.07,1.18)	9.85(0.81,20.14)	13.95(1.10,28.47)	1.08(1.01,1.14)	2.87(0.12,9.13)	3.99(0.16,12.41)	3.46(3.40,3.52)
Western Sub-Saharan Africa	0.43(0.02,1.39)	1.56(0.06,5.18)	1.43(1.38,1.48)	7.61(0.61,15.83)	11.45(0.87,23.92)	1.89(1.79,1.98)	1.58(0.06,5.14)	2.66(0.09,9.18)	4.44(4.35,4.53)
**Male**									
				**DALYs**					
High SDI	178.78(10.65,477.45)	680.39(40.37,1802.56)	4.62(4.54,4.70)	11.79(1.04,23.49)	17.57(1.48,34.55)	1.41(1.27,1.55)	46.63(2.79,124.98)	67.74(4.02,180.00)	1.33(1.21,1.44)
middle SDI	113.14(6.47,315.16)	417.55(24.02,1111.70)	4.51(4.41,4.61)	10.37(0.89,20.73)	13.73(1.16,27.50)	1.10(0.96,1.23)	40.27(2.32,109.65)	55.55(3.18,147.43)	1.16(1.04,1.29)
Middle SDI	123.05(6.72,341.32)	517.51(28.85,1401.85)	4.84(4.77,4.91)	11.47(0.96,23.64)	14.12(1.15,29.04)	0.81(0.73,0.89)	40.93(2.25,114.66)	53.36(2.96,143.44)	0.89(0.82,0.97)
middle SDI	62.16(3.53,166.52)	245.47(13.24,699.71)	4.60(4.57,4.63)	11.07(0.92,22.80)	16.36(1.32,33.81)	1.30(1.27,1.33)	31.62(1.78,85.73)	49.93(2.69,142.12)	1.52(1.49,1.55)

Low SDI	20.11(1.11,55.19)	66.46(3.38,186.82)	3.96(3.89,4.02)	10.89(0.88,22.62)	14.46(1.12,30.67)	0.88(0.86,0.90)	31.37(1.72,87.68)	43.27(2.20,124.00)	1.04(0.98,1.09)
Andean Latin America	1.67(0.10,4.61)	8.91(0.53,24.10)	1.99(1.90,2.09)	8.46(0.70,17.34)	14.80(1.17,31.00)	1.85(1.75,1.94)	21.10(1.20,57.44)	35.54(2.09,95.95)	6.05(5.89,6.22)
Australasia	2.95(0.19,8.04)	12.07(0.78,32.93)	0.98(0.95,1.01)	10.74(0.97,20.42)	14.19(1.29,26.75)	0.88(0.86,0.91)	35.30(2.15,96.37)	45.17(2.93,123.65)	4.72(4.68,4.76)
Caribbean	4.49(0.25,12.49)	12.20(0.70,33.07)	0.66(0.64,0.67)	14.42(1.13,29.95)	17.66(1.41,35.66)	0.51(0.50,0.53)	42.61(2.36,120.32)	50.63(2.88,137.19)	3.24(3.20,3.28)
Central Asia	2.82(0.16,7.69)	9.50(0.50,27.22)	2.35(2.26,2.43)	6.64(0.56,13.61)	12.67(0.94,26.41)	2.32(2.24,2.41)	22.23(1.25,60.58)	42.23(2.16,124.02)	4.38(4.17,4.60)
Central Europe	17.87(1.09,48.27)	50.92(2.99,132.85)	1.43(1.36,1.50)	11.34(0.97,22.41)	17.37(1.44,34.71)	1.37(1.29,1.44)	38.48(2.31,102.62)	57.90(3.38,153.04)	3.69(3.59,3.79)
Central Latin America	12.97(0.79,34.82)	51.60(2.99,141.55)	0.58(0.54,0.63)	13.88(1.14,28.45)	17.24(1.35,35.95)	0.47(0.42,0.52)	41.90(2.49,110.86)	50.53(2.93,136.82)	4.57(4.50,4.63)
Central Sub-Saharan Africa	2.91(0.17,7.89)	8.48(0.46,23.86)	0.57(0.54,0.59)	14.59(1.24,28.61)	17.37(1.45,35.60)	0.75(0.71,0.79)	56.98(3.29,156.24)	71.13(3.76,204.65)	3.51(3.39,3.62)
East Asia	104.49(5.73,299.51)	444.38(25.85,1205.69)	0.65(0.47,0.83)	10.67(0.96,21.72)	12.00(1.04,24.32)	0.72(0.56,0.88)	45.45(2.51,127.37)	54.86(3.10,146.57)	4.93(4.78,5.09)
Eastern Europe	14.60(0.82,40.51)	39.70(2.17,110.49)	1.65(1.54,1.77)	7.16(0.56,14.79)	10.86(0.87,22.36)	1.61(1.50,1.73)	24.43(1.35,67.31)	36.63(2.01,101.64)	3.69(3.50,3.88)
Eastern Sub-Saharan Africa	5.88(0.30,16.83)	18.10(0.84,53.46)	0.63(0.61,0.65)	9.64(0.71,21.51)	11.79(0.84,25.69)	0.76(0.74,0.78)	31.38(1.54,93.99)	39.75(1.83,116.86)	3.68(3.55,3.82)
High-income Asia Pacific	32.56(2.00,84.56)	149.65(9.31,391.42)	0.45(0.37,0.54)	14.21(1.31,27.22)	16.70(1.46,32.01)	0.54(0.48,0.61)	51.58(3.14,135.92)	61.00(3.79,159.78)	5.16(5.05,5.27)
High-income North America	74.51(4.57,194.37)	274.28(16.65,739.82)	1.97(1.74,2.21)	12.52(1.15,23.95)	21.26(1.87,41.21)	1.79(1.58,2.00)	56.64(3.48,149.28)	91.76(5.57,249.41)	4.56(4.40,4.71)
North Africa and Middle East	28.58(1.64,77.67)	130.23(7.42,348.35)	1.78(1.72,1.85)	11.63(0.96,23.46)	19.08(1.54,38.63)	1.51(1.45,1.56)	53.31(3.02,145.94)	80.79(4.59,219.95)	5.36(5.26,5.46)
Oceania	0.49(0.03,1.38)	1.45(0.08,4.04)	0.30(0.30,0.31)	17.58(1.41,36.73)	19.25(1.56,39.18)	0.10(0.08,0.13)	63.54(3.59,183.53)	66.29(3.62,190.11)	3.54(3.51,3.57)

South Asia	54.30(3.10,145.18)	231.07(12.69,656.45)	0.99(0.95,1.03)	12.25(1.03,25.20)	16.86(1.38,34.41)	1.30(1.24,1.36)	29.24(1.65,79.84)	44.30(2.38,126.81)	4.79(4.71,4.86)
Southeast Asia	26.44(1.47,71.50)	107.35(5.62,302.80)	1.05(0.98,1.12)	11.24(0.90,23.75)	15.59(1.16,33.04)	1.14(1.04,1.24)	36.94(2.00,102.86)	53.10(2.77,153.30)	4.50(4.44,4.57)
Southern Latin America	4.64(0.27,12.47)	17.12(0.96,49.18)	1.79(1.74,1.84)	9.63(0.81,19.32)	16.47(1.30,33.33)	1.71(1.65,1.77)	28.75(1.69,77.34)	48.02(2.71,137.05)	4.32(4.23,4.41)
Southern Sub-Saharan Africa	2.49(0.13,7.20)	6.70(0.34,19.48)	1.14(1.05,1.22)	11.21(0.83,24.97)	14.85(1.09,31.81)	1.16(1.06,1.26)	33.25(1.66,98.49)	44.57(2.24,133.23)	3.43(3.38,3.47)
Tropical Latin America	15.78(0.89,44.35)	69.41(3.92,189.21)	1.12(1.06,1.18)	11.81(0.97,24.48)	15.65(1.28,31.73)	1.11(1.05,1.16)	52.70(3.00,146.59)	69.60(3.93,191.59)	5.15(5.07,5.23)
Western Europe	81.06(4.68,221.83)	266.25(14.87,733.58)	1.20(1.13,1.28)	10.48(0.86,21.60)	15.10(1.19,31.38)	1.18(1.11,1.25)	39.22(2.27,107.61)	55.85(3.11,153.39)	4.08(4.03,4.12)
Western Sub-Saharan Africa	6.34(0.33,18.03)	19.76(0.96,57.65)	0.95(0.90,1.00)	10.03(0.78,21.80)	13.57(1.01,29.06)	0.97(0.92,1.02)	25.12(1.29,70.94)	33.76(1.63,100.58)	3.83(3.76,3.90)
**Death**									
High SDI	8.89(0.37,28.34)	38.73(1.58,124.19)	5.09(5.00,5.17)	11.73(1.04,23.32)	17.65(1.48,34.71)	1.46(1.31,1.60)	2.64(0.11,8.64)	3.87(0.16,12.41)	1.36(1.24,1.49)
middle SDI	5.18(0.20,17.19)	20.92(0.85,67.95)	4.85(4.74,4.96)	10.48(0.91,20.78)	13.83(1.15,27.73)	1.07(0.94,1.21)	2.29(0.09,7.43)	3.09(0.12,9.93)	1.10(0.96,1.23)
Middle SDI	5.34(0.20,17.75)	24.38(0.95,78.10)	5.15(5.06,5.24)	11.62(0.97,24.09)	14.23(1.14,29.28)	0.78(0.70,0.87)	2.25(0.08,7.34)	2.91(0.11,9.26)	0.89(0.82,0.97)
middle SDI	2.75(0.10,8.91)	11.67(0.43,38.37)	4.90(4.85,4.95)	11.16(0.92,22.99)	16.38(1.31,33.67)	1.28(1.25,1.31)	1.69(0.06,5.51)	2.76(0.10,9.17)	1.69(1.65,1.73)
Low SDI	0.88(0.03,2.90)	3.11(0.11,10.14)	4.26(4.20,4.32)	11.35(0.90,23.73)	14.61(1.12,30.54)	0.79(0.77,0.81)	1.77(0.06,5.89)	2.47(0.09,8.20)	1.15(1.07,1.24)
Andean Latin America	0.08(0.00,0.27)	0.46(0.02,1.45)	2.00(1.91,2.10)	8.52(0.70,17.35)	14.94(1.16,31.23)	1.84(1.75,1.94)	1.13(0.04,3.75)	1.90(0.08,6.09)	6.25(6.07,6.42)
Australasia	0.14(0.01,0.45)	0.67(0.03,2.13)	0.93(0.91,0.96)	10.67(0.97,20.46)	13.93(1.28,26.31)	0.94(0.91,0.97)	1.94(0.08,6.32)	2.51(0.11,7.95)	5.33(5.27,5.39)
Caribbean	0.21(0.01,0.71)	0.64(0.02,2.07)	0.67(0.65,0.69)	14.43(1.14,30.07)	17.83(1.40,36.15)	0.55(0.53,0.57)	2.23(0.08,7.73)	2.67(0.10,8.65)	3.58(3.50,3.67)

Central Asia	0.13(0.01,0.44)	0.42(0.01,1.37)	2.26(2.18,2.35)	6.67(0.56,13.61)	12.47(0.92,25.83)	2.25(2.17,2.33)	1.20(0.05,3.96)	2.25(0.08,7.36)	4.19(3.88,4.51)
Central Europe	0.79(0.03,2.60)	2.55(0.10,8.30)	1.43(1.36,1.50)	11.34(0.96,22.36)	17.38(1.41,34.85)	1.37(1.30,1.43)	2.06(0.08,6.70)	3.11(0.12,10.29)	4.12(3.98,4.26)
Central Latin America	0.57(0.02,1.88)	2.47(0.10,8.16)	0.60(0.55,0.65)	13.50(1.11,27.63)	16.97(1.31,35.60)	0.49(0.43,0.54)	2.06(0.08,6.77)	2.52(0.10,8.27)	4.86(4.77,4.94)
Central Sub-Saharan Africa	0.12(0.00,0.41)	0.37(0.01,1.25)	0.47(0.46,0.49)	14.99(1.28,29.14)	17.42(1.46,35.82)	0.75(0.69,0.82)	3.37(0.13,11.00)	4.22(0.16,14.19)	3.63(3.55,3.72)
East Asia	4.42(0.17,14.70)	20.58(0.82,67.92)	0.60(0.41,0.78)	10.77(0.98,21.94)	11.92(1.02,24.08)	0.54(0.38,0.70)	2.68(0.10,8.65)	3.04(0.12,9.70)	5.27(5.09,5.44)
Eastern Europe	0.63(0.02,2.10)	1.96(0.07,6.54)	1.66(1.53,1.78)	7.26(0.57,14.91)	10.98(0.88,22.64)	1.59(1.46,1.72)	1.34(0.05,4.51)	1.99(0.08,6.70)	4.24(3.90,4.57)
Eastern Sub-Saharan Africa	0.28(0.01,0.94)	0.89(0.03,2.99)	0.46(0.45,0.47)	10.58(0.76,23.66)	12.20(0.85,26.71)	0.75(0.74,0.77)	1.88(0.06,6.71)	2.36(0.08,7.94)	3.84(3.73,3.95)
High-income Asia Pacific	1.64(0.07,5.20)	9.01(0.39,27.94)	0.45(0.38,0.52)	14.58(1.35,27.86)	17.09(1.48,32.38)	0.44(0.39,0.49)	3.03(0.13,9.41)	3.51(0.15,10.86)	5.71(5.65,5.76)
High-income North America	3.71(0.16,11.90)	15.52(0.66,50.36)	2.07(1.83,2.32)	12.31(1.13,23.58)	21.34(1.84,41.48)	2.00(1.76,2.24)	3.13(0.14,10.07)	5.34(0.23,17.34)	5.06(4.85,5.27)
North Africa and Middle East	1.32(0.05,4.30)	6.32(0.25,20.51)	1.78(1.71,1.84)	11.67(0.96,23.45)	19.06(1.53,38.76)	1.47(1.41,1.53)	2.96(0.11,9.67)	4.43(0.17,14.46)	5.64(5.50,5.77)
Oceania	0.02(0.00,0.06)	0.06(0.00,0.20)	0.31(0.31,0.32)	17.52(1.39,36.71)	19.26(1.54,38.93)	0.11(0.07,0.15)	3.43(0.13,11.64)	3.59(0.13,12.05)	3.86(3.80,3.92)
South Asia	2.35(0.08,7.84)	10.85(0.41,35.69)	0.94(0.90,0.99)	12.39(1.03,25.58)	16.83(1.36,34.15)	1.52(1.46,1.59)	1.55(0.05,5.02)	2.44(0.09,8.08)	5.14(5.06,5.21)
Southeast Asia	1.17(0.04,3.89)	5.09(0.19,16.92)	1.01(0.93,1.08)	11.89(0.94,25.30)	16.23(1.20,34.24)	1.22(1.11,1.34)	2.00(0.07,6.72)	2.96(0.11,10.03)	4.71(4.60,4.81)
Southern Latin America	0.21(0.01,0.70)	0.87(0.03,2.98)	1.85(1.79,1.90)	9.42(0.79,18.91)	16.39(1.28,33.40)	1.80(1.74,1.86)	1.49(0.06,4.87)	2.54(0.10,8.62)	4.74(4.60,4.87)
Southern Sub-Saharan Africa	0.12(0.00,0.40)	0.29(0.01,0.99)	0.96(0.90,1.03)	11.76(0.86,25.99)	14.93(1.08,31.74)	1.04(0.95,1.14)	1.85(0.06,6.27)	2.43(0.08,8.82)	3.11(3.05,3.16)

Tropical Latin America	0.70(0.03,2.30)	3.40(0.13,10.79)	1.13(1.07,1.19)	11.56(0.95,23.91)	15.39(1.25,31.22)	1.06(0.99,1.12)	2.82(0.11,9.19)	3.65(0.14,11.69)	5.51(5.39,5.63)
Western Europe	4.13(0.16,13.63)	15.50(0.59,50.80)	1.22(1.15,1.30)	10.41(0.85,21.31)	15.09(1.18,31.23)	1.22(1.15,1.29)	2.24(0.09,7.34)	3.21(0.12,10.54)	4.53(4.48,4.57)
Western Sub-Saharan Africa	0.31(0.01,1.04)	0.99(0.03,3.36)	0.79(0.74,0.83)	10.63(0.80,22.94)	13.70(1.00,29.17)	0.92(0.86,0.98)	1.52(0.05,5.18)	2.01(0.06,6.99)	3.97(3.88,4.05)

DALYs: disability-adjusted life-years; UI: uncertainty intervals; CI: confidence intervals; PAF: population attributable fraction; EAPC: estimated annual percentage change; GBD: Global Burden of Diseases; ASR: age-standardized rate;
